# Supplementary material for: Beyond predictive R2: Quantile regression and non-equivalence tests reveal complex relationships of traits and polygenic scores
Source: Am J Hum Genet. 2025 Jun 5;112(6):1363–75. doi: 10.1016/j.ajhg.2025.04.013 (PMC12256909; doi:10.1016/j.ajhg.2025.04.013)
Supplement: Document S1. Figures S1–S35 and Table S1 [file mmc1.pdf]

The American Journal of Human Genetics, Volume *112*

Supplemental information

Beyond predictive R^2: Quantile regression and non-equivalence tests reveal complex relationships of traits and polygenic scores

Joel Mefford, Molly Smullen, Felix Zhang, Michal Sadowski, Richard Border, Andy Dahl, Jonathan Flint, and Noah Zaitlen

Supplement

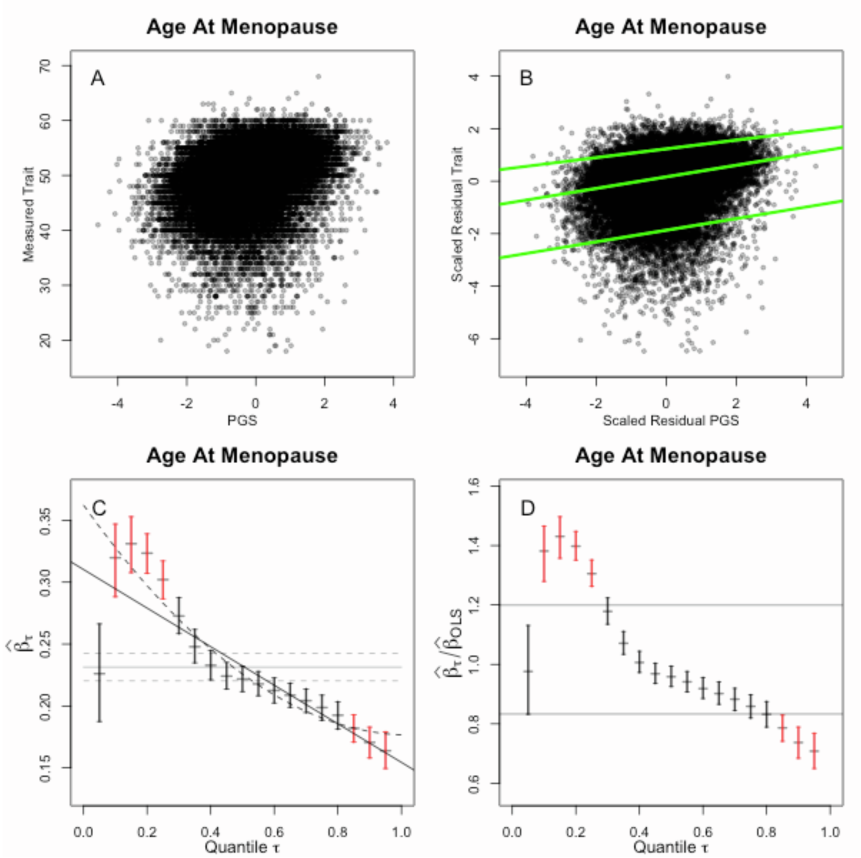

Figure S1. Age at menopause

Panels A and B show scatter plots of the trait (age at menopause) versus polygenic score for participants in the UK Biobank. In panel A and trait and PGS as reported in the data set are plotted. In panel B, residualized and scaled trait and PGS values are used, after projection of age, age^2, sex, age*sex, and ten genetic principal components and rescaling the residuals to mean zero variance one. The green lines show linear models fit to the data using QR at the 95th, 50th, and 5th quantiles of the residual trait distribution. QR is used to estimate quantile specific linear effect sizes $\widehat{\beta}_\tau$ at 19 quantiles τ of the residual trait distribution as in panel B. In panel C, $\widehat{\beta}_\tau$ is plotted against τ, with vertical bars representing 95% confidence intervals for $\widehat{\beta}_\tau$. The gray horizontal line with dashed lines above and below represent $\widehat{\beta}_{\mathrm{OLS}}$ and its 95% confidence interval for a linear model fit to residual data as in panel B. In panel D, $\widehat{\beta}_\tau/\widehat{\beta}_{\mathrm{OLS}}$ versus τ is plotted with vertical bars representing 95% confidence intervals for the ratio. Homogeneous effect sizes across the phenotypic distribution correspond to ratios $\widehat{\beta}_\tau/\widehat{\beta}_{\mathrm{OLS}}$ equal to one for each τ. The gray horizontal lines in panel D bound an equivalence band where the ratio is between $(1/\gamma, \gamma)$ for $\gamma = 1.2$. For ratios with the 95% confidence interval completely above or below the band, the vertical bars are colored red to indicate non-equivalence of the QR and OLS effect sizes and thus heterogeneity of $\widehat{\beta}_\tau$ and the linear predictive value of the PGS across the phenotypic range.

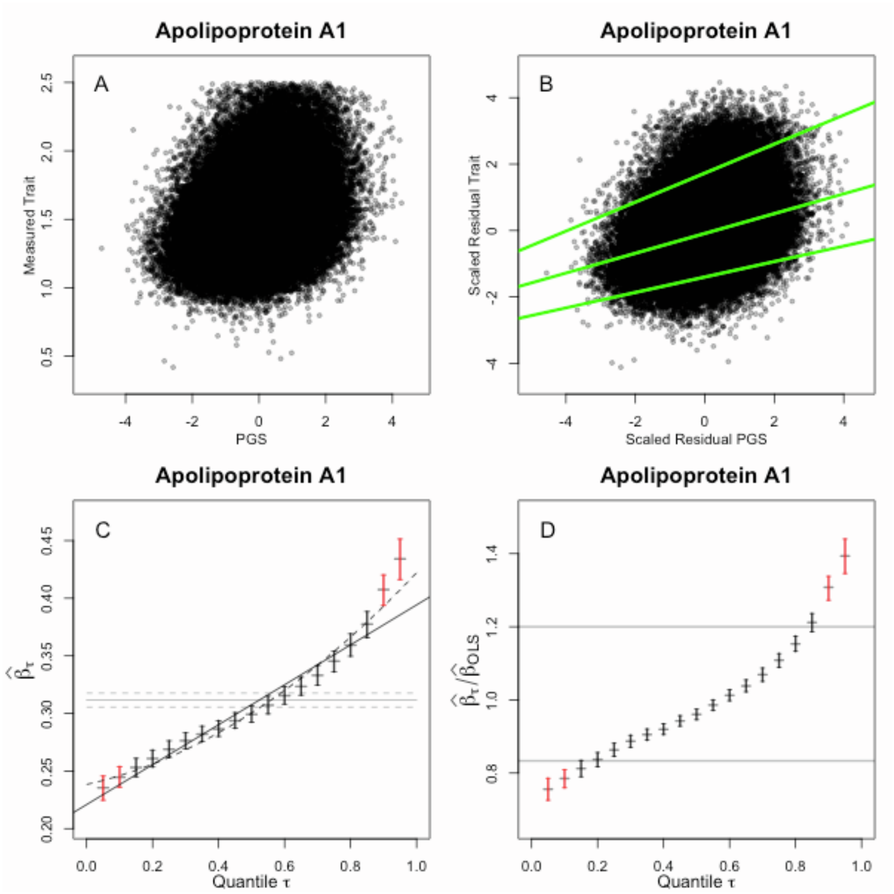

Figure S2. Apolipoprotein A1

Panels A and B show scatter plots of the trait (apolipoprotein A1) versus polygenic score for participants in the UK Biobank. In panel A and trait and PGS as reported in the data set are plotted. In panel B, residualized and scaled trait and PGS values are used, after projection of age, age^2, sex, age*sex, and ten genetic principal components and rescaling the residuals to mean zero variance one. The green lines show linear models fit to the data using QR at the 95th, 50th, and 5th quantiles of the residual trait distribution. QR is used to estimate quantile specific linear effect sizes $\widehat{\beta}_\tau$ at 19 quantiles τ of the residual trait distribution as in panel B. In panel C, $\widehat{\beta}_\tau$ is plotted against τ, with vertical bars representing 95% confidence intervals for $\widehat{\beta}_\tau$. The gray horizontal line with dashed lines above and below represent $\widehat{\beta}_{\mathrm{OLS}}$ and its 95% confidence interval for a linear model fit to residual data as in panel B. In panel D, $\widehat{\beta}_\tau/\widehat{\beta}_{\mathrm{OLS}}$ versus τ is plotted with vertical bars representing 95% confidence intervals for the ratio. Homogeneous effect sizes across the phenotypic distribution correspond to ratios $\widehat{\beta}_\tau/\widehat{\beta}_{\mathrm{OLS}}$ equal to one for each τ. The gray horizontal lines in panel D bound an equivalence band where the ratio is between $(1/\gamma, \gamma)$ for $\gamma = 1.2$. For ratios with the 95% confidence interval completely above or below the band, the vertical bars are colored red to indicate non-equivalence of the QR and OLS effect sizes and thus heterogeneity of $\widehat{\beta}_\tau$ and the linear predictive value of the PGS across the phenotypic range.

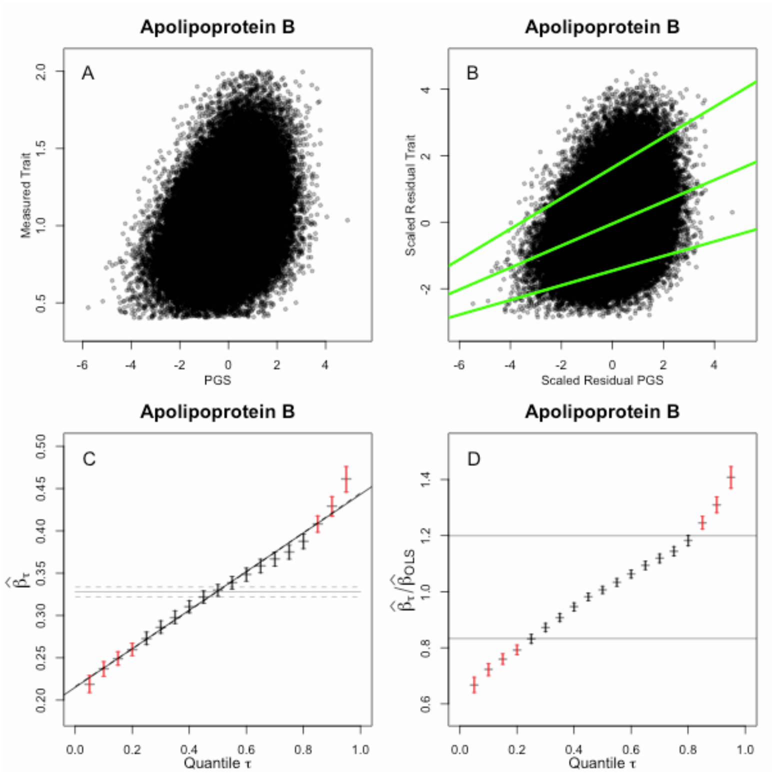

Figure S3. Apolipoprotein B

Panels A and B show scatter plots of the trait (apolipoprotein B) versus polygenic score for participants in the UK Biobank. In panel A and trait and PGS as reported in the data set are plotted. In panel B, residualized and scaled trait and PGS values are used, after projection of age, age^2, sex, age*sex, and ten genetic principal components and rescaling the residuals to mean zero variance one. The green lines show linear models fit to the data using QR at the 95th, 50th, and 5th quantiles of the residual trait distribution. QR is used to estimate quantile specific linear effect sizes $\widehat{\beta}_\tau$ at 19 quantiles τ of the residual trait distribution as in panel B. In panel C, $\widehat{\beta}_\tau$ is plotted against τ, with vertical bars representing 95% confidence intervals for $\widehat{\beta}_\tau$. The gray horizontal line with dashed lines above and below represent $\widehat{\beta}_{\mathrm{OLS}}$ and its 95% confidence interval for a linear model fit to residual data as in panel B. In panel D, $\widehat{\beta}_\tau/\widehat{\beta}_{\mathrm{OLS}}$ versus τ is plotted with vertical bars representing 95% confidence intervals for the ratio. Homogeneous effect sizes across the phenotypic distribution correspond to ratios $\widehat{\beta}_\tau/\widehat{\beta}_{\mathrm{OLS}}$ equal to one for each τ. The gray horizontal lines in panel D bound an equivalence band where the ratio is between $(1/\gamma, \gamma)$ for $\gamma = 1.2$. For ratios with the 95% confidence interval completely above or below the band, the vertical bars are colored red to indicate non-equivalence of the QR and OLS effect sizes and thus heterogeneity of $\widehat{\beta}_\tau$ and the linear predictive value of the PGS across the phenotypic range.

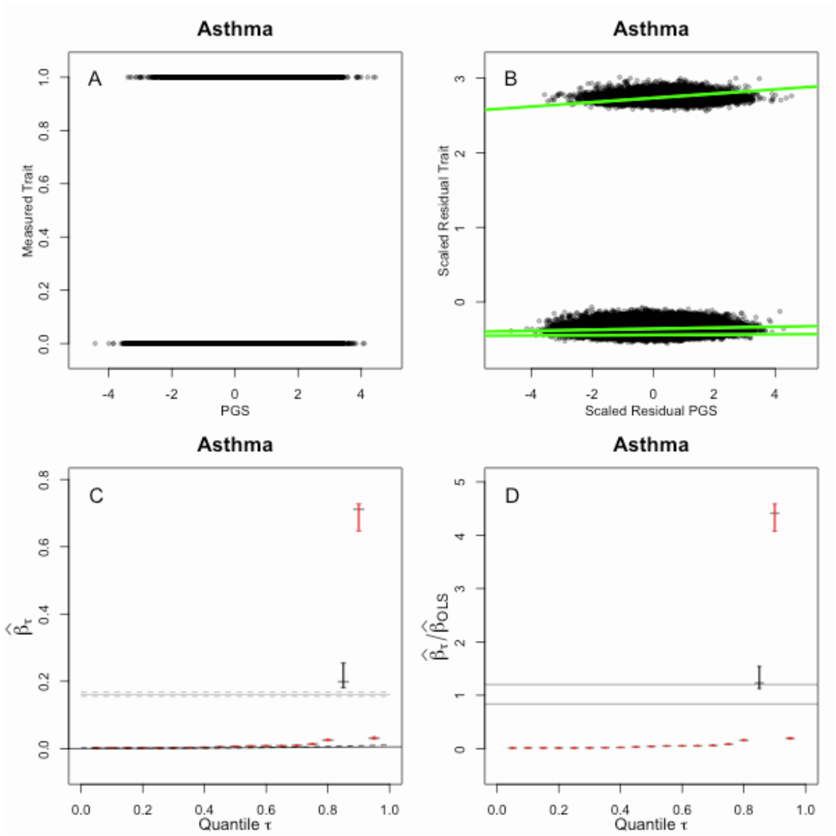

Figure S4. Asthma

Panels A and B show scatter plots of the trait (asthma) versus polygenic score for participants in the UK Biobank. In panel A and trait and PGS as reported in the data set are plotted. In panel B, residualized and scaled trait and PGS values are used, after projection of age, age^2, sex, age*sex, and ten genetic principal components and rescaling the residuals to mean zero variance one. The green lines show linear models fit to the data using QR at the 95th, 50th, and 5th quantiles of the residual trait distribution. QR is used to estimate quantile specific linear effect sizes $\widehat{\beta}_\tau$ at 19 quantiles τ of the residual trait distribution as in panel B. In panel C, $\widehat{\beta}_\tau$ is plotted against τ, with vertical bars representing 95% confidence intervals for $\widehat{\beta}_\tau$. The gray horizontal line with dashed lines above and below represent $\widehat{\beta}_{\mathrm{OLS}}$ and its 95% confidence interval for a linear model fit to residual data as in panel B. In panel D, $\widehat{\beta}_\tau/\widehat{\beta}_{\mathrm{OLS}}$ versus τ is plotted with vertical bars representing 95% confidence intervals for the ratio. Homogeneous effect sizes across the phenotypic distribution correspond to ratios $\widehat{\beta}_\tau/\widehat{\beta}_{\mathrm{OLS}}$ equal to one for each τ. The gray horizontal lines in panel D bound an equivalence band where the ratio is between $(1/\gamma, \gamma)$ for $\gamma = 1.2$. For ratios with the 95% confidence interval completely above or below the band, the vertical bars are colored red to indicate non-equivalence of the QR and OLS effect sizes and thus heterogeneity of $\widehat{\beta}_\tau$ and the linear predictive value of the PGS across the phenotypic range.

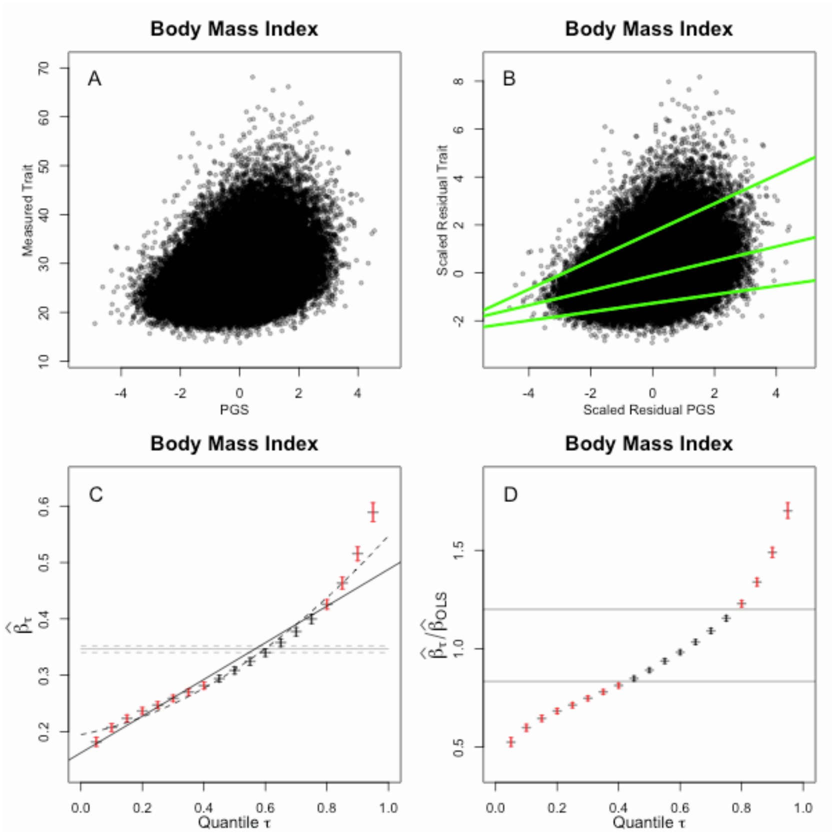

Figure S5. Body mass index

Panels A and B show scatter plots of the trait (body mass index; BMI) versus polygenic score for participants in the UK Biobank. In panel A and trait and PGS as reported in the data set are plotted. In panel B, residualized and scaled trait and PGS values are used, after projection of age, age^2, sex, age*sex, and ten genetic principal components and rescaling the residuals to mean zero variance one. The green lines show linear models fit to the data using QR at the 95th, 50th, and 5th quantiles of the residual trait distribution. QR is used to estimate quantile specific linear effect sizes $\widehat{\beta}_\tau$ at 19 quantiles τ of the residual trait distribution as in panel B. In panel C, $\widehat{\beta}_\tau$ is plotted against τ, with vertical bars representing 95% confidence intervals for $\widehat{\beta}_\tau$. The gray horizontal line with dashed lines above and below represent $\widehat{\beta}_{\mathrm{OLS}}$ and its 95% confidence interval for a linear model fit to residual data as in panel B. In panel D, $\widehat{\beta}_\tau/\widehat{\beta}_{\mathrm{OLS}}$ versus τ is plotted with vertical bars representing 95% confidence intervals for the ratio. Homogeneous effect sizes across the phenotypic distribution correspond to ratios $\widehat{\beta}_\tau/\widehat{\beta}_{\mathrm{OLS}}$ equal to one for each τ. The gray horizontal lines in panel D bound an equivalence band where the ratio is between $(1/\gamma, \gamma)$ for $\gamma = 1.2$. For ratios with the 95% confidence interval completely above or below the band, the vertical bars are colored red to indicate non-equivalence of the QR and OLS effect sizes and thus heterogeneity of $\widehat{\beta}_\tau$ and the linear predictive value of the PGS across the phenotypic range.

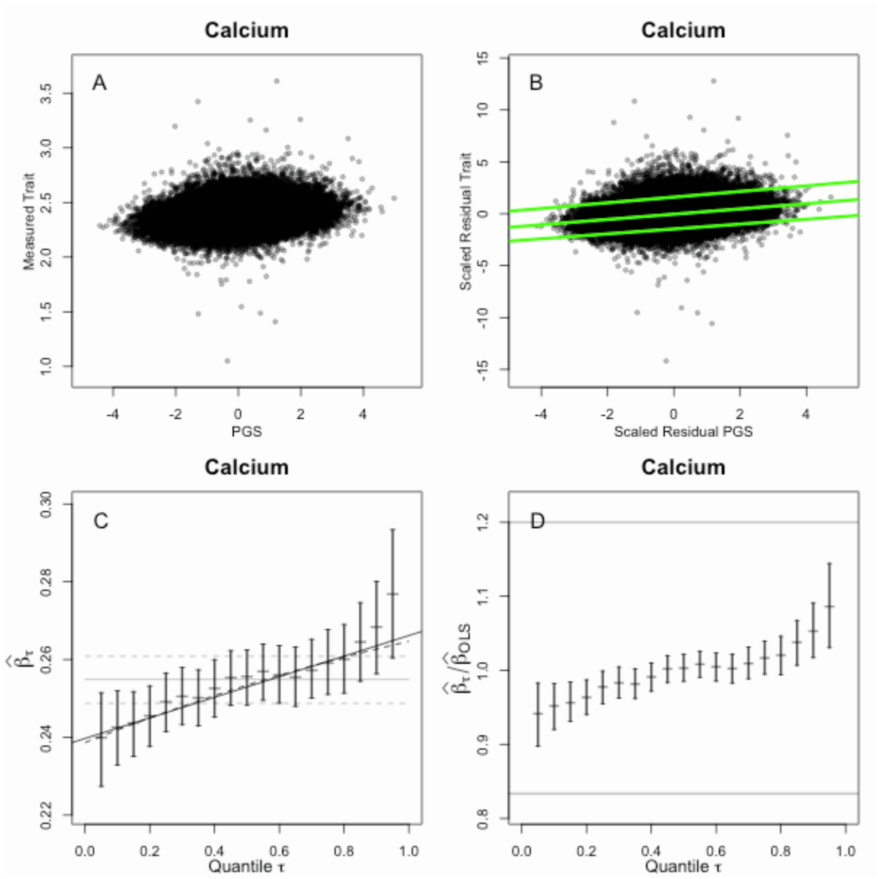

Figure S6. Calcium

Panels A and B show scatter plots of the trait (calcium) versus polygenic score for participants in the UK Biobank. In panel A and trait and PGS as reported in the data set are plotted. In panel B, residualized and scaled trait and PGS values are used, after projection of age, age^2, sex, age*sex, and ten genetic principal components and rescaling the residuals to mean zero variance one. The green lines show linear models fit to the data using QR at the 95th, 50th, and 5th quantiles of the residual trait distribution. QR is used to estimate quantile specific linear effect sizes $\widehat{\beta}_\tau$ at 19 quantiles τ of the residual trait distribution as in panel B. In panel C, $\widehat{\beta}_\tau$ is plotted against τ, with vertical bars representing 95% confidence intervals for $\widehat{\beta}_\tau$. The gray horizontal line with dashed lines above and below represent $\widehat{\beta}_{\mathrm{OLS}}$ and its 95% confidence interval for a linear model fit to residual data as in panel B. In panel D, $\widehat{\beta}_\tau/\widehat{\beta}_{\mathrm{OLS}}$ versus τ is plotted with vertical bars representing 95% confidence intervals for the ratio. Homogeneous effect sizes across the phenotypic distribution correspond to ratios $\widehat{\beta}_\tau/\widehat{\beta}_{\mathrm{OLS}}$ equal to one for each τ. The gray horizontal lines in panel D bound an equivalence band where the ratio is between $(1/\gamma, \gamma)$ for $\gamma = 1.2$. For ratios with the 95% confidence interval completely above or below the band, the vertical bars are colored red to indicate non-equivalence of the QR and OLS effect sizes and thus heterogeneity of $\widehat{\beta}_\tau$ and the linear predictive value of the PGS across the phenotypic range.

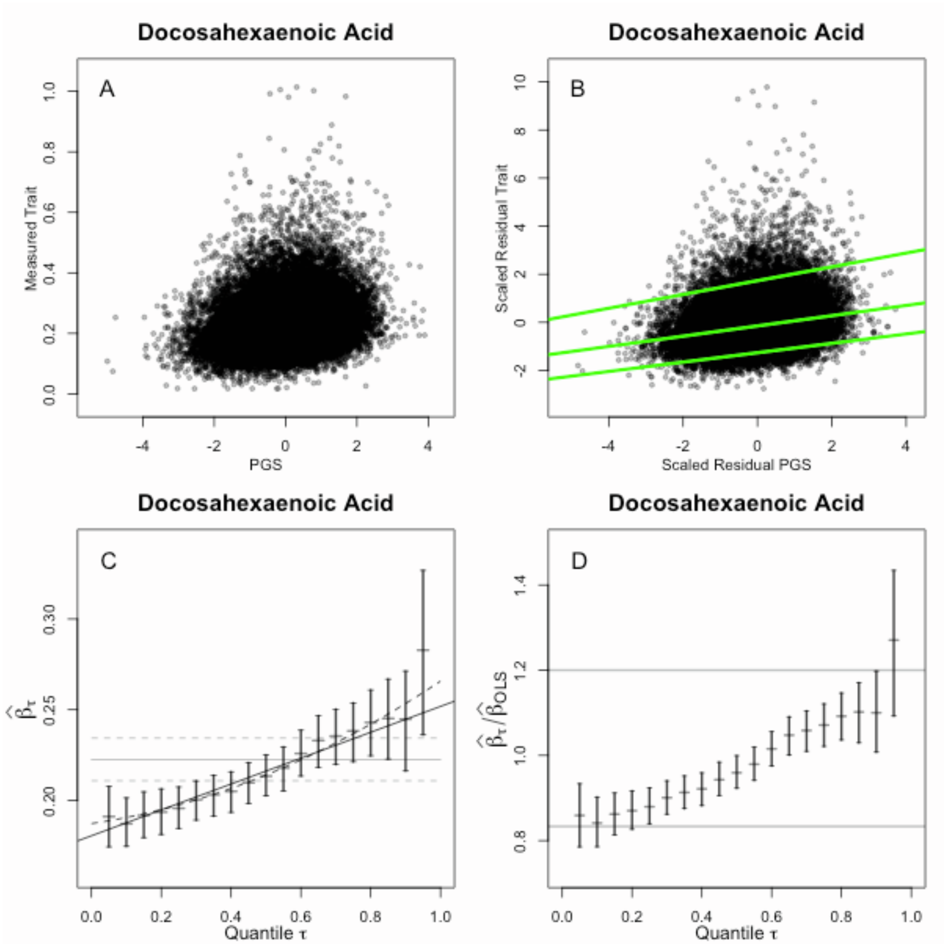

Figure S7. Docasahexaenoic Acid

Panels A and B show scatter plots of the trait (docasahexaenoic Acid) versus polygenic score for participants in the UK Biobank. In panel A and trait and PGS as reported in the data set are plotted. In panel B, residualized and scaled trait and PGS values are used, after projection of age, age^2, sex, age*sex, and ten genetic principal components and rescaling the residuals to mean zero variance one. The green lines show linear models fit to the data using QR at the 95th, 50th, and 5th quantiles of the residual trait distribution. QR is used to estimate quantile specific linear effect sizes $\widehat{\beta}_\tau$ at 19 quantiles τ of the residual trait distribution as in panel B. In panel C, $\widehat{\beta}_\tau$ is plotted against τ, with vertical bars representing 95% confidence intervals for $\widehat{\beta}_\tau$. The gray horizontal line with dashed lines above and below represent $\widehat{\beta}_{\mathrm{OLS}}$ and its 95% confidence interval for a linear model fit to residual data as in panel B. In panel D, $\widehat{\beta}_\tau/\widehat{\beta}_{\mathrm{OLS}}$ versus τ is plotted with vertical bars representing 95% confidence intervals for the ratio. Homogeneous effect sizes across the phenotypic distribution correspond to ratios $\widehat{\beta}_\tau/\widehat{\beta}_{\mathrm{OLS}}$ equal to one for each τ. The gray horizontal lines in panel D bound an equivalence band where the ratio is between $(1/\gamma, \gamma)$ for $\gamma = 1.2$. For ratios with the 95% confidence interval completely above or below the band, the vertical bars are colored red to indicate non-equivalence of the QR and OLS effect sizes and thus heterogeneity of $\widehat{\beta}_\tau$ and the linear predictive value of the PGS across the phenotypic range.

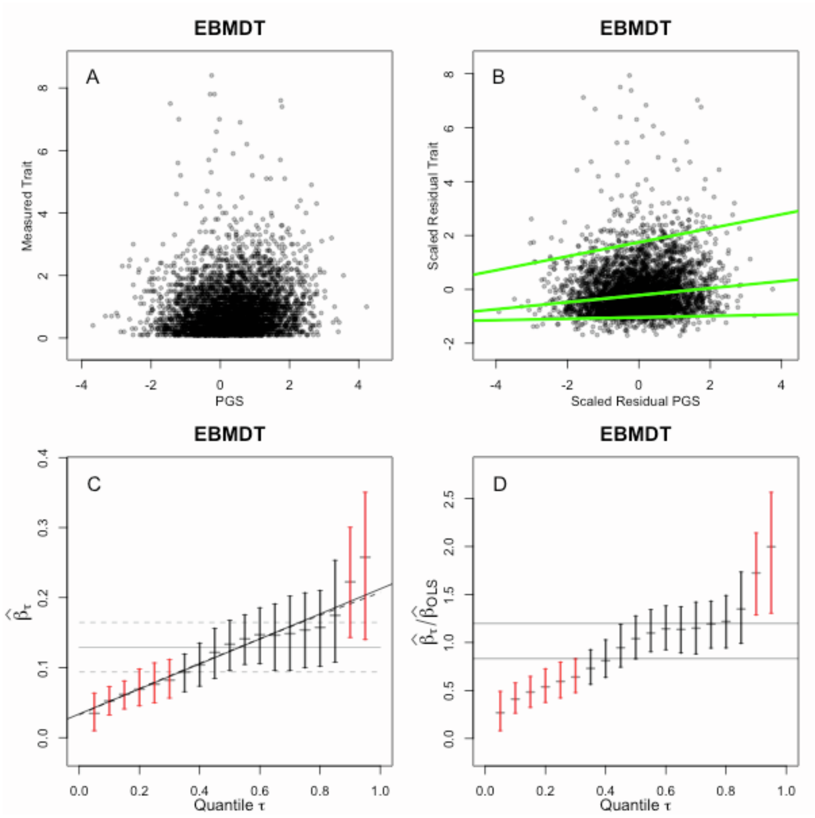

Figure S8. Estimated bone mineral density T score (EBMDT)

Panels A and B show scatter plots of the trait (estimated bone mineral density T score) versus polygenic score for participants in the UK Biobank. In panel A and trait and PGS as reported in the data set are plotted. In panel B, residualized and scaled trait and PGS values are used, after projection of age, age^2, sex, age*sex, and ten genetic principal components and rescaling the residuals to mean zero variance one. The green lines show linear models fit to the data using QR at the 95th, 50th, and 5th quantiles of the residual trait distribution. QR is used to estimate quantile specific linear effect sizes $\widehat{\beta}_{\tau}$ at 19 quantiles τ of the residual trait distribution as in panel B. In panel C, $\widehat{\beta}_{\tau}$ is plotted against τ, with vertical bars representing 95% confidence intervals for $\widehat{\beta}_{\tau}$. The gray horizontal line with dashed lines above and below represent $\widehat{\beta}_{\mathrm{OLS}}$ and its 95% confidence interval for a linear model fit to residual data as in panel B. In panel D, $\widehat{\beta}_{\tau}/\widehat{\beta}_{\mathrm{OLS}}$ versus τ is plotted with vertical bars representing 95% confidence intervals for the ratio. Homogeneous effect sizes across the phenotypic distribution correspond to ratios $\widehat{\beta}_{\tau}/\widehat{\beta}_{\mathrm{OLS}}$ equal to one for each τ. The gray horizontal lines in panel D bound an equivalence band where the ratio is between $(1/\gamma, \gamma)$ for $\gamma = 1.2$. For ratios with the 95% confidence interval completely above or below the band, the vertical bars are colored red to indicate non-equivalence of the QR and OLS effect sizes and thus heterogeneity of $\widehat{\beta}_{\tau}$ and the linear predictive value of the PGS across the phenotypic range.

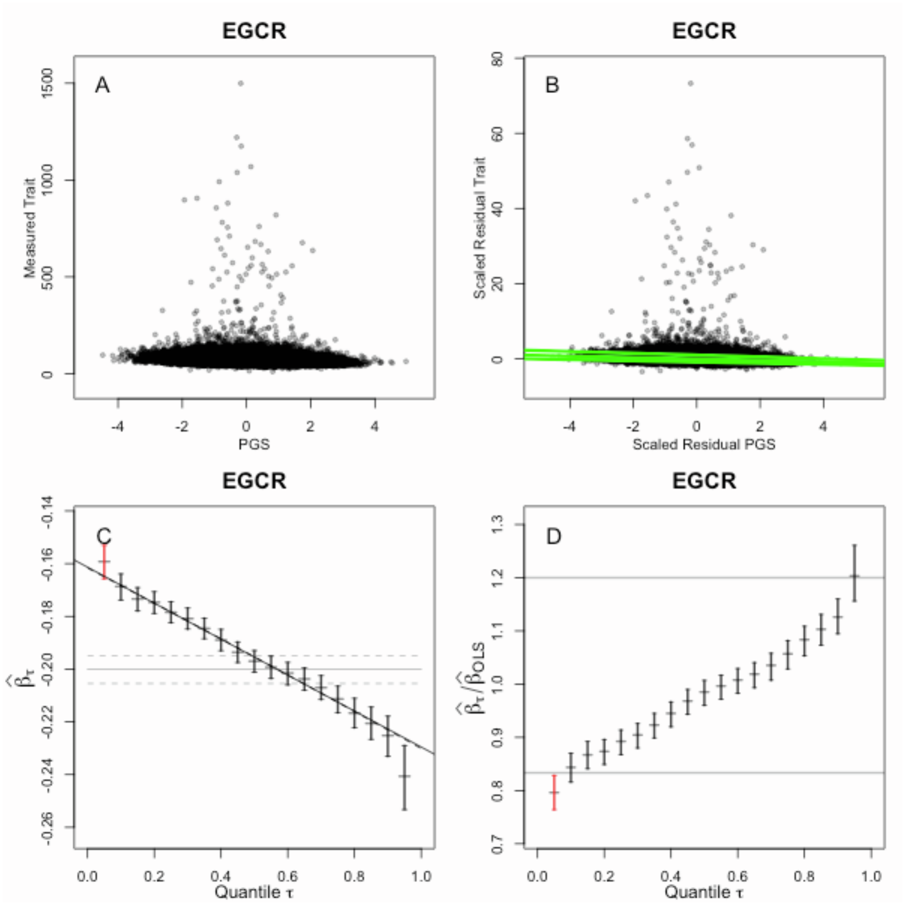

Figure S9. Estimated glomerular filtration rate, creatinin method, EGCR

Panels A and B show scatter plots of the trait (estimated glomerular filtration rate – creatinin method) versus polygenic score for participants in the UK Biobank. In panel A and trait and PGS as reported in the data set are plotted. In panel B, residualized and scaled trait and PGS values are used, after projection of age, age^2, sex, age*sex, and ten genetic principal components and rescaling the residuals to mean zero variance one. The green lines show linear models fit to the data using QR at the 95th, 50th, and 5th quantiles of the residual trait distribution. QR is used to estimate quantile specific linear effect sizes $\widehat{\beta}_\tau$ at 19 quantiles τ of the residual trait distribution as in panel B. In panel C, $\widehat{\beta}_\tau$ is plotted against τ, with vertical bars representing 95% confidence intervals for $\widehat{\beta}_\tau$. The gray horizontal line with dashed lines above and below represent $\widehat{\beta}_{\mathrm{OLS}}$ and its 95% confidence interval for a linear model fit to residual data as in panel B. In panel D, $\widehat{\beta}_\tau/\widehat{\beta}_{\mathrm{OLS}}$ versus τ is plotted with vertical bars representing 95% confidence intervals for the ratio. Homogeneous effect sizes across the phenotypic distribution correspond to ratios $\widehat{\beta}_\tau/\widehat{\beta}_{\mathrm{OLS}}$ equal to one for each τ. The gray horizontal lines in panel D bound an equivalence band where the ratio is between $(1/\gamma, \gamma)$ for $\gamma = 1.2$. For ratios with the 95% confidence interval completely above or below the band, the vertical bars are colored red to indicate non-equivalence of the QR and OLS effect sizes and thus heterogeneity of $\widehat{\beta}_\tau$ and the linear predictive value of the PGS across the phenotypic range.

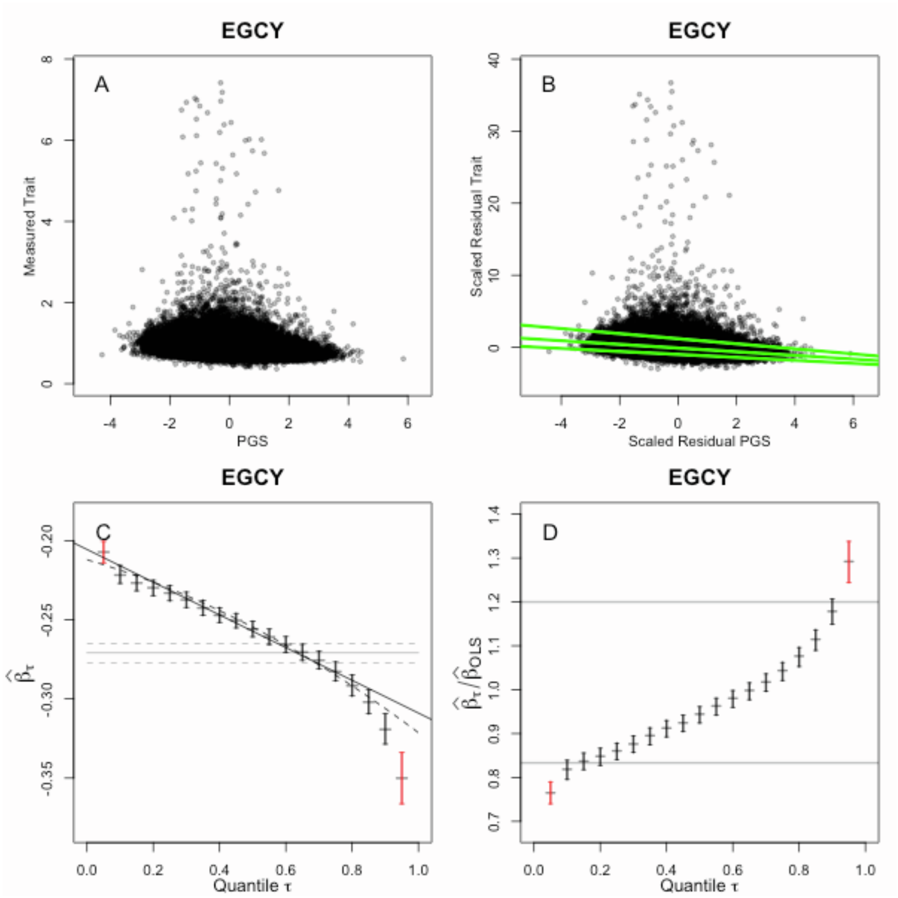

Figure S10. Estimated glomerular filtration rate cystatin method, EGCY

Panels A and B show scatter plots of the trait (estimated glomerular filtration rate – cystatin method) versus polygenic score for participants in the UK Biobank. In panel A and trait and PGS as reported in the data set are plotted. In panel B, residualized and scaled trait and PGS values are used, after projection of age, age^2, sex, age*sex, and ten genetic principal components and rescaling the residuals to mean zero variance one. The green lines show linear models fit to the data using QR at the 95th, 50th, and 5th quantiles of the residual trait distribution. QR is used to estimate quantile specific linear effect sizes $\widehat{\beta}_\tau$ at 19 quantiles τ of the residual trait distribution as in panel B. In panel C, $\widehat{\beta}_\tau$ is plotted against τ, with vertical bars representing 95% confidence intervals for $\widehat{\beta}_\tau$. The gray horizontal line with dashed lines above and below represent $\widehat{\beta}_{\mathrm{OLS}}$ and its 95% confidence interval for a linear model fit to residual data as in panel B. In panel D, $\widehat{\beta}_\tau/\widehat{\beta}_{\mathrm{OLS}}$ versus τ is plotted with vertical bars representing 95% confidence intervals for the ratio. Homogeneous effect sizes across the phenotypic distribution correspond to ratios $\widehat{\beta}_\tau/\widehat{\beta}_{\mathrm{OLS}}$ equal to one for each τ. The gray horizontal lines in panel D bound an equivalence band where the ratio is between $(1/\gamma, \gamma)$ for $\gamma = 1.2$. For ratios with the 95% confidence interval completely above or below the band, the vertical bars are colored red to indicate non-equivalence of the QR and OLS effect sizes and thus heterogeneity of $\widehat{\beta}_\tau$ and the linear predictive value of the PGS across the phenotypic range.

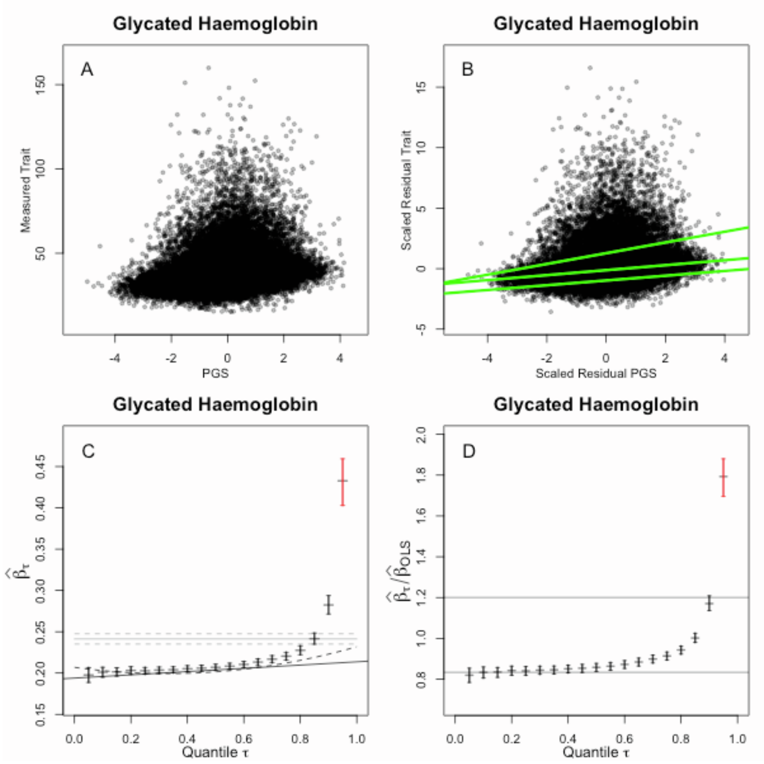

Figure S11. Glycated haemoglobin, HbA1c

Panels A and B show scatter plots of the trait (glycated haemoglobin, HbA1c) versus polygenic score for participants in the UK Biobank. In panel A and trait and PGS as reported in the data set are plotted. In panel B, residualized and scaled trait and PGS values are used, after projection of age, age^2, sex, age*sex, and ten genetic principal components and rescaling the residuals to mean zero variance one. The green lines show linear models fit to the data using QR at the 95th, 50th, and 5th quantiles of the residual trait distribution. QR is used to estimate quantile specific linear effect sizes $\widehat{\beta}_\tau$ at 19 quantiles τ of the residual trait distribution as in panel B. In panel C, $\widehat{\beta}_\tau$ is plotted against τ, with vertical bars representing 95% confidence intervals for $\widehat{\beta}_\tau$. The gray horizontal line with dashed lines above and below represent $\widehat{\beta}_{\mathrm{OLS}}$ and its 95% confidence interval for a linear model fit to residual data as in panel B. In panel D, $\widehat{\beta}_\tau/\widehat{\beta}_{\mathrm{OLS}}$ versus τ is plotted with vertical bars representing 95% confidence intervals for the ratio. Homogeneous effect sizes across the phenotypic distribution correspond to ratios $\widehat{\beta}_\tau/\widehat{\beta}_{\mathrm{OLS}}$ equal to one for each τ. The gray horizontal lines in panel D bound an equivalence band where the ratio is between $(1/\gamma, \gamma)$ for $\gamma = 1.2$. For ratios with the 95% confidence interval completely above or below the band, the vertical bars are colored red to indicate non-equivalence of the QR and OLS effect sizes and thus heterogeneity of $\widehat{\beta}_\tau$ and the linear predictive value of the PGS across the phenotypic range.

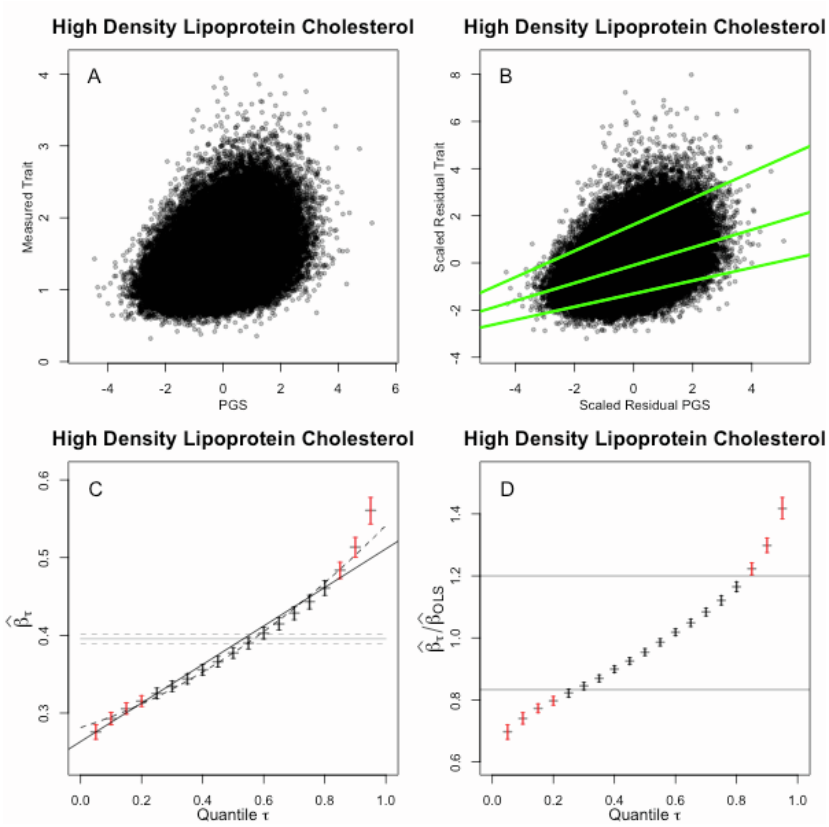

Figure S12. High density lipoprotein cholesterol, HDLc

Panels A and B show scatter plots of the trait (high density lipoprotein cholesterol, HDLc) versus polygenic score for participants in the UK Biobank. In panel A and trait and PGS as reported in the data set are plotted. In panel B, residualized and scaled trait and PGS values are used, after projection of age, age^2, sex, age*sex, and ten genetic principal components and rescaling the residuals to mean zero variance one. The green lines show linear models fit to the data using QR at the 95th, 50th, and 5th quantiles of the residual trait distribution. QR is used to estimate quantile specific linear effect sizes $\widehat{\beta}_\tau$ at 19 quantiles τ of the residual trait distribution as in panel B. In panel C, $\widehat{\beta}_\tau$ is plotted against τ, with vertical bars representing 95% confidence intervals for $\widehat{\beta}_\tau$. The gray horizontal line with dashed lines above and below represent $\widehat{\beta}_{\mathrm{OLS}}$ and its 95% confidence interval for a linear model fit to residual data as in panel B. In panel D, $\widehat{\beta}_\tau/\widehat{\beta}_{\mathrm{OLS}}$ versus τ is plotted with vertical bars representing 95% confidence intervals for the ratio. Homogeneous effect sizes across the phenotypic distribution correspond to ratios $\widehat{\beta}_\tau/\widehat{\beta}_{\mathrm{OLS}}$ equal to one for each τ. The gray horizontal lines in panel D bound an equivalence band where the ratio is between $(1/\gamma, \gamma)$ for $\gamma = 1.2$. For ratios with the 95% confidence interval completely above or below the band, the vertical bars are colored red to indicate non-equivalence of the QR and OLS effect sizes and thus heterogeneity of $\widehat{\beta}_\tau$ and the linear predictive value of the PGS across the phenotypic range.

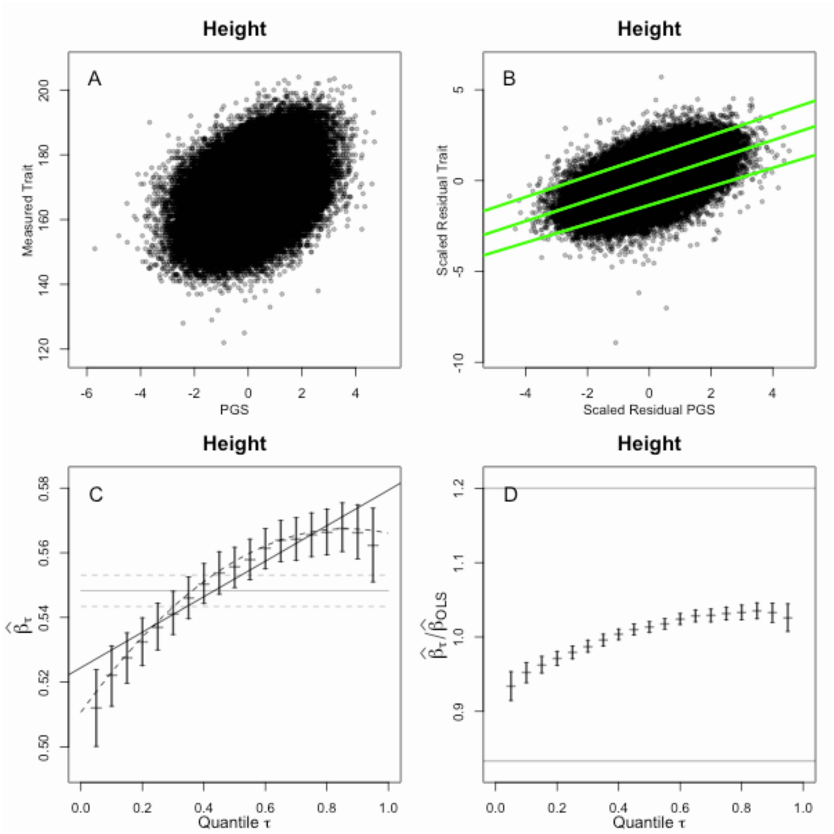

Figure S13. Height

Panels A and B show scatter plots of the trait (height) versus polygenic score for participants in the UK Biobank. In panel A and trait and PGS as reported in the data set are plotted. In panel B, residualized and scaled trait and PGS values are used, after projection of age, age^2, sex, age*sex, and ten genetic principal components and rescaling the residuals to mean zero variance one. The green lines show linear models fit to the data using QR at the 95th, 50th, and 5th quantiles of the residual trait distribution. QR is used to estimate quantile specific linear effect sizes $\widehat{\beta}_\tau$ at 19 quantiles τ of the residual trait distribution as in panel B. In panel C, $\widehat{\beta}_\tau$ is plotted against τ, with vertical bars representing 95% confidence intervals for $\widehat{\beta}_\tau$. The gray horizontal line with dashed lines above and below represent $\widehat{\beta}_{\mathrm{OLS}}$ and its 95% confidence interval for a linear model fit to residual data as in panel B. In panel D, $\widehat{\beta}_\tau/\widehat{\beta}_{\mathrm{OLS}}$ versus τ is plotted with vertical bars representing 95% confidence intervals for the ratio. Homogeneous effect sizes across the phenotypic distribution correspond to ratios $\widehat{\beta}_\tau/\widehat{\beta}_{\mathrm{OLS}}$ equal to one for each τ. The gray horizontal lines in panel D bound an equivalence band where the ratio is between $(1/\gamma, \gamma)$ for $\gamma = 1.2$. For ratios with the 95% confidence interval completely above or below the band, the vertical bars are colored red to indicate non-equivalence of the QR and OLS effect sizes and thus heterogeneity of $\widehat{\beta}_\tau$ and the linear predictive value of the PGS across the phenotypic range.

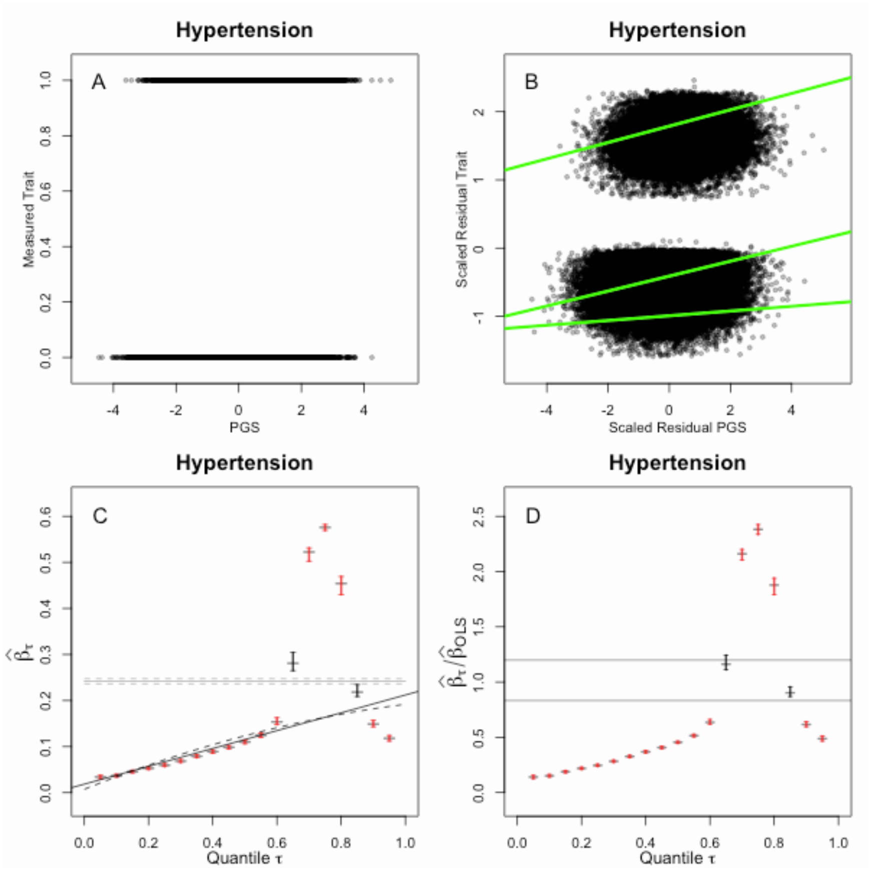

Figure S14. Hypertension

Panels A and B show scatter plots of the trait (hypertension) versus polygenic score for participants in the UK Biobank. In panel A and trait and PGS as reported in the data set are plotted. In panel B, residualized and scaled trait and PGS values are used, after projection of age, age^2, sex, age*sex, and ten genetic principal components and rescaling the residuals to mean zero variance one. The green lines show linear models fit to the data using QR at the 95th, 50th, and 5th quantiles of the residual trait distribution. QR is used to estimate quantile specific linear effect sizes $\widehat{\beta}_\tau$ at 19 quantiles τ of the residual trait distribution as in panel B. In panel C, $\widehat{\beta}_\tau$ is plotted against τ, with vertical bars representing 95% confidence intervals for $\widehat{\beta}_\tau$. The gray horizontal line with dashed lines above and below represent $\widehat{\beta}_{\text{OLS}}$ and its 95% confidence interval for a linear model fit to residual data as in panel B. In panel D, $\widehat{\beta}_\tau/\widehat{\beta}_{\text{OLS}}$ versus τ is plotted with vertical bars representing 95% confidence intervals for the ratio. Homogeneous effect sizes across the phenotypic distribution correspond to ratios $\widehat{\beta}_\tau/\widehat{\beta}_{\text{OLS}}$ equal to one for each τ. The gray horizontal lines in panel D bound an equivalence band where the ratio is between $(1/\gamma, \gamma)$ for $\gamma = 1.2$. For ratios with the 95% confidence interval completely above or below the band, the vertical bars are colored red to indicate non-equivalence of the QR and OLS effect sizes and thus heterogeneity of $\widehat{\beta}_\tau$ and the linear predictive value of the PGS across the phenotypic range.

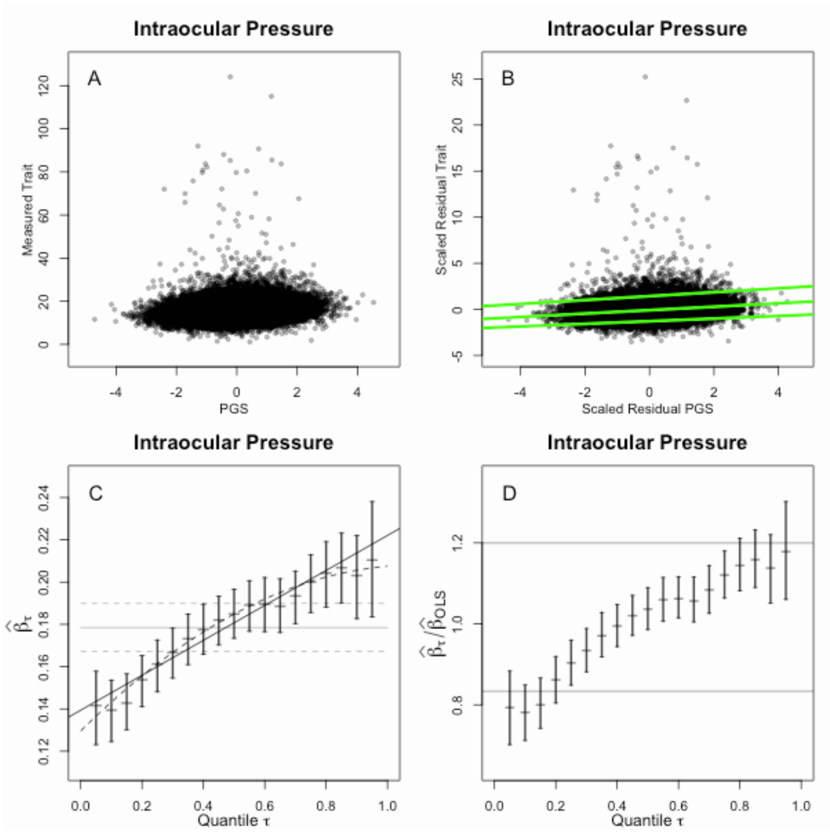

Figure S15. Intraocular Pressure

Panels A and B show scatter plots of the trait (intraocular pressure) versus polygenic score for participants in the UK Biobank. In panel A and trait and PGS as reported in the data set are plotted. In panel B, residualized and scaled trait and PGS values are used, after projection of age, age^2, sex, age*sex, and ten genetic principal components and rescaling the residuals to mean zero variance one. The green lines show linear models fit to the data using QR at the 95th, 50th, and 5th quantiles of the residual trait distribution. QR is used to estimate quantile specific linear effect sizes $\widehat{\beta}_\tau$ at 19 quantiles τ of the residual trait distribution as in panel B. In panel C, $\widehat{\beta}_\tau$ is plotted against τ, with vertical bars representing 95% confidence intervals for $\widehat{\beta}_\tau$. The gray horizontal line with dashed lines above and below represent $\widehat{\beta}_{\text{OLS}}$ and its 95% confidence interval for a linear model fit to residual data as in panel B. In panel D, $\widehat{\beta}_\tau/\widehat{\beta}_{\text{OLS}}$ versus τ is plotted with vertical bars representing 95% confidence intervals for the ratio. Homogeneous effect sizes across the phenotypic distribution correspond to ratios $\widehat{\beta}_\tau/\widehat{\beta}_{\text{OLS}}$ equal to one for each τ. The gray horizontal lines in panel D bound an equivalence band where the ratio is between $(1/\gamma, \gamma)$ for $\gamma = 1.2$. For ratios with the 95% confidence interval completely above or below the band, the vertical bars are colored red to indicate non-equivalence of the QR and OLS effect sizes and thus heterogeneity of $\widehat{\beta}_\tau$ and the linear predictive value of the PGS across the phenotypic range.

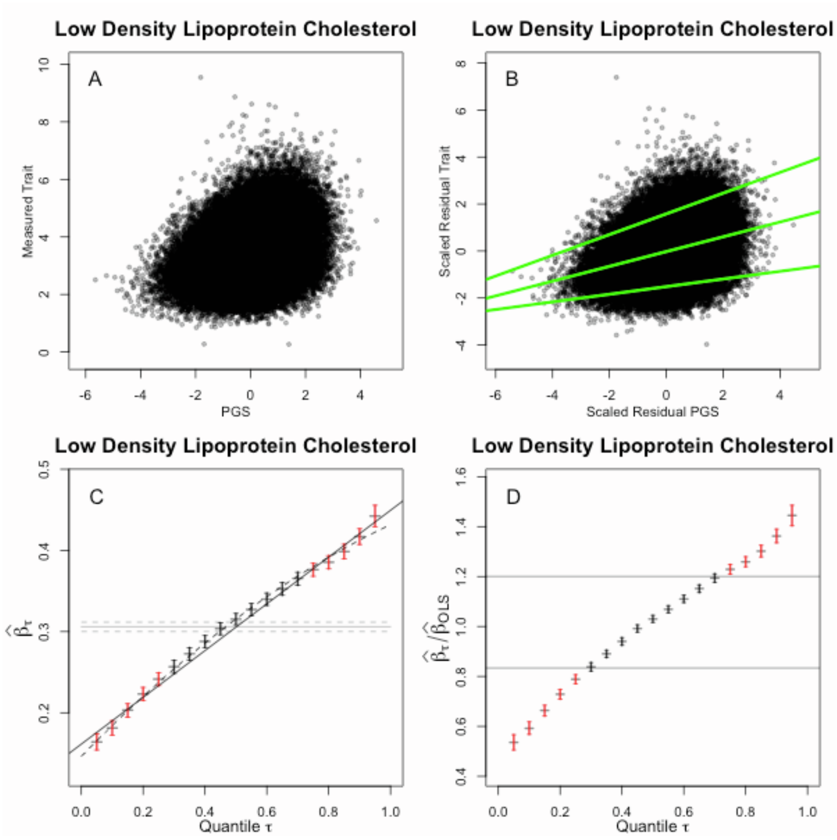

Figure S16. Low density lipoprotein cholesterol, LDLc

Panels A and B show scatter plots of the trait (low density lipoprotein cholesterol, LDLc) versus polygenic score for participants in the UK Biobank. In panel A and trait and PGS as reported in the data set are plotted. In panel B, residualized and scaled trait and PGS values are used, after projection of age, age^2, sex, age*sex, and ten genetic principal components and rescaling the residuals to mean zero variance one. The green lines show linear models fit to the data using QR at the 95th, 50th, and 5th quantiles of the residual trait distribution. QR is used to estimate quantile specific linear effect sizes $\widehat{\beta}_\tau$ at 19 quantiles τ of the residual trait distribution as in panel B. In panel C, $\widehat{\beta}_\tau$ is plotted against τ, with vertical bars representing 95% confidence intervals for $\widehat{\beta}_\tau$. The gray horizontal line with dashed lines above and below represent $\widehat{\beta}_{\mathrm{OLS}}$ and its 95% confidence interval for a linear model fit to residual data as in panel B. In panel D, $\widehat{\beta}_\tau/\widehat{\beta}_{\mathrm{OLS}}$ versus τ is plotted with vertical bars representing 95% confidence intervals for the ratio. Homogeneous effect sizes across the phenotypic distribution correspond to ratios $\widehat{\beta}_\tau/\widehat{\beta}_{\mathrm{OLS}}$ equal to one for each τ. The gray horizontal lines in panel D bound an equivalence band where the ratio is between $(1/\gamma, \gamma)$ for $\gamma = 1.2$. For ratios with the 95% confidence interval completely above or below the band, the vertical bars are colored red to indicate non-equivalence of the QR and OLS effect sizes and thus heterogeneity of $\widehat{\beta}_\tau$ and the linear predictive value of the PGS across the phenotypic range.

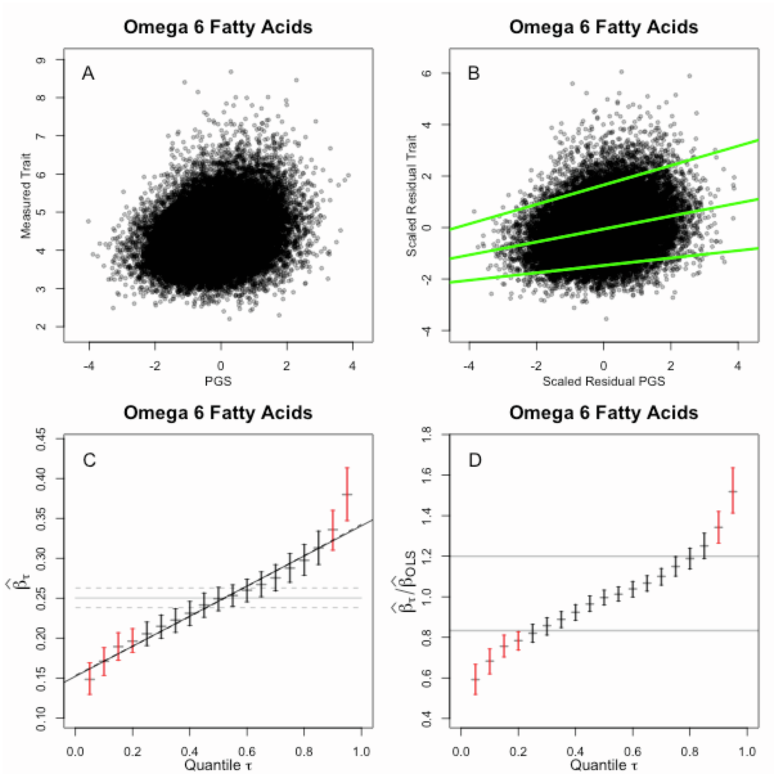

Figure S17. Omega 6 fatty acids

Panels A and B show scatter plots of the trait (omega 6 fatty acids) versus polygenic score for participants in the UK Biobank. In panel A and trait and PGS as reported in the data set are plotted. In panel B, residualized and scaled trait and PGS values are used, after projection of age, age^2, sex, age*sex, and ten genetic principal components and rescaling the residuals to mean zero variance one. The green lines show linear models fit to the data using QR at the 95th, 50th, and 5th quantiles of the residual trait distribution. QR is used to estimate quantile specific linear effect sizes $\widehat{\beta}_\tau$ at 19 quantiles τ of the residual trait distribution as in panel B. In panel C, $\widehat{\beta}_\tau$ is plotted against τ, with vertical bars representing 95% confidence intervals for $\widehat{\beta}_\tau$. The gray horizontal line with dashed lines above and below represent $\widehat{\beta}_{\mathrm{OLS}}$ and its 95% confidence interval for a linear model fit to residual data as in panel B. In panel D, $\widehat{\beta}_\tau/\widehat{\beta}_{\mathrm{OLS}}$ versus τ is plotted with vertical bars representing 95% confidence intervals for the ratio. Homogeneous effect sizes across the phenotypic distribution correspond to ratios $\widehat{\beta}_\tau/\widehat{\beta}_{\mathrm{OLS}}$ equal to one for each τ. The gray horizontal lines in panel D bound an equivalence band where the ratio is between $(1/\gamma, \gamma)$ for $\gamma = 1.2$. For ratios with the 95% confidence interval completely above or below the band, the vertical bars are colored red to indicate non-equivalence of the QR and OLS effect sizes and thus heterogeneity of $\widehat{\beta}_\tau$ and the linear predictive value of the PGS across the phenotypic range.

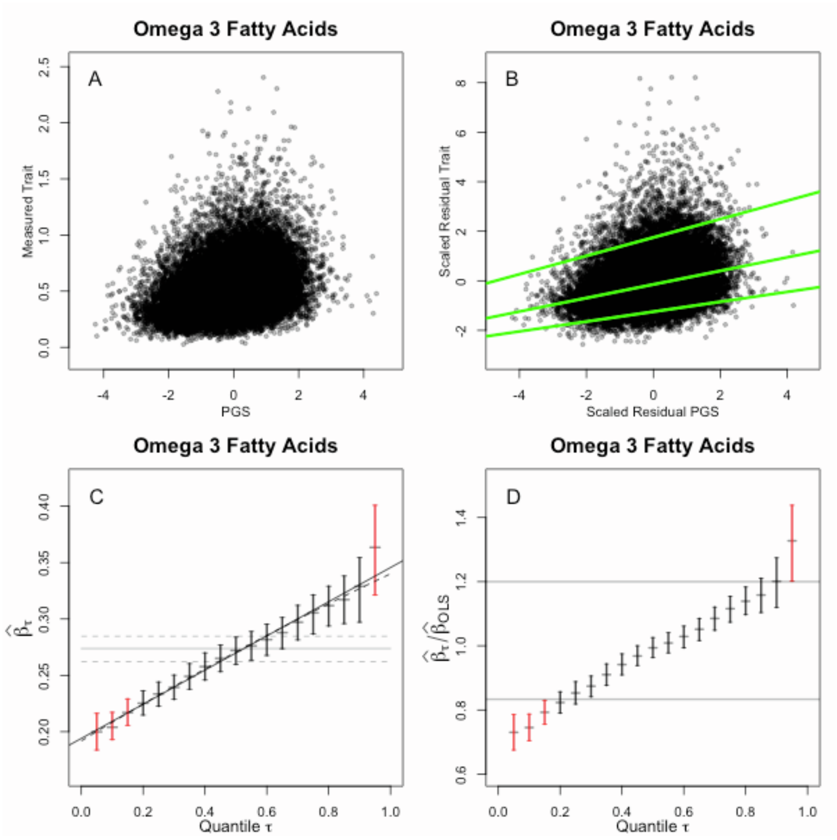

Figure S18. Omega 3 fatty acids

Panels A and B show scatter plots of the trait (omega 3 fatty acids) versus polygenic score for participants in the UK Biobank. In panel A and trait and PGS as reported in the data set are plotted. In panel B, residualized and scaled trait and PGS values are used, after projection of age, age^2, sex, age*sex, and ten genetic principal components and rescaling the residuals to mean zero variance one. The green lines show linear models fit to the data using QR at the 95th, 50th, and 5th quantiles of the residual trait distribution. QR is used to estimate quantile specific linear effect sizes $\widehat{\beta}_\tau$ at 19 quantiles τ of the residual trait distribution as in panel B. In panel C, $\widehat{\beta}_\tau$ is plotted against τ, with vertical bars representing 95% confidence intervals for $\widehat{\beta}_\tau$. The gray horizontal line with dashed lines above and below represent $\widehat{\beta}_{\mathrm{OLS}}$ and its 95% confidence interval for a linear model fit to residual data as in panel B. In panel D, $\widehat{\beta}_\tau/\widehat{\beta}_{\mathrm{OLS}}$ versus τ is plotted with vertical bars representing 95% confidence intervals for the ratio. Homogeneous effect sizes across the phenotypic distribution correspond to ratios $\widehat{\beta}_\tau/\widehat{\beta}_{\mathrm{OLS}}$ equal to one for each τ. The gray horizontal lines in panel D bound an equivalence band where the ratio is between $(1/\gamma, \gamma)$ for $\gamma = 1.2$. For ratios with the 95% confidence interval completely above or below the band, the vertical bars are colored red to indicate non-equivalence of the QR and OLS effect sizes and thus heterogeneity of $\widehat{\beta}_\tau$ and the linear predictive value of the PGS across the phenotypic range.

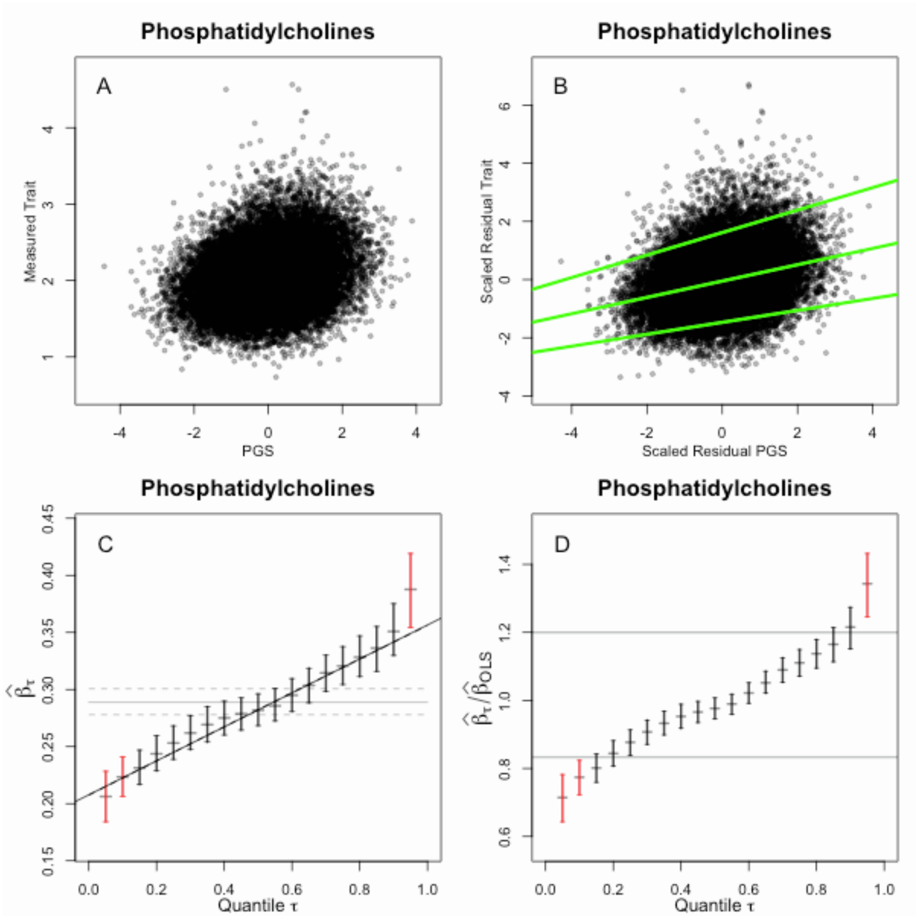

Figure S19. Phosphatidylcholines

Panels A and B show scatter plots of the trait (phosphatidylcholines) versus polygenic score for participants in the UK Biobank. In panel A and trait and PGS as reported in the data set are plotted. In panel B, residualized and scaled trait and PGS values are used, after projection of age, age^2, sex, age*sex, and ten genetic principal components and rescaling the residuals to mean zero variance one. The green lines show linear models fit to the data using QR at the 95th, 50th, and 5th quantiles of the residual trait distribution. QR is used to estimate quantile specific linear effect sizes $\widehat{\beta}_\tau$ at 19 quantiles τ of the residual trait distribution as in panel B. In panel C, $\widehat{\beta}_\tau$ is plotted against τ, with vertical bars representing 95% confidence intervals for $\widehat{\beta}_\tau$. The gray horizontal line with dashed lines above and below represent $\widehat{\beta}_{\mathrm{OLS}}$ and its 95% confidence interval for a linear model fit to residual data as in panel B. In panel D, $\widehat{\beta}_\tau/\widehat{\beta}_{\mathrm{OLS}}$ versus τ is plotted with vertical bars representing 95% confidence intervals for the ratio. Homogeneous effect sizes across the phenotypic distribution correspond to ratios $\widehat{\beta}_\tau/\widehat{\beta}_{\mathrm{OLS}}$ equal to one for each τ. The gray horizontal lines in panel D bound an equivalence band where the ratio is between $(1/\gamma, \gamma)$ for $\gamma = 1.2$. For ratios with the 95% confidence interval completely above or below the band, the vertical bars are colored red to indicate non-equivalence of the QR and OLS effect sizes and thus heterogeneity of $\widehat{\beta}_\tau$ and the linear predictive value of the PGS across the phenotypic range.

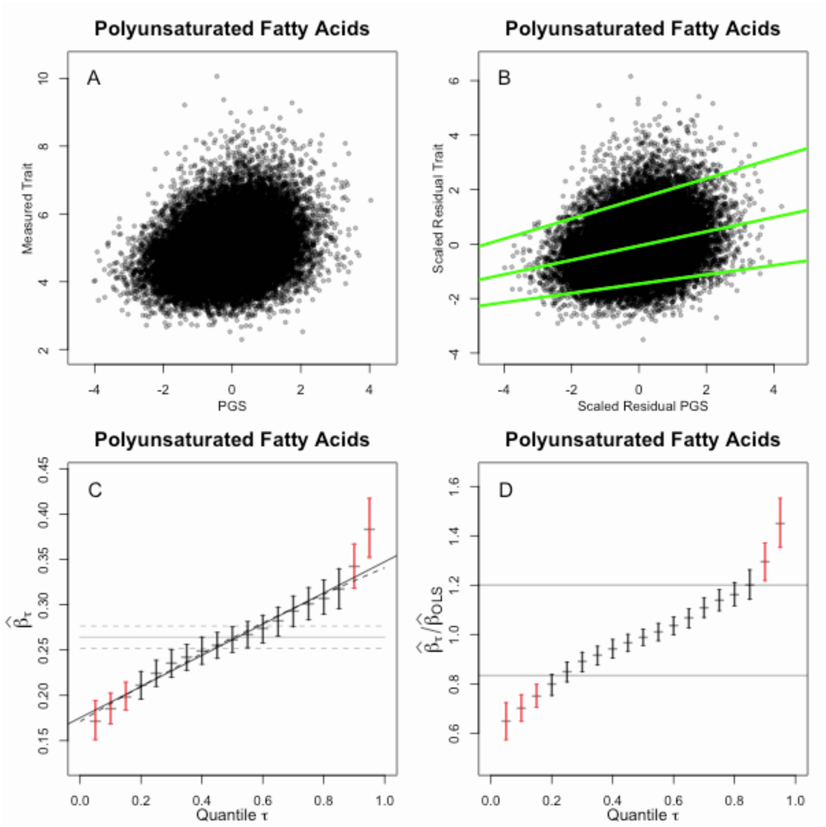

Figure S20. Polyunsaturated fatty acids

Panels A and B show scatter plots of the trait (polyunsaturated fatty acids) versus polygenic score for participants in the UK Biobank. In panel A and trait and PGS as reported in the data set are plotted. In panel B, residualized and scaled trait and PGS values are used, after projection of age, age^2, sex, age*sex, and ten genetic principal components and rescaling the residuals to mean zero variance one. The green lines show linear models fit to the data using QR at the 95th, 50th, and 5th quantiles of the residual trait distribution. QR is used to estimate quantile specific linear effect sizes $\widehat{\beta}_\tau$ at 19 quantiles τ of the residual trait distribution as in panel B. In panel C, $\widehat{\beta}_\tau$ is plotted against τ, with vertical bars representing 95% confidence intervals for $\widehat{\beta}_\tau$. The gray horizontal line with dashed lines above and below represent $\widehat{\beta}_{\mathrm{OLS}}$ and its 95% confidence interval for a linear model fit to residual data as in panel B. In panel D, $\widehat{\beta}_\tau/\widehat{\beta}_{\mathrm{OLS}}$ versus τ is plotted with vertical bars representing 95% confidence intervals for the ratio. Homogeneous effect sizes across the phenotypic distribution correspond to ratios $\widehat{\beta}_\tau/\widehat{\beta}_{\mathrm{OLS}}$ equal to one for each τ. The gray horizontal lines in panel D bound an equivalence band where the ratio is between $(1/\gamma, \gamma)$ for $\gamma = 1.2$. For ratios with the 95% confidence interval completely above or below the band, the vertical bars are colored red to indicate non-equivalence of the QR and OLS effect sizes and thus heterogeneity of $\widehat{\beta}_\tau$ and the linear predictive value of the PGS across the phenotypic range.

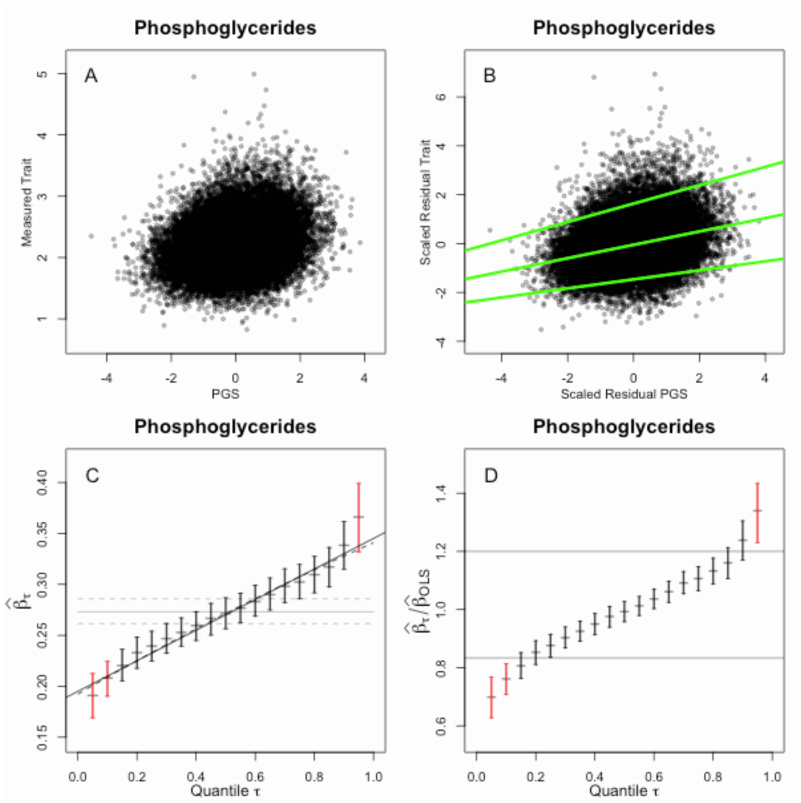

Figure S21. Phosphoglycerides

Panels A and B show scatter plots of the trait (phosphoglycerides) versus polygenic score for participants in the UK Biobank. In panel A and trait and PGS as reported in the data set are plotted. In panel B, residualized and scaled trait and PGS values are used, after projection of age, age^2, sex, age*sex, and ten genetic principal components and rescaling the residuals to mean zero variance one. The green lines show linear models fit to the data using QR at the 95th, 50th, and 5th quantiles of the residual trait distribution. QR is used to estimate quantile specific linear effect sizes $\widehat{\beta}_\tau$ at 19 quantiles τ of the residual trait distribution as in panel B. In panel C, $\widehat{\beta}_\tau$ is plotted against τ, with vertical bars representing 95% confidence intervals for $\widehat{\beta}_\tau$. The gray horizontal line with dashed lines above and below represent $\widehat{\beta}_{\text{OLS}}$ and its 95% confidence interval for a linear model fit to residual data as in panel B. In panel D, $\widehat{\beta}_\tau/\widehat{\beta}_{\text{OLS}}$ versus τ is plotted with vertical bars representing 95% confidence intervals for the ratio. Homogeneous effect sizes across the phenotypic distribution correspond to ratios $\widehat{\beta}_\tau/\widehat{\beta}_{\text{OLS}}$ equal to one for each τ. The gray horizontal lines in panel D bound an equivalence band where the ratio is between $(1/\gamma, \gamma)$ for $\gamma = 1.2$. For ratios with the 95% confidence interval completely above or below the band, the vertical bars are colored red to indicate non-equivalence of the QR and OLS effect sizes and thus heterogeneity of $\widehat{\beta}_\tau$ and the linear predictive value of the PGS across the phenotypic range.

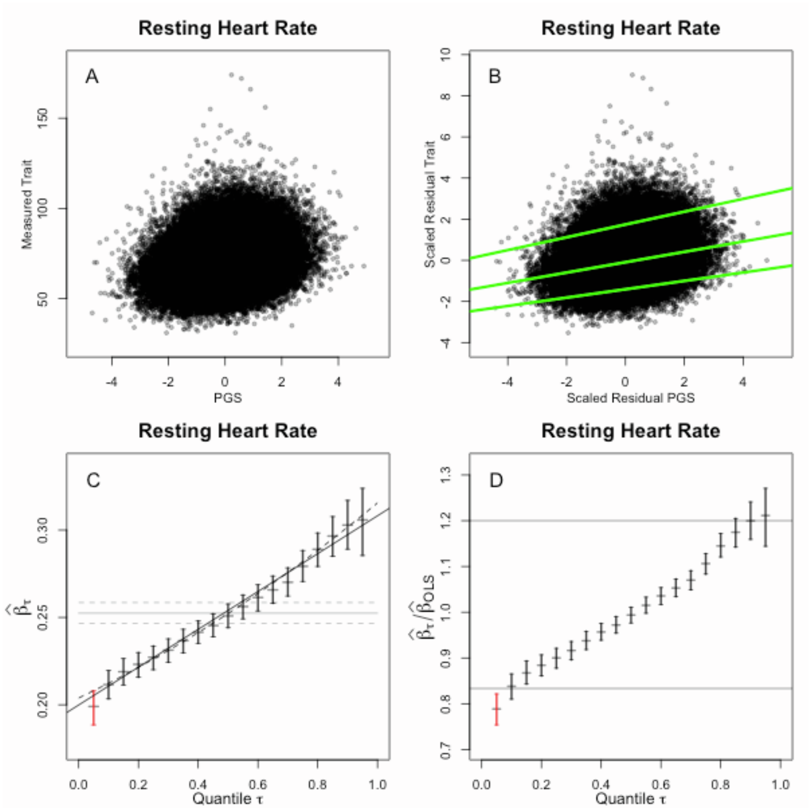

Figure S22. Resting heart rate

Panels A and B show scatter plots of the trait (resting heart rate) versus polygenic score for participants in the UK Biobank. In panel A and trait and PGS as reported in the data set are plotted. In panel B, residualized and scaled trait and PGS values are used, after projection of age, age^2, sex, age*sex, and ten genetic principal components and rescaling the residuals to mean zero variance one. The green lines show linear models fit to the data using QR at the 95th, 50th, and 5th quantiles of the residual trait distribution. QR is used to estimate quantile specific linear effect sizes $\widehat{\beta}_\tau$ at 19 quantiles τ of the residual trait distribution as in panel B. In panel C, $\widehat{\beta}_\tau$ is plotted against τ, with vertical bars representing 95% confidence intervals for $\widehat{\beta}_\tau$. The gray horizontal line with dashed lines above and below represent $\widehat{\beta}_{\mathrm{OLS}}$ and its 95% confidence interval for a linear model fit to residual data as in panel B. In panel D, $\widehat{\beta}_\tau/\widehat{\beta}_{\mathrm{OLS}}$ versus τ is plotted with vertical bars representing 95% confidence intervals for the ratio. Homogeneous effect sizes across the phenotypic distribution correspond to ratios $\widehat{\beta}_\tau/\widehat{\beta}_{\mathrm{OLS}}$ equal to one for each τ. The gray horizontal lines in panel D bound an equivalence band where the ratio is between $(1/\gamma, \gamma)$ for $\gamma = 1.2$. For ratios with the 95% confidence interval completely above or below the band, the vertical bars are colored red to indicate non-equivalence of the QR and OLS effect sizes and thus heterogeneity of $\widehat{\beta}_\tau$ and the linear predictive value of the PGS across the phenotypic range.

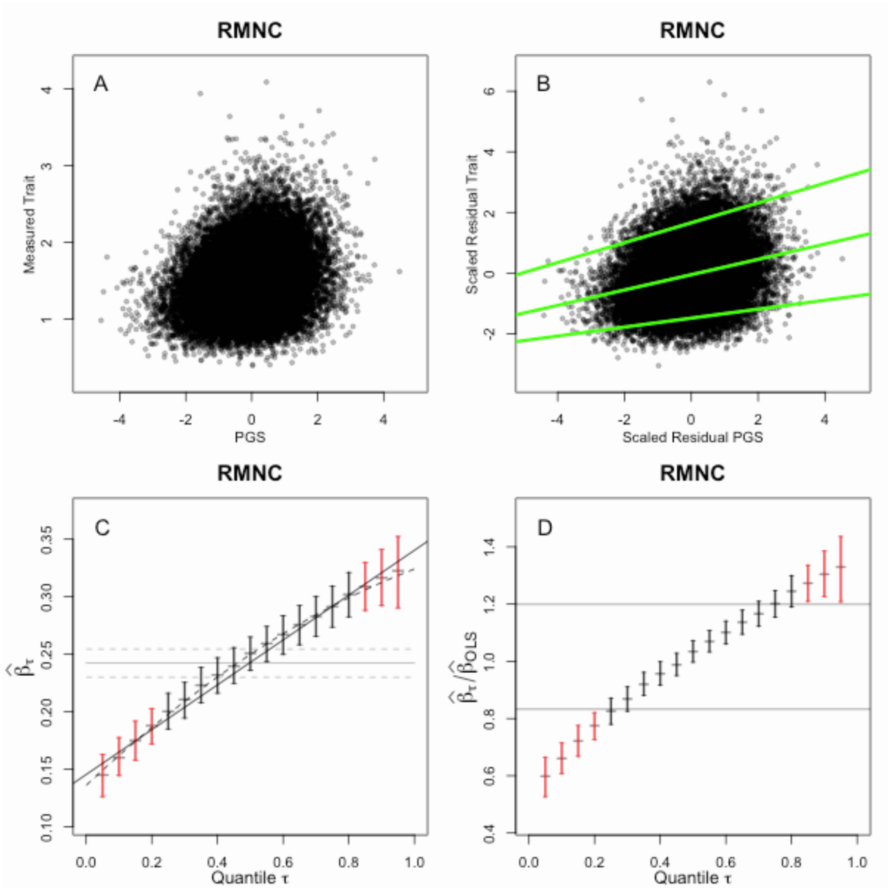

Figure S23. Remnant cholesterol, RMNC

Panels A and B show scatter plots of the trait (remnant cholesterol) versus polygenic score for participants in the UK Biobank. In panel A and trait and PGS as reported in the data set are plotted. In panel B, residualized and scaled trait and PGS values are used, after projection of age, age^2, sex, age*sex, and ten genetic principal components and rescaling the residuals to mean zero variance one. The green lines show linear models fit to the data using QR at the 95th, 50th, and 5th quantiles of the residual trait distribution. QR is used to estimate quantile specific linear effect sizes $\widehat{\beta}_\tau$ at 19 quantiles τ of the residual trait distribution as in panel B. In panel C, $\widehat{\beta}_\tau$ is plotted against τ, with vertical bars representing 95% confidence intervals for $\widehat{\beta}_\tau$. The gray horizontal line with dashed lines above and below represent $\widehat{\beta}_{\mathrm{OLS}}$ and its 95% confidence interval for a linear model fit to residual data as in panel B. In panel D, $\widehat{\beta}_\tau/\widehat{\beta}_{\mathrm{OLS}}$ versus τ is plotted with vertical bars representing 95% confidence intervals for the ratio. Homogeneous effect sizes across the phenotypic distribution correspond to ratios $\widehat{\beta}_\tau/\widehat{\beta}_{\mathrm{OLS}}$ equal to one for each τ. The gray horizontal lines in panel D bound an equivalence band where the ratio is between $(1/\gamma, \gamma)$ for $\gamma = 1.2$. For ratios with the 95% confidence interval completely above or below the band, the vertical bars are colored red to indicate non-equivalence of the QR and OLS effect sizes and thus heterogeneity of $\widehat{\beta}_\tau$ and the linear predictive value of the PGS across the phenotypic range.

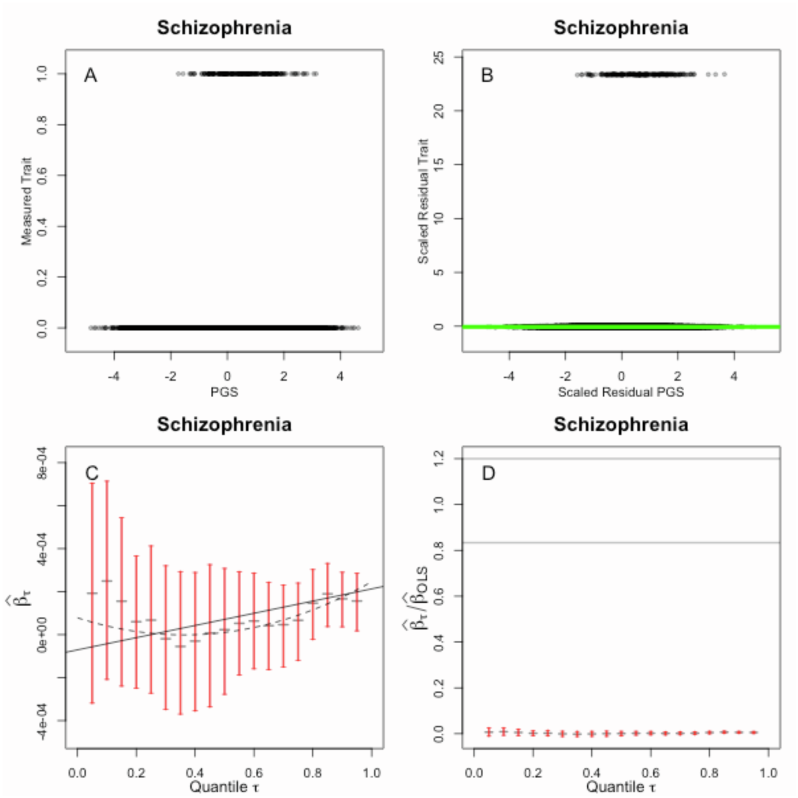

Figure S24. Schizophrenia

Panels A and B show scatter plots of the trait (schizophrenia) versus polygenic score for participants in the UK Biobank. In panel A and trait and PGS as reported in the data set are plotted. In panel B, residualized and scaled trait and PGS values are used, after projection of age, age^2, sex, age*sex, and ten genetic principal components and rescaling the residuals to mean zero variance one. The green lines show linear models fit to the data using QR at the 95th, 50th, and 5th quantiles of the residual trait distribution. QR is used to estimate quantile specific linear effect sizes $\widehat{\beta}_\tau$ at 19 quantiles τ of the residual trait distribution as in panel B. In panel C, $\widehat{\beta}_\tau$ is plotted against τ, with vertical bars representing 95% confidence intervals for $\widehat{\beta}_\tau$. The gray horizontal line with dashed lines above and below represent $\widehat{\beta}_{\mathrm{OLS}}$ and its 95% confidence interval for a linear model fit to residual data as in panel B. In panel D, $\widehat{\beta}_\tau/\widehat{\beta}_{\mathrm{OLS}}$ versus τ is plotted with vertical bars representing 95% confidence intervals for the ratio. Homogeneous effect sizes across the phenotypic distribution correspond to ratios $\widehat{\beta}_\tau/\widehat{\beta}_{\mathrm{OLS}}$ equal to one for each τ. The gray horizontal lines in panel D bound an equivalence band where the ratio is between $(1/\gamma, \gamma)$ for $\gamma = 1.2$. For ratios with the 95% confidence interval completely above or below the band, the vertical bars are colored red to indicate non-equivalence of the QR and OLS effect sizes and thus heterogeneity of $\widehat{\beta}_\tau$ and the linear predictive value of the PGS across the phenotypic range.

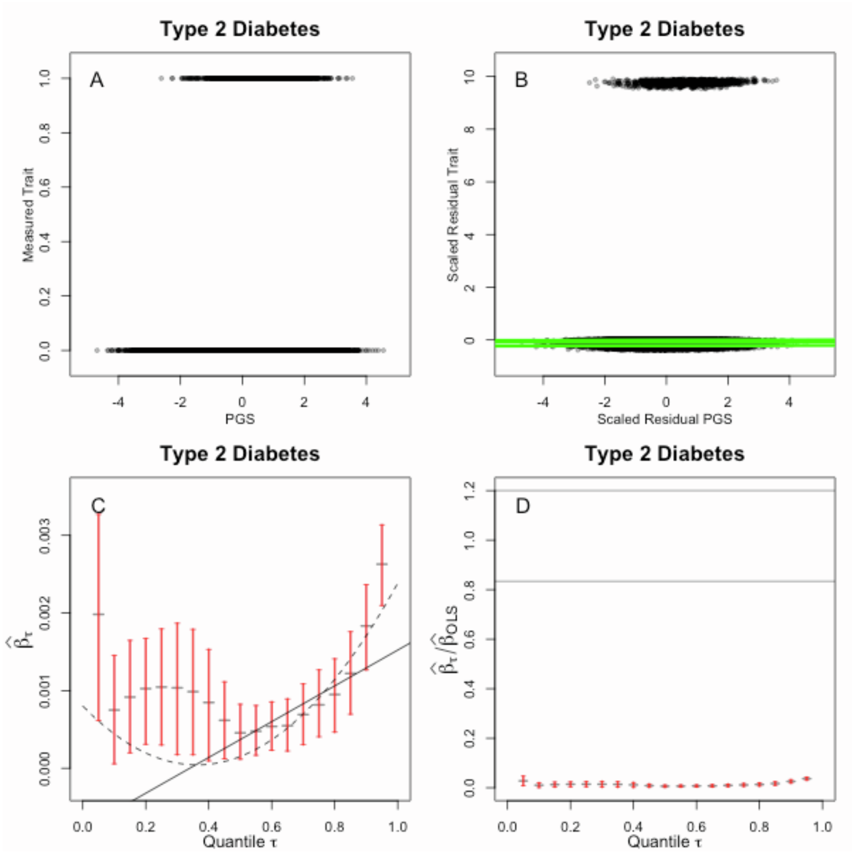

Figure S25. Type 2 diabetes, T2D

Panels A and B show scatter plots of the trait (type 2 diabetes, T2D) versus polygenic score for participants in the UK Biobank. In panel A and trait and PGS as reported in the data set are plotted. In panel B, residualized and scaled trait and PGS values are used, after projection of age, age^2, sex, age*sex, and ten genetic principal components and rescaling the residuals to mean zero variance one. The green lines show linear models fit to the data using QR at the 95th, 50th, and 5th quantiles of the residual trait distribution. QR is used to estimate quantile specific linear effect sizes $\widehat{\beta}_\tau$ at 19 quantiles τ of the residual trait distribution as in panel B. In panel C, $\widehat{\beta}_\tau$ is plotted against τ, with vertical bars representing 95% confidence intervals for $\widehat{\beta}_\tau$. The gray horizontal line with dashed lines above and below represent $\widehat{\beta}_{\rm OLS}$ and its 95% confidence interval for a linear model fit to residual data as in panel B. In panel D, $\widehat{\beta}_\tau/\widehat{\beta}_{\rm OLS}$ versus τ is plotted with vertical bars representing 95% confidence intervals for the ratio. Homogeneous effect sizes across the phenotypic distribution correspond to ratios $\widehat{\beta}_\tau/\widehat{\beta}_{\rm OLS}$ equal to one for each τ. The gray horizontal lines in panel D bound an equivalence band where the ratio is between $(1/\gamma, \gamma)$ for $\gamma = 1.2$. For ratios with the 95% confidence interval completely above or below the band, the vertical bars are colored red to indicate non-equivalence of the QR and OLS effect sizes and thus heterogeneity of $\widehat{\beta}_\tau$ and the linear predictive value of the PGS across the phenotypic range.

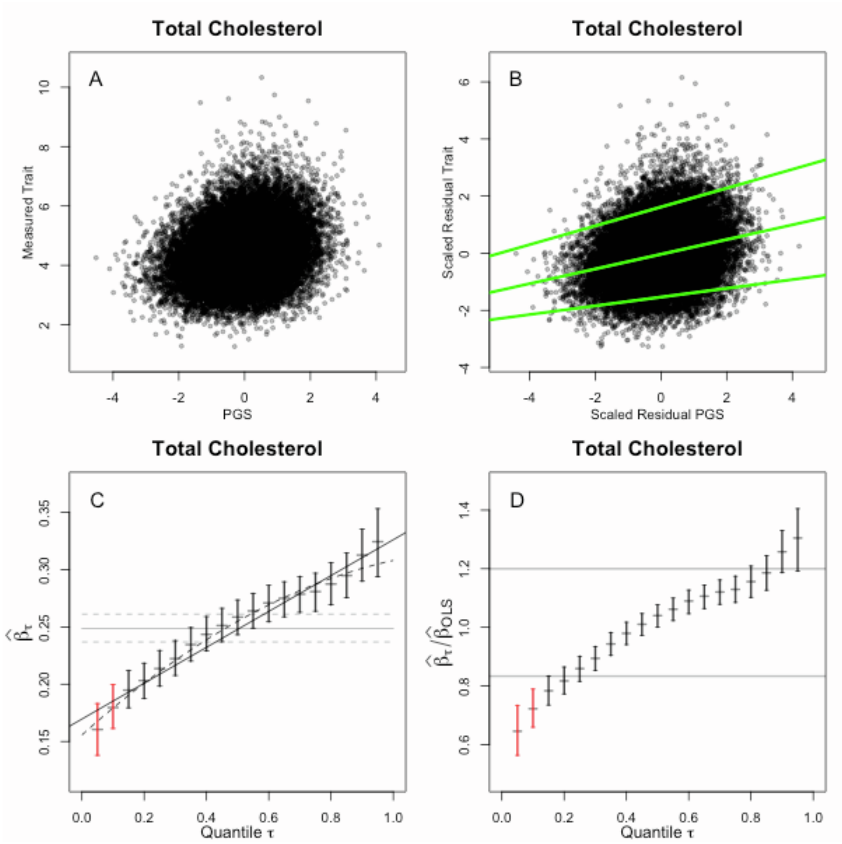

Figure S26. Total cholesterol

Panels A and B show scatter plots of the trait (total cholesterol) versus polygenic score for participants in the UK Biobank. In panel A and trait and PGS as reported in the data set are plotted. In panel B, residualized and scaled trait and PGS values are used, after projection of age, age^2, sex, age*sex, and ten genetic principal components and rescaling the residuals to mean zero variance one. The green lines show linear models fit to the data using QR at the 95th, 50th, and 5th quantiles of the residual trait distribution. QR is used to estimate quantile specific linear effect sizes $\widehat{\beta}_\tau$ at 19 quantiles τ of the residual trait distribution as in panel B. In panel C, $\widehat{\beta}_\tau$ is plotted against τ, with vertical bars representing 95% confidence intervals for $\widehat{\beta}_\tau$. The gray horizontal line with dashed lines above and below represent $\widehat{\beta}_{\mathrm{OLS}}$ and its 95% confidence interval for a linear model fit to residual data as in panel B. In panel D, $\widehat{\beta}_\tau/\widehat{\beta}_{\mathrm{OLS}}$ versus τ is plotted with vertical bars representing 95% confidence intervals for the ratio. Homogeneous effect sizes across the phenotypic distribution correspond to ratios $\widehat{\beta}_\tau/\widehat{\beta}_{\mathrm{OLS}}$ equal to one for each τ. The gray horizontal lines in panel D bound an equivalence band where the ratio is between $(1/\gamma, \gamma)$ for $\gamma = 1.2$. For ratios with the 95% confidence interval completely above or below the band, the vertical bars are colored red to indicate non-equivalence of the QR and OLS effect sizes and thus heterogeneity of $\widehat{\beta}_\tau$ and the linear predictive value of the PGS across the phenotypic range.

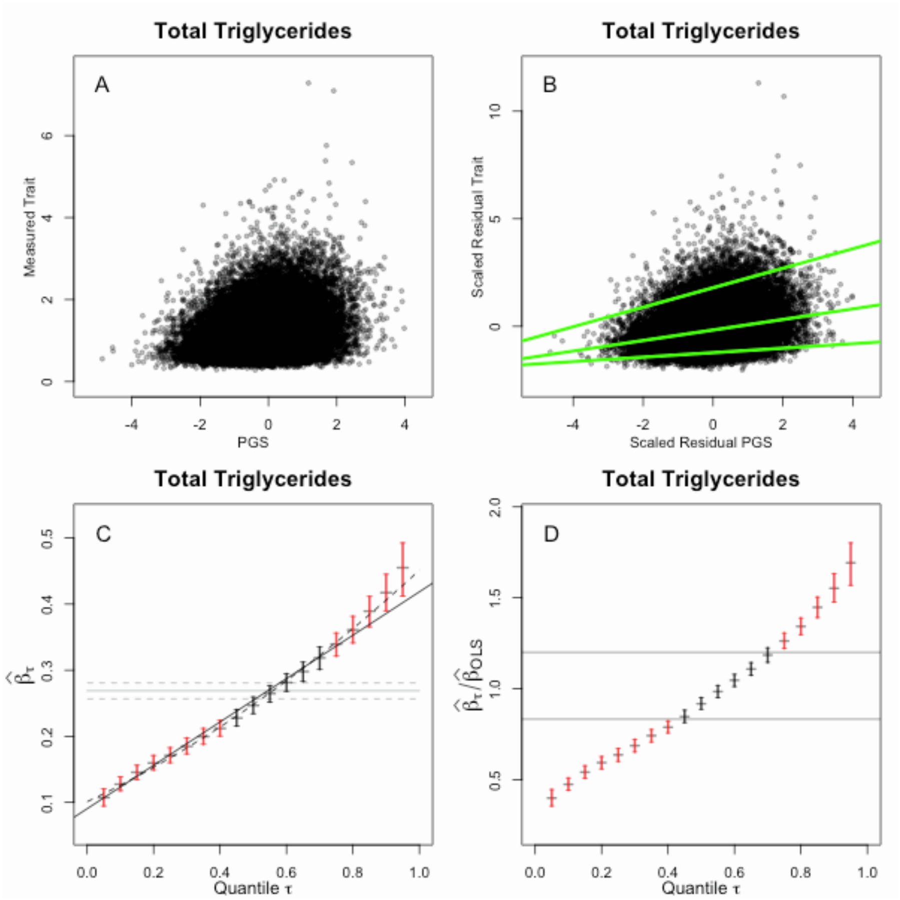

Figure S27. Total triglycerides

Panels A and B show scatter plots of the trait (total triglycerides) versus polygenic score for participants in the UK Biobank. In panel A and trait and PGS as reported in the data set are plotted. In panel B, residualized and scaled trait and PGS values are used, after projection of age, age^2, sex, age*sex, and ten genetic principal components and rescaling the residuals to mean zero variance one. The green lines show linear models fit to the data using QR at the 95th, 50th, and 5th quantiles of the residual trait distribution. QR is used to estimate quantile specific linear effect sizes $\widehat{\beta}_\tau$ at 19 quantiles τ of the residual trait distribution as in panel B. In panel C, $\widehat{\beta}_\tau$ is plotted against τ, with vertical bars representing 95% confidence intervals for $\widehat{\beta}_\tau$. The gray horizontal line with dashed lines above and below represent $\widehat{\beta}_{\mathrm{OLS}}$ and its 95% confidence interval for a linear model fit to residual data as in panel B. In panel D, $\widehat{\beta}_\tau/\widehat{\beta}_{\mathrm{OLS}}$ versus τ is plotted with vertical bars representing 95% confidence intervals for the ratio. Homogeneous effect sizes across the phenotypic distribution correspond to ratios $\widehat{\beta}_\tau/\widehat{\beta}_{\mathrm{OLS}}$ equal to one for each τ. The gray horizontal lines in panel D bound an equivalence band where the ratio is between $(1/\gamma, \gamma)$ for $\gamma = 1.2$. For ratios with the 95% confidence interval completely above or below the band, the vertical bars are colored red to indicate non-equivalence of the QR and OLS effect sizes and thus heterogeneity of $\widehat{\beta}_\tau$ and the linear predictive value of the PGS across the phenotypic range.

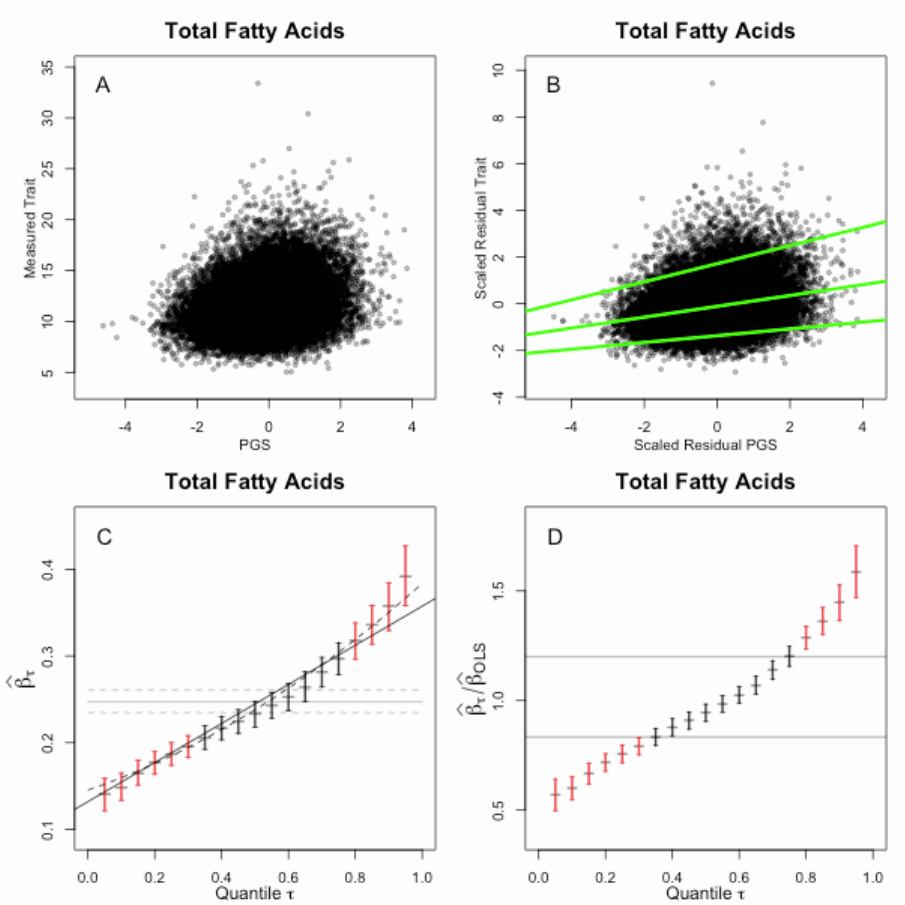

Figure S28. Total fatty acids

Panels A and B show scatter plots of the trait (total fatty acids) versus polygenic score for participants in the UK Biobank. In panel A and trait and PGS as reported in the data set are plotted. In panel B, residualized and scaled trait and PGS values are used, after projection of age, age^2, sex, age*sex, and ten genetic principal components and rescaling the residuals to mean zero variance one. The green lines show linear models fit to the data using QR at the 95th, 50th, and 5th quantiles of the residual trait distribution. QR is used to estimate quantile specific linear effect sizes $\widehat{\beta}_\tau$ at 19 quantiles τ of the residual trait distribution as in panel B. In panel C, $\widehat{\beta}_\tau$ is plotted against τ, with vertical bars representing 95% confidence intervals for $\widehat{\beta}_\tau$. The gray horizontal line with dashed lines above and below represent $\widehat{\beta}_{\mathrm{OLS}}$ and its 95% confidence interval for a linear model fit to residual data as in panel B. In panel D, $\widehat{\beta}_\tau/\widehat{\beta}_{\mathrm{OLS}}$ versus τ is plotted with vertical bars representing 95% confidence intervals for the ratio. Homogeneous effect sizes across the phenotypic distribution correspond to ratios $\widehat{\beta}_\tau/\widehat{\beta}_{\mathrm{OLS}}$ equal to one for each τ. The gray horizontal lines in panel D bound an equivalence band where the ratio is between $(1/\gamma, \gamma)$ for $\gamma = 1.2$. For ratios with the 95% confidence interval completely above or below the band, the vertical bars are colored red to indicate non-equivalence of the QR and OLS effect sizes and thus heterogeneity of $\widehat{\beta}_\tau$ and the linear predictive value of the PGS across the phenotypic range.

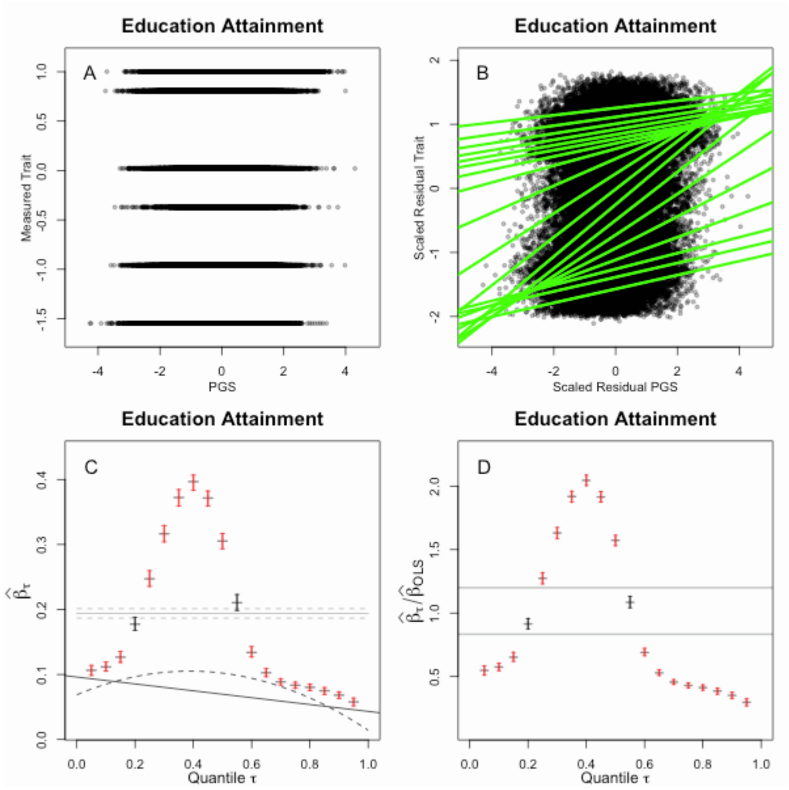

Figure S29. Education Attainment

Educational attainment (EA, years) takes a small number of integer values corresponding to completion of primary school, secondary school, college degree and so on. So, education-years has a distinctly multimodal distribution. These education-years were z-transformed to give the trait values in Panel A. As with case-control traits, the OLS and quantile regression effect estimates for the PGS, $\widehat{\beta}_{\mathrm{OLS}}$ and $\widehat{\beta}_{\tau}$, show heterogeneity of $\widehat{\beta}_{\tau}$ related to the proportions of observations in each mode represented by the horizontal lines in the scatter plot of panel A. Perhaps future work could find a way to quantify and interpret the relatively strong impact of a genetic boost to the liability of moving from one mode of the EA distribution to another for individuals whose non-genetic contributions put them near a threshold between modes. This would generalize the analyses of case-control traits to multimodal traits, but further work is needed to make the quantile-regression and non-equivalence test approach informative for case-control traits as well.

Panels A and B show scatter plots of the trait (education attainment) versus polygenic score for participants in the UK Biobank. In panel A and trait and PGS as reported in the data set are plotted. In panel B, residualized and scaled trait and PGS values are used, after projection of age, age^2, sex, age*sex, and ten genetic principal components and rescaling the residuals to mean zero variance one. QR is used to estimate and plot quantile specific linear models at 19 quantiles τ of the residual trait distribution. In panel C, $\widehat{\beta}_{\tau}$ is plotted against τ, with vertical bars representing 95% confidence intervals for $\widehat{\beta}_{\tau}$. The gray horizontal line with dashed lines above

and below represent $\widehat{\beta}_{\mathrm{OLS}}$ and its 95% confidence interval for a linear model fit to residual data as in panel B. In panel D, $\widehat{\beta}_{\tau}/\widehat{\beta}_{\mathrm{OLS}}$ versus τ is plotted with vertical bars representing 95% confidence intervals for the ratio. Homogeneous effect sizes across the phenotypic distribution correspond to ratios $\widehat{\beta}_{\tau}/\widehat{\beta}_{\mathrm{OLS}}$ equal to one for each τ. The gray horizontal lines in panel D bound an equivalence band where the ratio is between $(1/\gamma, \gamma)$ for $\gamma = 1.2$. For ratios with the 95% confidence interval completely above or below the band, the vertical bars are colored red to indicate non-equivalence of the QR and OLS effect sizes and thus heterogeneity of $\widehat{\beta}_{\tau}$ and the linear predictive value of the PGS across the phenotypic range.

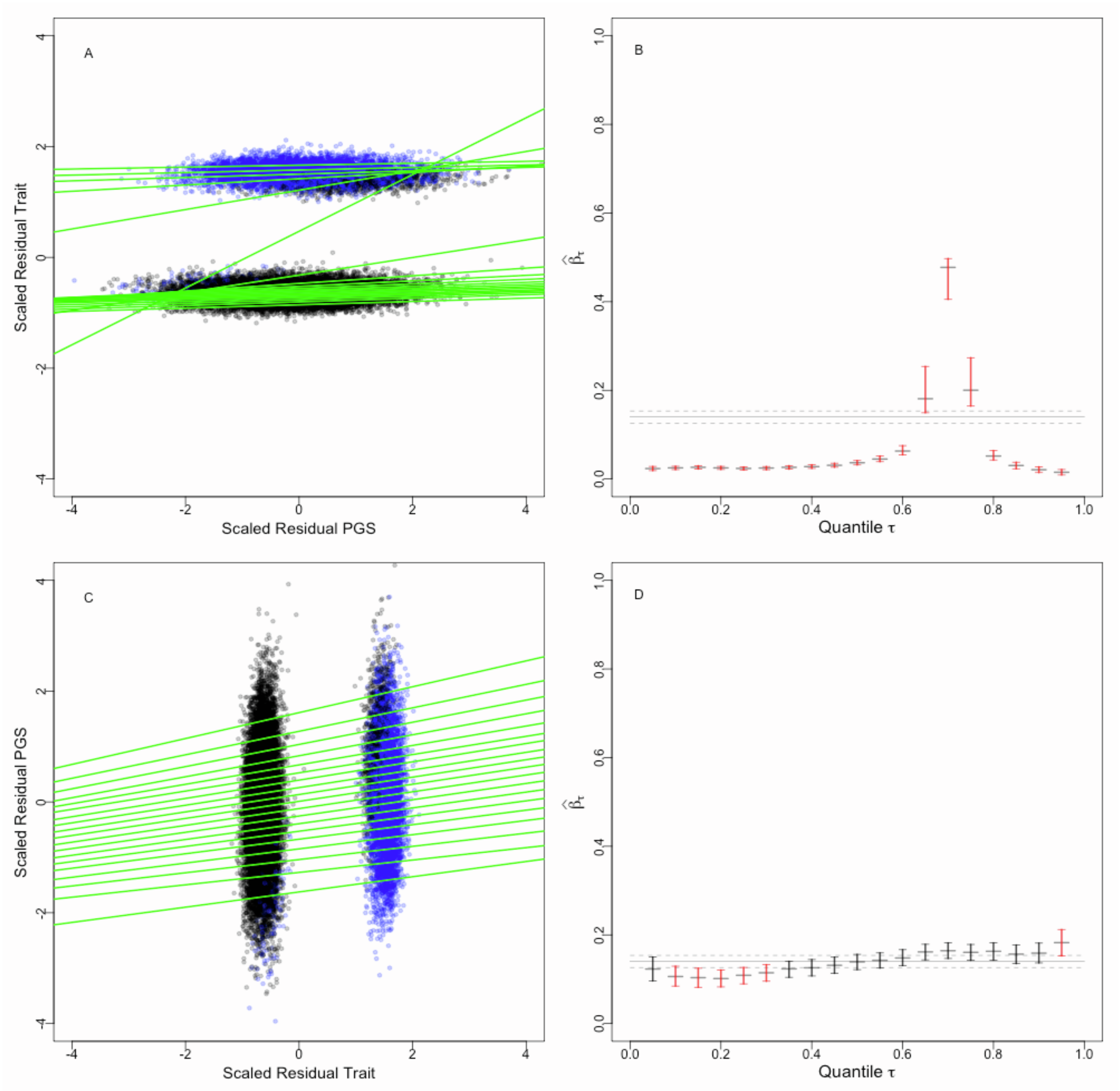

Figure S30. Transposing the analysis for case-control data

Figures S30 and S31 are results from analysis of a case control data set corresponding to the data of Figure 4P (S31) and another bimodal dataset with better separated modes (S30, $\gamma = 5$). Here the continuous trait analyzed for Figure 4A and 4B was treated as a liability for the case-control trait in S31. Figure S30 is for a trait with better separated liabilities for the cases and controls. Panel S30B shows a spike at quantile τ=0.7, corresponding to the 0.7 control proportion in the simulated dataset or the 1-0.7=0.3 case proportion.

Panels S30C and S30D show the same data as in panels A and B, but with the axes transposed. With these scales, quantile regression models the quantiles of the continuous PGS distribution rather than the dichotomous trait. Comparing panels S30B and D, or S30D and S31D, we don't see any easy way to interpret these results in terms of exposure or interactions

as we have done for other deviations from homogeneity. We do not see any way to interpret the results of the transposed analysis in terms of case-control proportions either.

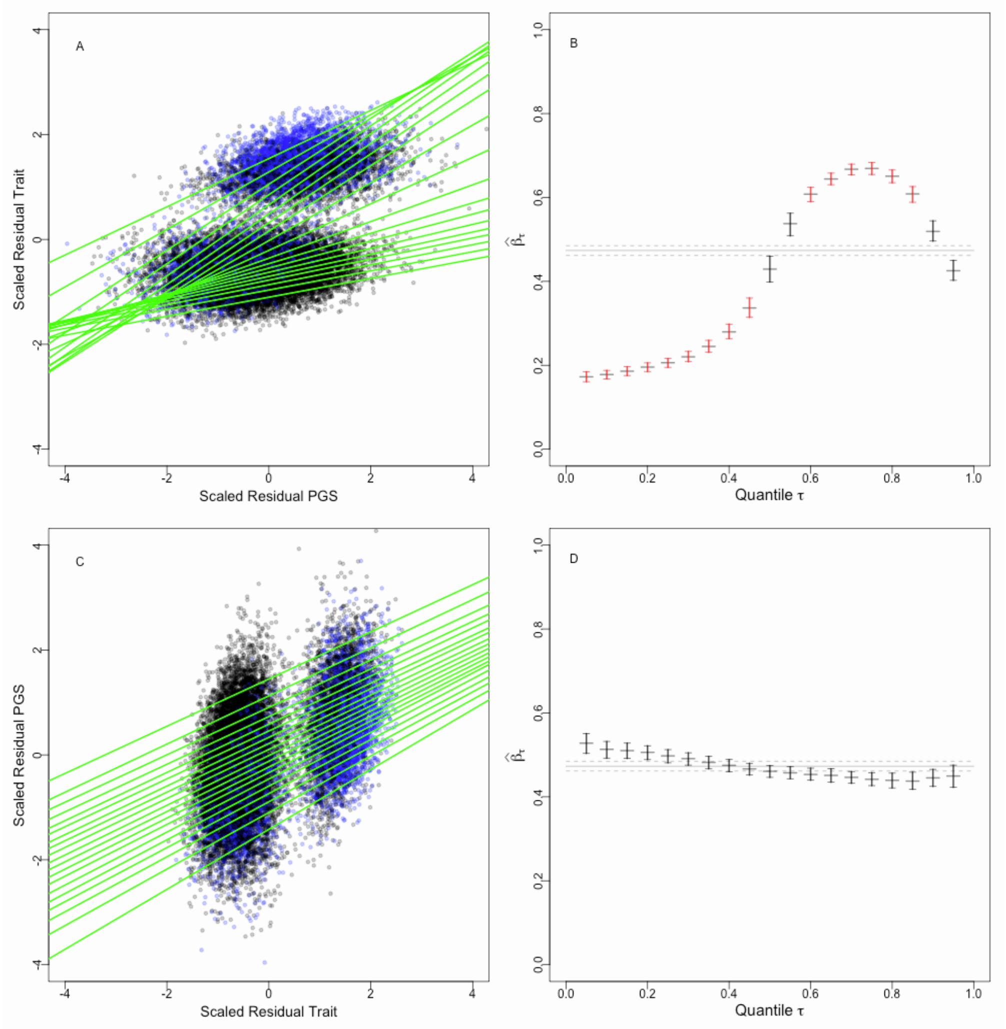

Figure S31. Transposing the analysis for bimodal data

Figures S30 and S31 are results from analysis of a case control data set corresponding to the data of Figure 4P (S31) and another bimodal dataset with better separated modes (S30, $\gamma = 5$). Here the continuous trait analyzed for Figure 4A and 4B was treated as a liability for the case-control trait in S31. Figure S30 is for a trait with better separated liabilities for the cases and controls. Panel S31B shows a mode at quantile τ=0.7, corresponding to the 0.7 control proportion in the simulated dataset or the 1-0.7=0.3 case proportion. This mode is broader than the one in panel S30B due to the less well-separated modes in Figure S31 than in S29.

Panels S31C and S31D show the same data as in panels A and B, but with the axes transposed. With these scales, quantile regression models the quantiles of the continuous PGS distribution rather than the dichotomous trait. Comparing panels S31B and D, or S31D and

S30D, we don't see any easy way to interpret these results in terms of exposure or interactions as we have done for other deviations from homogeneity. We do not see any way to interpret the results of the transposed analysis in terms of case-control proportions either.

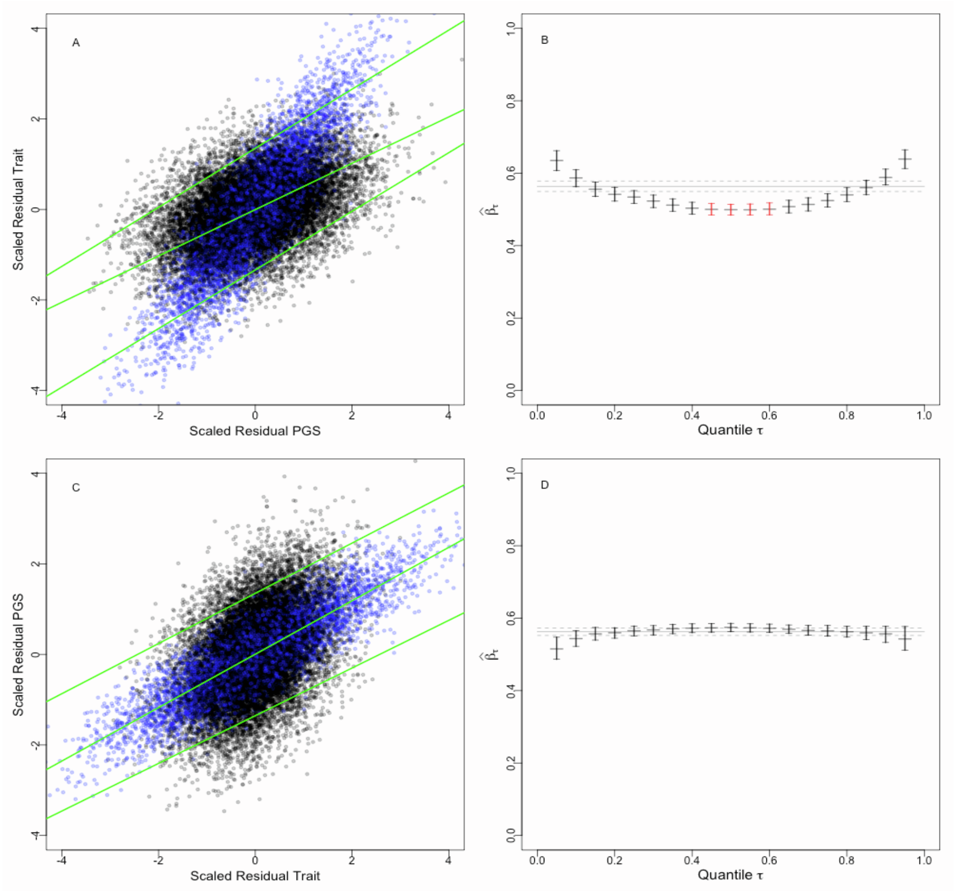

Figure S32. Transposing the analysis for the $G \times E$ model

For S32 and S33 we compare the standard analyses to the transposed analysis for continuous traits. In the residual analysis we use (residual trait vs residual PGS) with rescaled residuals, the OLS regression coefficient is also the correlation. In the transposed analysis, the OLS regression coefficient is again the correlation The quantile-specific linear model effect sizes are then analogous to correlations or OLS effect sizes. Specifically, for the analyses in this paper, $\widehat{\beta}_{\tau}$ is the slope of a trend line through the τth percentile of residual outcome vs residual PGS. The simulations for Figure 4 suggest how patterns in plots of $\widehat{\beta}_{\tau}$ versus τ reflect the data generating model – directions and strengths of GxE interactions and E main effects, and the proportion PrE of observations having a dichotomous exposure. For a "turned around analysis", $\widehat{\beta}_{\tau}$ is the slope of a trend line through the τth percentile of residual PGS vs residual PGS. In

supplemental figures S32 and S33, data with pronounced GxE interactions is analyzed by our standard trait-vs-PGS approach in Panels A and B, then by the turned-around method for Panels C and D. For the standard analysis, significant non-equivalence of $\widehat{\beta}_\tau$ and the OLS effect size is shown, and the patterns on $\widehat{\beta}_\tau$ versus τ suggest a GxE interaction with negligible E main effect for S32, and a GxE interaction with an E main effect both increasing trait values in the exposed observations and an exposed proportion around 0.3. For the turned around analyses, no $\widehat{\beta}_\tau$ are flagged as non-equivalent to the OLS estimate, and we don't see any we cannot see obvious patterns in the $\widehat{\beta}_\tau$ versus τ to give insight into the data generating model. There may be some other informative way to use quantile regression to characterize disease polygenic risk scores

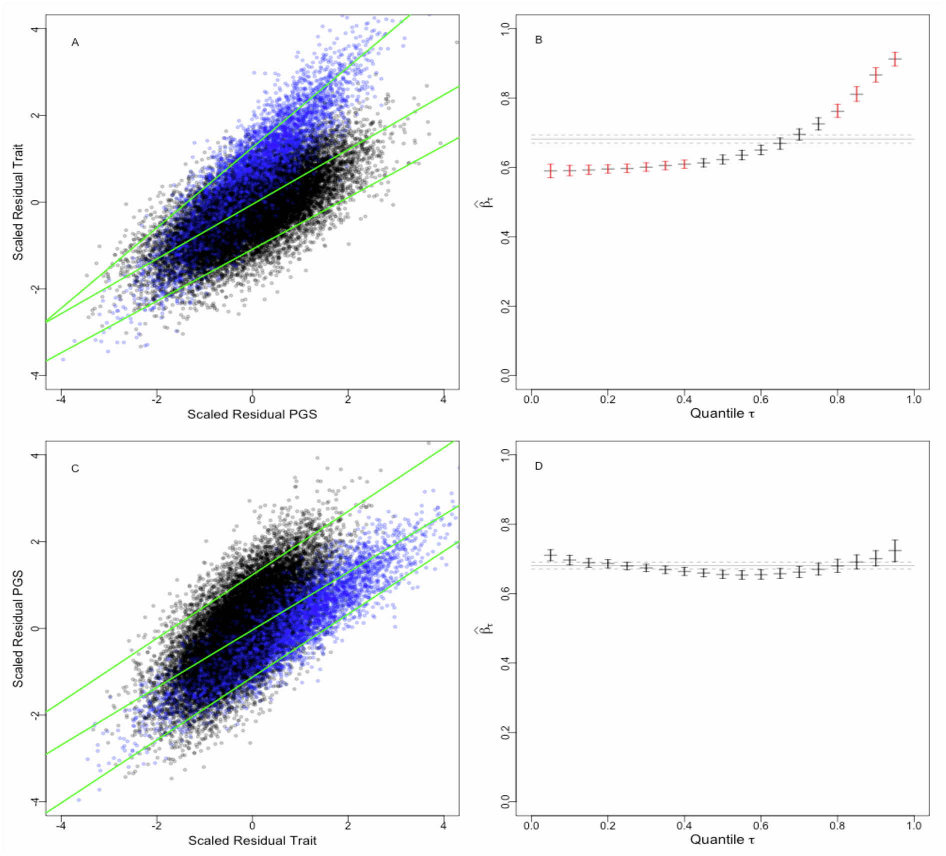

Figure S33. Transposing the analysis for the $G \times E$ model with an E main effect

For S32 and S33 we compare the standard analyses to the transposed analysis for continuous traits. In the residual analysis we use (residual trait vs residual PGS) with rescaled residuals, the OLS regression coefficient is also the correlation. In the transposed analysis, the OLS regression coefficient is again the correlation The quantile-specific linear model effect sizes are then analogous to correlations or OLS effect sizes. Specifically, for the analyses in this paper, $\widehat{\beta}_\tau$ is the slope of a trend line through the τth percentile of residual outcome vs residual PGS. The simulations for Figure 4 suggest how patterns in plots of $\widehat{\beta}_\tau$ versus τ reflect the data generating model – directions and strengths of GxE interactions and E main effects, and the proportion PrE of observations having a dichotomous exposure. For a "turned around analysis", $\widehat{\beta}_\tau$ is the slope of a trend line through the τth percentile of residual PGS vs residual PGS. In supplemental figures S32 and S33, data with pronounced GxE interactions is analyzed by our standard trait-vs-PGS approach in Panels A and B, then by the turned-around method for Panels C and D. For the standard analysis, significant non-equivalence of $\widehat{\beta}_\tau$ and the OLS effect size is shown, and the patterns on $\widehat{\beta}_\tau$ versus τ suggest a GxE interaction with a substantial E main effect for S33, with the GxE interaction and E main effect both increasing

trait values in the exposed observations and an exposed proportion around 0.3. For the turned around analyses, no $\widehat{\beta}_{\tau}$ are flagged as non-equivalent to the OLS estimate, and we don't see any we cannot see obvious patterns in the $\widehat{\beta}_{\tau}$ versus τ to give insight into the data generating model. There may be some other informative way to use quantile regression to characterize disease polygenic risk scores

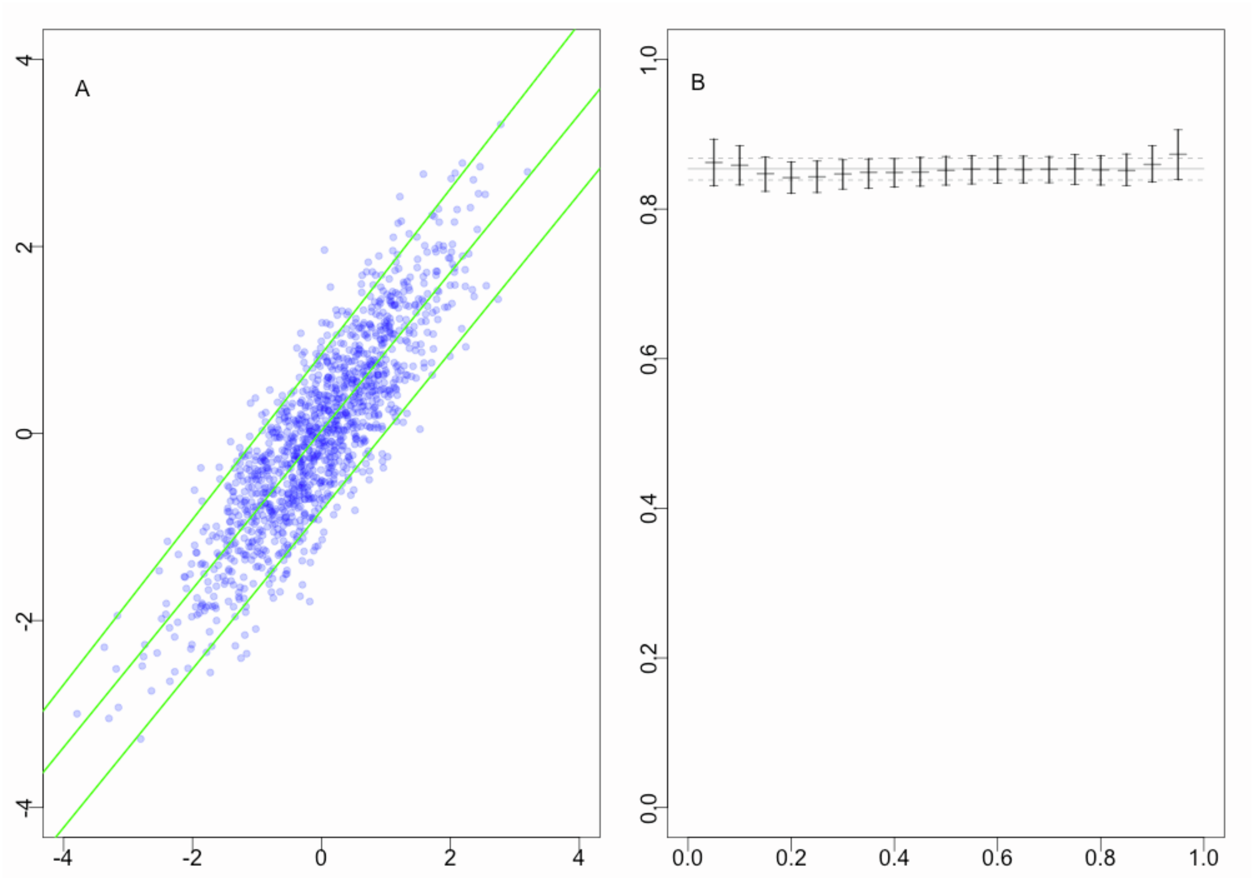

Figure S34. Reference Polygenic Scores

Supplemental Figure S34 illustrates the effect of using an 'off-the-shelf' polygenic score (rather than one generated from the same data) For Figure 5 Panels E-H, the GWAS and PGS development was done separately in the exposed (5E and 5F) and non-exposed (5G and 5H) strata, however, using a polygenic score developed on a mixed set of exposed and unexposed observations but following with the quantile regression and non-equivalence tests in a single partition also results in homogeneous $\widehat{\beta}_{\tau}$ in the stratum specific analyses. So, the confirmatory analyses for checking the contribution of a particular exposure to an observed pattern of heterogeneous $\widehat{\beta}_{\tau}$, does not require access to the genetic source data for a new GWAS run in the stratum of interest, and development of a new PGS. However, removal of one source of trait heterogeneity and stronger PGS predictive value in one stratum suggests further analyses of each stratum separately may be informative.

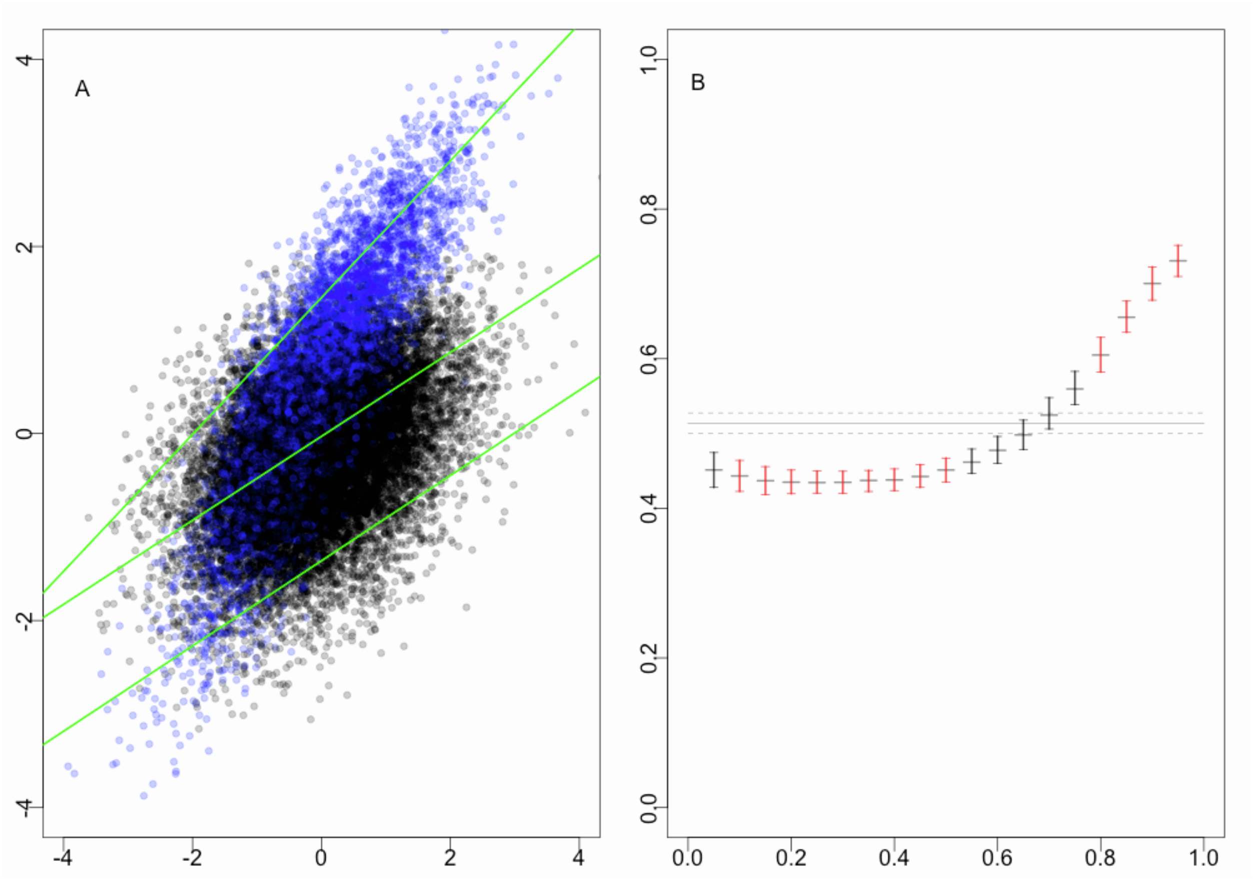

Figure S35. Data transformation

In Figure S35, the same data that was analyzed for Figure 5 was used. The contrast between Figure 5 Panels A and B and Figure S35, is that to generate the later figure, the trait was quantile normalized before analysis. The trait was quantile normalized before the GWAS, so SNP effect sizes and the PGS do not have the same interpretation as in the non-transformed analyses in Figure 5A and 5B, but the plots of $\widehat{\beta}_\tau$ versus quantile τ are qualitatively the same for the analyses of the non-transformed and transformed data (uniformly low $\widehat{\beta}_\tau$ values at low quantiles below 0.5, sloping up to higher values at higher quantiles, crossing the OLS effect size estimate around 0.75) . Here, the data generating model corresponds to the non-transformed analysis, which may explain the somewhat higher power for the non-equivalence tests using the non-transformed data – more $\widehat{\beta}_\tau$ quantiles are significantly non-equivalent to the OLS estimate with the non-transformed analysis.

Table S1. Quantile-specific and OLS predictive R^2

Trait	Trait code	R2 OLs	R2 min	R2 max	R2 min/ R2 OLS	R2 max / R2 OLS	Max $\widehat{\beta}_{\tau}/\widehat{\beta}_{\mathrm{OLS}}$	Min $\widehat{\beta}_{\tau}/\widehat{\beta}_{\mathrm{OLS}}$
Age At Menopause	AAM	0.053	0.028	0.111	0.523	2.078	0.723	1.442
Apolipoprotein A1	APOEA	0.097	0.054	0.191	0.559	1.972	0.748	1.404
Apolipoprotein B	APOEB	0.107	0.048	0.211	0.453	1.966	0.673	1.402
Body Mass Index	BMI	0.119	0.033	0.355	0.273	2.973	0.523	1.724
Calcium	CAL	0.065	0.056	0.075	0.864	1.160	0.930	1.077
Docosahexaenoic Acid	DOA	0.050	0.035	0.083	0.699	1.651	0.836	1.285
Estimated Bone Mineral Density T Score	EBMDT	0.016	0.001	0.067	0.042	4.172	0.204	2.043
Estimated Glomerular Filtration Rate (Creatinine Based)	EGCR	0.040	0.057	0.026	1.422	0.641	1.192	0.801
Estimated Glomerular Filtration Rate (Cystatin Based)	EGCY	0.073	0.124	0.043	1.701	0.589	1.304	0.768
Glycated Haemoglobin	HBA1C	0.058	0.039	0.197	0.673	3.409	0.821	1.846
Height	HEIGHT	0.302	0.264	0.323	0.875	1.071	0.936	1.035
High Density Lipoprotein Cholesterol	HDL	0.157	0.076	0.311	0.487	1.984	0.698	1.409
Intraocular Pressure	IOP	0.032	0.019	0.045	0.598	1.400	0.774	1.183
Low Density Lipoprotein Cholesterol	LDL	0.094	0.026	0.195	0.282	2.077	0.531	1.441
Omega 3 Fatty Acids	OTFA	0.075	0.040	0.138	0.529	1.831	0.727	1.353
Omega 6 Fatty Acids	OSFA	0.062	0.021	0.143	0.334	2.292	0.578	1.514
Phosphatidylcholines	PDCL	0.083	0.042	0.150	0.507	1.792	0.712	1.339
Phosphoglycerides	PHG	0.075	0.034	0.141	0.459	1.888	0.677	1.374
Polyunsaturated Fatty Acids	PFA	0.070	0.029	0.136	0.417	1.950	0.646	1.396

Remnant Cholesterol (Non Hdl, Non Ldl Cholesterol)	RMNC	0.059	0.022	0.107	0.372	1.827	0.610	1.352
Resting Heart Rate	RHR	0.065	0.041	0.097	0.640	1.502	0.800	1.226
Sphingomyelins	SGM	0.076	0.039	0.134	0.517	1.772	0.719	1.331
Total Cholesterol	TCH	0.062	0.023	0.109	0.381	1.763	0.617	1.328
Total Fatty Acids	TFA	0.061	0.021	0.151	0.343	2.452	0.586	1.566
Total Triglycerides	TTG	0.072	0.011	0.206	0.150	2.855	0.388	1.690
