# Supplementary material for: Beyond predictive R2: Quantile regression and non-equivalence tests reveal complex relationships of traits and polygenic scores
Source: Am J Hum Genet. 2025 Jun 5;112(6):1363–75. doi: 10.1016/j.ajhg.2025.04.013 (PMC12256909; doi:10.1016/j.ajhg.2025.04.013)
Supplement: Document S2. Article plus supplemental information [file mmc2.pdf]

Beyond predictive R^2: Quantile regression and non-equivalence tests reveal complex relationships of traits and polygenic scores

Authors

Joel Mefford, Molly Smullen, Felix Zhang, ..., Andy Dahl, Jonathan Flint, Noah Zaitlen

Correspondence

jmefford@mednet.ucla.edu

We quantify differences in the predictive value of polygenic scores (PGSs) across the phenotypic range using quantile regression and non-equivalence tests of quantile-specific effect sizes. We demonstrate that heterogeneity can arise from gene-by-environment interactions. Our approach finds traits with evidence of interactions and heterogeneous PGS performance without specifying the relevant environmental exposures.

Mefford et al., 2025, The American Journal of Human Genetics *112*, 1363–1375
June 5, 2025 © 2025 The Authors. Published by Elsevier Inc. on behalf of American Society of Human Genetics.
https://doi.org/10.1016/j.ajhg.2025.04.013

CellPress

Beyond predictive R^2: Quantile regression and non-equivalence tests reveal complex relationships of traits and polygenic scores

Joel Mefford,[1,*] Molly Smullen,[2] Felix Zhang,[3] Michal Sadowski,[4] Richard Border,[3,5,6] Andy Dahl,[7] Jonathan Flint,[1,8] and Noah Zaitlen[5,6,9]

Summary

Polygenic scores (PGSs) are genetic predictions of trait values or disease risk that are increasingly finding applications in clinical predictive models and basic genetics research. However, the predictive value of a PGS can vary within similar population groups, depending on characteristics such as the environmental exposures, sex, age, or socioeconomic status of the individuals. To maximize the value of a PGS, approaches to screen trait-PGS pairs for evidence of such heterogeneity without having to specify the relevant exposure or individual characteristics would be useful. Here, in analyses from the UK Biobank, we show that a PGS's predictive accuracy depends on the quantile of the phenotypic distribution to which the PGS is being applied. We quantify differences in predictive value across the phenotypic range using quantile regression linear models to estimate quantile-specific effect sizes for linear models of phenotype values as a function of PGS. Of 25 continuous traits, only three have no quantile-specific effect sizes that varied by at least 1.2-fold from the ordinary least squares estimate. Through simulation, we demonstrate that this heterogeneity of PGS predictive value can arise from gene-by-environment interactions. Our approach can be used to flag traits where the use of PGSs warrants extra caution, and perhaps stratification variables should be sought and used because PGSs perform substantially differently in portions of the sampled population than expected from quoted predictive R^2 or incremental R^2 values that represent average performance across a dataset.

Introduction

The use of polygenic scores (PGSs) as genetic predictors of complex traits has attracted considerable interest for their potential value in personalized health by predicting the risk of developing common diseases[1–6] and for their use as basic research instruments.[7–10] The predictive value of a PGS depends on several factors, some of which have been individually studied, including how well the ancestry of the population in which the PGS was developed matches the target individual's[11] sex, age, and socioeconomic status,[12] genotyping error,[13] and trait-specific factors, such as the use of a particular medication.[14]

Often, the predictive value of a PGS is summarized with an R^2 value from a regression model for phenotype value as a function of the PGS or by the incremental improvement of R^2 when PGS is added to a baseline model. However, the value of R^2 as a summary of the mean predictive value of a PGS is limited if there are strata within a dataset where the predictive value is well above or below the mean. If the appropriate stratification variables are known or suspected and measured, then subsets of the dataset can be analyzed separately to characterize the heterogeneous predictive value of the PGS across strata, but an approach to screen trait-PGS pairs for evidence of such heterogeneity without having to specify appropriate stratification variables would be useful. In this study, we propose a method to screen trait-PGS pairs for evidence of heterogeneity in predictive value without such knowledge. Instead of heterogeneity of predictive value across strata, we consider heterogeneity of predictive value across the phenotypic range.

Identifying trait-PGS pairs with heterogeneous predictive value is important because assuming that a mean predictive value such as an R^2 value from ordinary least squares (OLS) applies to an entire population may greatly over- or under-estimate the actual predictive value for subsets of the population. We characterize how genetic effects vary across the phenotypic distribution by examining quantile-specific effect sizes from linear models estimated using quantile regression (QR) and contrasting the effect sizes to those estimated by OLS.

Material and methods

Data

Data from the UK Biobank (UKBB)[15] were used to investigate the predictive performance of PGSs for the 25 phenotypes shown in

[1]Semel Institute for Neuroscience and Human Behavior, University of California, Los Angeles, Los Angeles, CA, USA; [2]Chan Medical School, University of Massachusetts, Worcester, MA, USA; [3]Department of Computer Science, University of California, Los Angeles, Los Angeles, CA, USA; [4]Bioinformatics Interdepartmental Program, University of California, Los Angeles, Los Angeles, CA, USA; [5]Department of Neurology, University of California, Los Angeles, Los Angeles, CA, USA; [6]Department of Computational Medicine, David Geffen School of Medicine, University of California, Los Angeles, Los Angeles, CA, USA; [7]Section of Genetic Medicine, Department of Medicine, University of Chicago, Chicago, IL, USA; [8]Department of Psychiatry and Biobehavioral Sciences, David Geffen School of Medicine, University of California, Los Angeles, Los Angeles, CA, USA; [9]Department of Human Genetics, David Geffen School of Medicine, University of California, Los Angeles, Los Angeles, CA, USA
*Correspondence: jmefford@mednet.ucla.edu
https://doi.org/10.1016/j.ajhg.2025.04.013.
© 2025 The Authors. Published by Elsevier Inc. on behalf of American Society of Human Genetics.
This is an open access article under the CC BY license (http://creativecommons.org/licenses/by/4.0/).

Table 1. UKBB data were accessed under application 33127 and are available through the UKBB Access Management System (http://amsportal.ukbiobank.ac.uk/). The PGSs for continuous traits and education attainment calculated in Thompson et al.[2] and the corresponding phenotypes were used for each analysis. These PGSs are based on SNP effect sizes from genome-wide association studies (GWASs) that do not include the UKBB study participants for whom the predictive value of the PGSs was analyzed in this study. PGSs and phenotypes are available via application to the UKBB's Research Access Platform (Resource 5202, https://biobank.ndph.ox.ac.uk/ukb/refer.cgi?id=5202).

OLS and QR

The R package quantreg v.5.95[16] was used for QR analyses. Statistical analyses were adjusted using age, age^2, sex, age*sex, and 10 genetic principal components. The number of study participants for analysis of each phenotype ranged from 9,000 to 104,000.

We developed a residualization process to reduce the QR problem to a univariate regression model: phenotype* $\sim$ PGS*. Here, phenotype* and PGS* were the residuals from linear regression of phenotype $\sim$ adjustment covariates and PGS $\sim$ adjustment covariates, respectively. So, the QR analyses do not consider quantile-specific effect sizes for the adjustment covariates.

OLS was used to estimate $\widehat{\beta}_{\text{OLS}}$ from the regression of residual phenotypes on residual PGSs. QR was used to estimate the quantile-specific effect sizes $\widehat{\beta}_\tau$ for 19 evenly spaced quantiles τ: 0.05, 0.1, … 0.95. The m-of-n bootstrap was used to estimate the covariances of $\widehat{\beta}_{\text{OLS}}$ and each of the $\widehat{\beta}_\tau$, as well as the ratios $\widehat{\beta}_\tau / \widehat{\beta}_{\text{OLS}}$.

In the m-of-n bootstrap, a set of m observations with $m < n$ is sampled, with replacement from the n observations available. The covariances of the effect size estimates and ratios are calculated from the m bootstrap samples and multiplied by m/n to give an estimate of the sampling covariance of the effect size estimates and ratios. The bootstrap sample size m was chosen to be 10,000 or the number of observations available, n, if $n < 10{,}000$.

Predictive value of PGSs

Residuals were scaled to have mean = 0 and variance = 1 before OLS and QR, so the estimated OLS effect size $\widehat{\beta}_{\text{OLS}}$ is the correlation between the residual trait and residual PGS, and effect quantile-specific effect sizes $\widehat{\beta}_\tau$ are analogous to correlations. Further, these effect sizes are squared to represent the OLS summary value for predictive R^2 of the PGS and the analogous predictive R^2_τ at a particular quantile τ. As predictive R^2 for a PGS is related to (upper bounded by) the heritability, the relative values of $\widehat{\beta}_\tau$ and $\widehat{\beta}^2_\tau = R^2_\tau$ at different quantiles indicate the relative predictive value of the PGS or the relative heritability of the trait across quantiles.

Trend tests

The quantile-specific effect sizes $\widehat{\beta}_\tau$ and their estimated sampling covariance were used to test for linear and quadratic trends $\widehat{\beta}_\tau \sim \tau$ and $\widehat{\beta}_\tau \sim \tau + \tau^2$ using the R package metafor v.4.2.0[17] as in Abadi et al.[18] Likelihood ratio tests were used to compare the quadratic and linear fits to the effect-size versus quantile plots and identify phenotypes with statistically significantly better fits with the quadratic trend than the linear trend.

Non-equivalence test

We test whether the QR regression coefficients $\widehat{\beta}_\tau$ are substantially different from those estimated by OLS, $\widehat{\beta}_{\text{OLS}}$, using a non-equivalence test.[19] Also known as the minimum-effects test, this analysis uses two one-sided tests to determine whether an estimate is either greater than a specified upper bound or less than a lower bound. If either null is rejected, the non-equivalence test indicates that the estimate likely lies outside the band defined by the two bounds. To account for the uncertainty in the OLS estimate as well as $\widehat{\beta}_\tau$, we test whether a confidence interval around the ratio $\widehat{\beta}_\tau / \widehat{\beta}_{\text{OLS}}$ is outside of an equivalence band $(1/\lambda, \lambda)$. Since both edges of the band are considered, testing whether a two-sided 95% confidence interval is outside the band corresponds to a non-equivalence test with level $\alpha = 0.10$. These ratios can be numerically unstable or uninterpretable if $\widehat{\beta}_{\text{OLS}}$ is indistinguishable from zero or of opposite sign from $\widehat{\beta}_\tau$. We assume that the analyses described here will be conducted on traits that are significantly heritable and with PGSs that have positive and statistically significant $\widehat{\beta}_{\text{OLS}}$.

The maximum and minimum ratios $\widehat{\beta}_\tau / \widehat{\beta}_{\text{OLS}}$ are collected to illustrate the magnitude of changes in the predictive value of PGSs across the phenotypic range.

Simulations

We run simulations to demonstrate data-generating processes that result in the observed patterns for the QR effect sizes.

For each simulation, data were generated from the following model:

$$y = C\alpha + X\beta + E\gamma + (X \circ E)\Delta + \epsilon. \qquad \text{(Equation 1)}$$

Here, the quantitative phenotype y depends on a set of covariates C, genotypes X, and environmental exposures E, with effect sizes α, β, and γ, respectively. We focus on dichotomous exposures, or, equivalently, subtype indicators, for E. Direct products of E with columns of X, $(X \circ E)$, represent $G \times E$ interactions with effect sizes Δ. Finally, ϵ is normally distributed noise.

Following Meisner et al.,[20] we simplify the generating model to work with a single genetic factor $G = X\beta$ and an environmental interaction with the genetic factor $(G \circ E)\delta$ rather than SNP-specific interaction terms $(X \circ E)\Delta$ as in Equation 1. This is equivalent to an SNP-level model where each SNP effect has a "coordinated" interaction with E.[14,21]

$$y = C\alpha + G\beta_G + E\gamma + (G \circ E)\delta + \epsilon \qquad \text{(Equation 2)}$$

In Equation 2, β_G is a scaling factor for polygenic factor $G = X\beta$. This is 1 for the generating model with $G = X\beta$, but in an estimation model corresponding to Equation 2 where an estimated PGS is used to approximate G, $\widehat{\beta}_G$ will be less than one. With a vector G instead of a matrix X, there is now simply a scalar $G \times E$ effect size δ.

For each simulation, $n = 2{,}000$ study participants were generated. The matrix C was set to hold ten standardized normally distributed covariates, and their effect sizes were set to the same value α such that the fraction of the variance of y explained by C was 30%.

The genotype matrix X was set to have 2,000 independent additively coded SNPs. Minor alleles for each SNP were chosen uniformly in the range [0.05, 0.5]. Each genotype was centered and scaled to have a mean of zero and a variance of one. A sparse genetic model was used with 10% of the SNPs in X having normally distributed effect sizes $\beta \sim \mathcal{N}(0, \sigma^2_g / m_c)$, with $\sigma^2_g = 0.3$ and the number of SNPs with non-zero effect sizes $m_c = 200$. The remaining SNPs had an effect size of zero. The total genetic effect G was calculated by multiplying X by the vector of effect sizes.

Table 1. Non-homogeneous quantile-specific effect sizes for regression of traits on PGSs

Trait	Trait code	$\widehat{\beta}_{OLS}$	Min $\widehat{\beta}_\tau$	Max $\widehat{\beta}_\tau$	Quantile with min $\widehat{\beta}_\tau$	Quantile with max $\widehat{\beta}_\tau$	Linear trend *p* value	Quadratic trend *p* value	No. of observations
Age at menopause	AAM	0.23	0.17	0.33	0.95	0.15	8.0E−39	1.4E−06	30,638
Apolipoprotein A1	APOEA	0.31	0.23	0.44	0.05	0.95	1.5E−107	3.6E−08	90,591
Apolipoprotein B	APOEB	0.33	0.22	0.46	0.05	0.95	1.0E−217	0.74	98,916
Body mass index	BMI	0.35	0.18	0.60	0.05	0.95	<1E−300	9.2E−36	103,850
Calcium	CAL	0.25	0.24	0.27	0.05	0.95	3.9E−04	0.76	91,113
Docosahexaenoic acid	DOA	0.22	0.19	0.29	0.1	0.95	6.6E−08	0.20	24,860
Estimated bone mineral density	EBMDT	0.13	0.03	0.26	0.05	0.95	1.4E−08	0.87	3,569
Glomerular filtration rate (creatinine)	EGCR	−0.20	−0.24	−0.16	0.95	0.05	2.9E−58	0.87	99,439
Glomerular filtration rate (cystatin)	EGCY	−0.27	−0.35	−0.21	0.95	0.05	3.3E−91	1.0E−03	99,484
Glycated hemoglobin	HBA1C	0.24	0.20	0.44	0.05	0.95	1.6E−05	2.3E−06	98,152
Height	HEIGHT	0.55	0.51	0.57	0.05	0.85	3.7E−17	9.7E−05	103,968
High-density lipoprotein cholesterol	HDL	0.40	0.28	0.56	0.05	0.95	1.6E−256	5.0E−11	91,089
Intraocular pressure	IOP	0.18	0.14	0.21	0.1	0.95	8.6E−13	7.1E−02	29,386
Low-density lipoprotein cholesterol	LDL	0.31	0.16	0.44	0.05	0.95	<1E−300	3.0E−07	99,296
Omega-3 fatty acids	OTFA	0.27	0.20	0.37	0.05	0.95	7.2E−34	0.62	24,860
Omega-6 fatty acids	OSFA	0.25	0.14	0.38	0.05	0.95	8.4E−36	0.85	24,860
Phosphatidylcholines	PDCL	0.29	0.21	0.39	0.05	0.95	6.5E−26	0.97	24,860
Phosphoglycerides	PHG	0.27	0.19	0.38	0.05	0.95	5.8E−26	0.66	24,860
Polyunsaturated fatty acids	PFA	0.26	0.17	0.37	0.05	0.95	7.1E−31	0.51	24,860
Remnant cholesterol	RMNC	0.24	0.15	0.33	0.05	0.95	3.6E−42	6.8E−02	24,875
Resting heart rate	RHR	0.25	0.20	0.31	0.05	0.95	9.3E−48	0.17	98,783
Sphingomyelins	SGM	0.27	0.20	0.37	0.05	0.95	4.9E−18	0.99	24,860
Total cholesterol	TCH	0.25	0.15	0.33	0.05	0.95	1.6E−27	3.8E−02	24,875
Total fatty acids	TFA	0.25	0.15	0.39	0.05	0.95	3.9E−55	1.6E−02	24,860
Total triglycerides	TTG	0.27	0.10	0.45	0.05	0.95	2.8E−150	3.9E−03	24,875

The exposure E was dichotomous with probability of exposure PrE. A vector of individual gene-by-environment factors $G \circ E$ was formed by element-wise multiplication of G and E.

Different values for the model parameters were used to generate data for the various simulation analyses: genetic variance σ_g^2, the probability of exposure PrE, the environmental main effect γ, the $G \times E$ effect size δ, and the noise variance σ_ϵ^2.

For each simulation run, two datasets were generated with the same simulation parameters, as well as the same minor-allele frequencies for X and the same genetic effect sizes. For one of the datasets, genetic association tests were run, with adjustment for the covariates in C. PGS weights were set to be the regression estimates for the SNP effects with p values below 1E−04. PGSs were estimated for the second dataset using these weights. The plots and regression analyses proceeded in this second dataset.

Results

Methods overview

We developed a method to distinguish the phenotype-PGS pairs with substantial heterogeneity across the phenotypic range. We use QR to estimate models for a set of quantiles of the phenotype distribution as linear functions of the value of a PGS. For all analyses presented here, we use 19 quantiles τ, evenly spaced in the unit interval (0.05, 0.10, …, 0.95). We consider the deviations between the quantile-specific effect sizes $\widehat{\beta}_\tau$ and the OLS effect size estimate $\widehat{\beta}_{\text{OLS}}$ from regression of the phenotype on PGS. We seek not just statistically significant deviations but rather qualitatively meaningful deviations that impact the practical use of PGSs. We define these using a non-equivalence test, with a null hypothesis that quantile-specific effect sizes are no more than λ-fold different from the effect estimated by OLS. This is important because even small fluctuations in quantile-specific effects can be statistically significant at large sample sizes when the null hypothesis is that all $\widehat{\beta}_\tau$ are identical or equal to $\widehat{\beta}_{\text{OLS}}$. To account for the uncertainty in both the QR and OLS effect-size estimates, we calculate the ratio $\widehat{\beta}_\tau/\widehat{\beta}_{\text{OLS}}$ for each quantile τ and estimate the ratios' standard errors using an m-of-n bootstrap. For a level $\alpha = 0.10$ non-equivalence test with a λ-fold equivalence region, we reject the null that $\widehat{\beta}_\tau$ and $\widehat{\beta}_{\text{OLS}}$ are equivalent when the $(1 - \alpha/2)$% confidence intervals for the ratios $\widehat{\beta}_\tau/\widehat{\beta}_{\text{OLS}}$ are completely outside the $(1/\lambda, \lambda)$ equivalence band.

Figures 1 and 2 provide an overview of the method. In Figure 1, scatterplots of trait versus PGS are shown on the left for height, BMI, and asthma. As the first step in the analysis, the residual trait and PGS are generated as the residuals from the linear regression of the trait and PGS, respectively, on additional covariates (age, age^2, sex, age*sex, and ten genetic principal components). The residual trait and PGS are rescaled to have a mean of zero and a variance of one so that the OLS linear regression coefficient for the residual trait on the residual PGS is the same as the correlation, and this can be squared to give a predictive R^2. Scatterplots of residuals are shown on the right of Figure 1, along with three lines representing QR linear models fit at the 95th, 50th, and 5th quantiles of the residual trait distribution. The nearly parallel lines for the QR linear models for height illustrate the homogeneous predictive value of the height PGS for height across the phenotypic range, while the non-parallel lines for BMI illustrate the non-uniform predictive value of the PGS across the phenotypic range.

The plots and QR linear models for asthma in the bottom images of Figure 1 illustrate how the analyses we describe below are not appropriate for categorical traits or traits with well-separated modes. The plotted lines from the QR linear models for asthma are essentially flat, with zero slopes, because the three quantiles of the residual asthma distribution are in two distinct modes across the PGS distribution.

In Figure 2, the quantile-specific effect size estimates, $\widehat{\beta}_\tau$, and their 95% confidence intervals are plotted against quantile τ. The OLS effect-size estimates for each trait, $\widehat{\beta}_{\text{OLS}}$, are shown as gray horizontal lines with dashed lines for their 95% confidence intervals. BMI and height show qualitatively distinct patterns. For height, the deviation of $\widehat{\beta}_\tau$ from $\widehat{\beta}_{\text{OLS}}$ is always small, 0.04 or less in units of standard deviations of residual height. For BMI, the deviation is striking: the PGS has much larger effect sizes in the highest BMI quantiles. The results for asthma again show that this approach is not informative for categorical traits or those with well-separated modes. For asthma, $\widehat{\beta}_\tau$ is near zero except at quantiles τ around 1-case prevalence, where the quantile of residual asthma moves from one mode to the other at different levels of the PGS.

Notwithstanding this qualitative difference across continuous traits such as height and BMI in patterns of $\widehat{\beta}_\tau$ versus τ, trend tests are highly significant for all traits analyzed (Table 1). Our new test for non-equivalence resolves this problem of flagging all traits as having a variable predictive value across the phenotypic range. On the right side of Figure 2, ratios $\widehat{\beta}_\tau/\widehat{\beta}_{\text{OLS}}$ are plotted against τ with their 95% confidence intervals. Applying the non-equivalence test with $\lambda = 1.2$ corresponds to checking that the confidence intervals for the ratios are completely below $1/\lambda = 0.833$ or above $\lambda = 1.2$; the limits of the equivalence band are illustrated with horizontal lines on the right side of Figure 2. None of the deviations for height are significant, as all estimated ratios $\widehat{\beta}_\tau/\widehat{\beta}_{\text{OLS}}$ have confidence intervals that intersect the equivalence band (1/1.2, 1.2). By contrast, quantile-specific effect sizes are substantially different than the mean effect size for BMI in the quantiles less than 0.4 and greater than 0.8, with much larger effect sizes in the highest BMI quantiles. This is a clear demonstration of the importance of emphasizing qualitative differences, especially in massive datasets that have high power to detect small quantitative fluctuations.

We analyzed the 25 continuous phenotypes with standardized polygenic risk scores (PRSs) released by UKBB using the procedure illustrated in Figures 1 and 2, with the

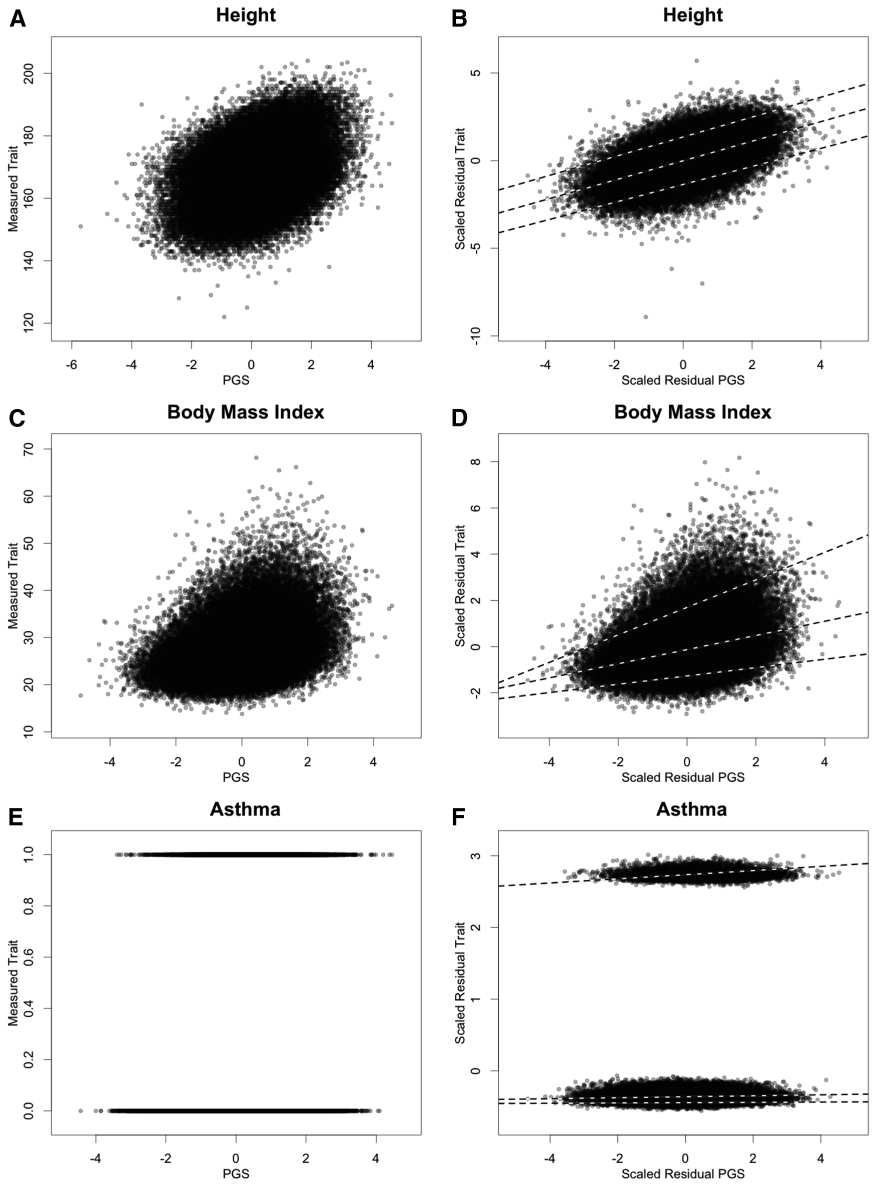

Figure 1. Residual quantile regression of trait versus PGS for height, BMI, and asthma
In (A), (C), and (E), scatterplots of height, BMI, and asthma versus their polygenic scores (PGSs) are shown. For each trait or PGS, residuals from linear regression on sex, age, age^2, sex*age, and ten genetic PCs were generated and rescaled to have a mean of 0 and a variance of 1. Residual trait and residual PGSs are plotted in (B), (D), and (F). The dashed lines represent quantile regression linear models for the 95th, 50th, and 5th percentiles of the conditional distributions.

results shown in Table 1. The OLS estimates $\widehat{\beta}_{\text{OLS}}$ are shown alongside the minimum and maximum quantile-specific estimates. Note that the extreme values of $\widehat{\beta}_\tau$ tend to be at the highest and lowest quantiles, $\tau = 0.05$ or 0.95. As expected, the linear trend tests for $\widehat{\beta}_\tau$ versus τ indicate significant trends for all 25 traits, with minimum p values below 1E−300 and the vast majority below 1E−07. The quadratic trend test p values are from likelihood ratio tests comparing a quadratic fit and a linear fit to $\widehat{\beta}_\tau$ versus τ for each trait. Only seven of the 25 traits have significant improvements in likelihood from the quadratic term, with p values below 1E−03. These significance tests establish that PGS predictive values are universally heterogeneous across the phenotypic range but do not directly inform the qualitative significance of this heterogeneity. Figures in the supplemental information correspond to plots in Figures 1 and 2, showing $\widehat{\beta}_\tau$ and $\widehat{\beta}_{\text{OLS}}$, for each trait in Table 1.

The non-equivalence tests are more informative than the trend tests. Table 2 shows the numbers of quantiles (of 19 evaluated) for each trait that are flagged as having non-homogeneous associations with PGS at different values of λ. 60% of traits (15 of 25) show substantial differences in regression coefficients, with at least two of the 19 quantile-specific regression coefficients over 1.2-fold different from the OLS estimate, but there are striking differences in their patterns of heterogeneity across traits.

The differences in the number of outlying quantiles across the 25 phenotypes emphasize the ubiquity of the non-uniform PGS predictive value. This is best seen by examining plots of the quantile-specific effect sizes $\widehat{\beta}_\tau$ versus quantile, as shown in Figures 1 and 2. These plots reveal apparent linear trends or curvature in the relationship between effect sizes and quantiles, the statistical significance of which is quantified by p values for linear and quadratic trend fits (as shown in Table 1). However, while both tests indicate a significant deviation from homogeneity of phenotype-PGS associations, they fail to adequately capture the magnitude and qualitative differences in the distributions of the effect sizes. Effect sizes for the slope of the linear trend fit or the linear and quadratic terms of the quadratic trend fit may suggest the magnitude of the heterogeneity of the PGS predictive value, if the $\widehat{\beta}_\tau$ values are well approximated by linear or quadratic fits, but as we see with the examples in Figure 2, outlying quantiles are poorly fit by these simple models. Figure 3 illustrates the qualitative patterns that the distribution of effect sizes may take. We next explored, by simulation, the likely origins of these departures from homogeneity of the PGS predictive value across the trait distribution.

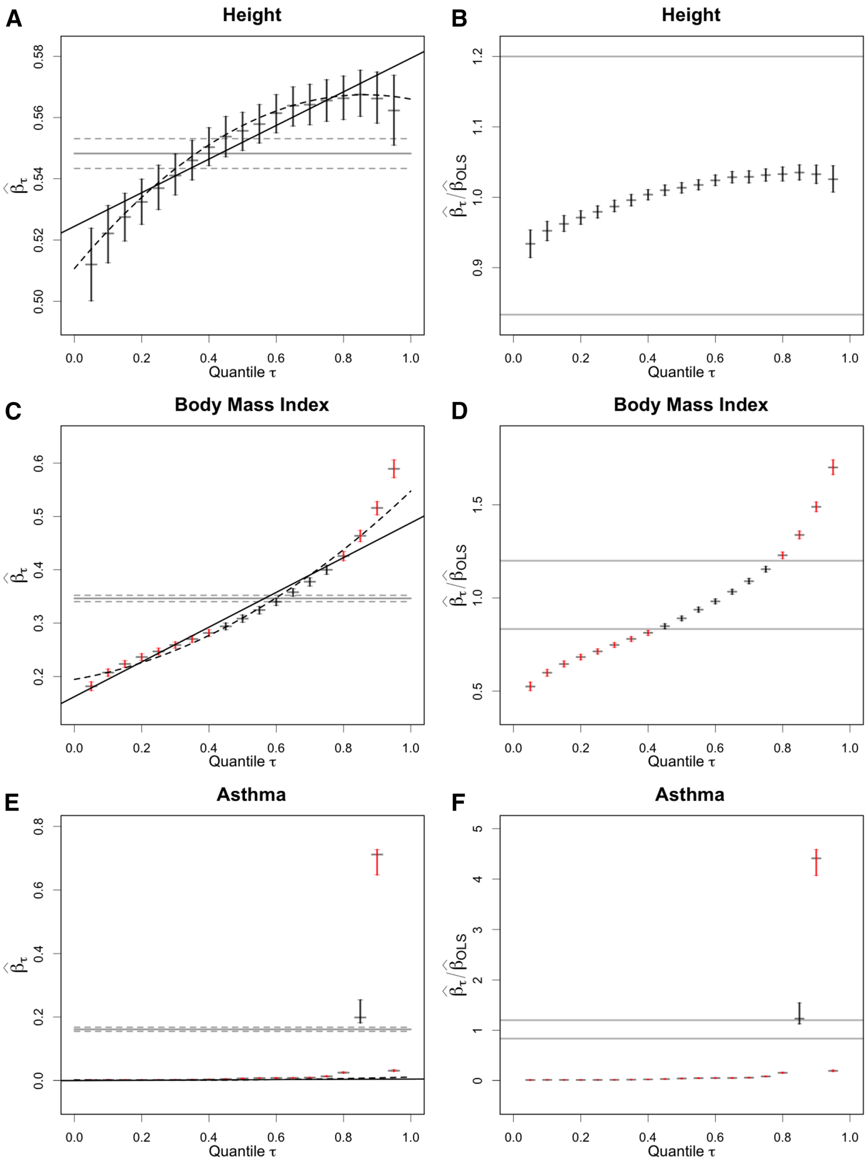

Figure 2. Quantile-specific PGS regression coefficients for height, BMI, and asthma
(A), (C), and (E) show the estimated quantile-specific effect sizes $\widehat{\beta}_\tau$ for the regression of residual BMI, height, or asthma on the corresponding residual polygenic scores. Estimates of $\widehat{\beta}_\tau$ are shown with their 95% confidence intervals for 19 evenly spaced quantiles from 0.05 to 0.95. The linear and quadratic trend lines are shown with solid and dashed lines, respectively. The average relationship between residual phenotype and polygenic scores from OLS, $\widehat{\beta}_{\mathrm{OLS}}$, is shown as a horizontal gray line, with dashed lines indicating 95% confidence intervals. All units are scaled for $\widehat{\beta}_{\mathrm{OLS}}$ to be the correlation. (B), (D), and (F) show the ratios $\widehat{\beta}_\tau/\widehat{\beta}_{\mathrm{OLS}}$ with their 95% confidence intervals. For these images, the horizontal lines at 1/1.2 and 1.2 show the boundaries of a user-specified practical equivalence band $(1/\lambda,\ \lambda)$. For ratios $\widehat{\beta}_\tau/\widehat{\beta}_{\mathrm{OLS}}$ whose 95% confidence intervals do not extend into the equivalence bands, vertical bars indicating their confidence intervals are colored red. Equivalently, ratios with red-colored confidence intervals reject a null hypothesis of $\widehat{\beta}_\tau/\widehat{\beta}_{\mathrm{OLS}}$ in $(1/\lambda, \lambda)$ by a non-equivalence test at significance level $\alpha = 0.10$. For ratios with red-colored confidence intervals, the corresponding confidence intervals for $\widehat{\beta}_\tau$ on the left side of the figure (A, C, and E) are also colored red.

Simulations

We use simulations to explore mechanisms that can generate the observed patterns for quantile-specific effect sizes $\widehat{\beta}_\tau$ versus quantile τ. Analyses illustrated in Figures 4 and 5 provide a framework for interpreting the patterns in terms of environmental exposure, genetic susceptibility, and interactions between the two. For each simulation, a continuous trait was generated with contributions from 200 causal SNPs, ten Gaussian covariates, and a dichotomous exposure. In the scatterplots, exposed observations are represented by blue dots and unexposed observations by black dots. Across the simulations, the proportions of the observations having the exposure, environmental main effect, and $G \times E$ interactions were varied. 40,000 observations were generated and split into two sets of 20,000. In one, a GWAS was run, and a PGS was developed. The PGS was calculated for the second half of the simulated data and analyzed using QR and non-equivalence tests.

The data-generating model specified in the material and methods sections was run with parameters β, γ, δ, and PrE, indicated in the legends for Figures 4 and 5, where β is a scaling factor for the heritability or predictive value of the PGS, γ is the E main effect, δ is a scaling factor for the $G \times E$ interaction (the extra trait heritability among the exposed), and PrE is the proportion of observations that are exposed.

The first four images in Figure 4 illustrate the patterns expected from two scenarios where we find homogeneity (constancy) of the estimates $\widehat{\beta}_\tau$. The images are presented in pairs: first a scatterplot of residual traits versus residual PGSs and then $\widehat{\beta}_\tau$ versus quantile τ. The first pair, Figures 4A and 4B, illustrates a simulation where there is no environmental effect, and the homogeneity of $\widehat{\beta}_\tau$ is evident (Figure 4B). Figures 4C and 4D show results from a simulation where there is an environmental effect but no $G \times E$ interaction, again resulting in a homogeneous distribution for $\widehat{\beta}_\tau$ (Figure 4D). These two patterns contrast with those that illustrate non-homogeneity. Non-homogeneity arises from two sources in the simulations: from a $G \times E$ interaction and from deviations from unimodality in the phenotypic distribution (of which one extreme and

Table 2. The number of quantiles with regression coefficients substantially different than the OLS estimate for regression of traits on PGSs

Trait	Trait code	$\lambda = 1.05$	$\lambda = 1.1$	$\lambda = 1.2$	$\lambda = 1.5$	$\lambda = 2$
Age at menopause	AAM	12	10	7	0	0
Apolipoprotein A1	APOEA	14	10	4	0	0
Apolipoprotein B	APOEB	14	12	7	0	0
Body mass index	BMI	17	15	12	4	0
Calcium	CAL	0	0	0	0	0
Docosahexaenoic acid	DOA	8	1	0	0	0
Estimated bone mineral density	EBMDT	9	8	8	3	1
Glomerular filtration rate (creatinine)	EGCR	11	5	1	0	0
Glomerular filtration rate (cystatin)	EGCY	13	8	2	0	0
Glycated hemoglobin	HBA1C	17	15	1	1	0
Height	HEIGHT	0	0	0	0	0
High-density lipoprotein cholesterol	HDL	15	12	7	0	0
Intraocular pressure	IOP	9	3	0	0	0
Low-density lipoprotein cholesterol	LDL	16	14	10	2	0
Omega-3 fatty acids	OTFA	12	9	4	0	0
Omega-6 fatty acids	OSFA	13	11	6	1	0
Phosphatidylcholines	PDCL	12	7	3	0	0
Phosphoglycerides	PHG	12	7	3	0	0
Polyunsaturated fatty acids	PFA	13	9	5	0	0
Remnant cholesterol	RMNC	14	11	7	1	0
Resting heart rate	RHR	11	8	1	0	0
Sphingomyelins	SGM	9	6	2	0	0
Total cholesterol	TCH	13	9	2	0	0
Total fatty acids	TFA	15	12	10	2	0
Total triglycerides	TTG	17	15	13	5	1

Quantile regression linear models were used to estimate changes in residual phenotype with changes in residual PGS at 19 evenly spaced quantiles (0.05, 0.10, …, 0.95). Ratios of quantile-specific effect sizes to effect sizes from OLS models were calculated and their distributions were estimated using an *m*-of-*n* bootstrap. Ratios whose 95% confidence intervals were completely outside the band from $[1/\lambda, \lambda]$ indicate that the quantile-specific effect sizes are not equivalent to the OLS effect size. The numbers of quantiles, out of 19, where QR effect sizes are not equivalent to OLS estimates are shown for different values of λ.

common example is a case-control cohort). Figures 4E–4N show different simulations with $G \times E$ interactions. The first (Figures 4E and 4F) is a simulation with no environmental main effect. This is followed by three simulations with both an E main effect and $G \times E$, where the positive signs of both γ and δ indicate increasing trait values in the exposed (Figures 4G–4L). Figures 4M and 4N illustrate data with an E main effect and a $G \times E$ interaction, but here, the environmental effect is in the direction opposite to that of the interaction. Finally, Figures 4O and 4P show the results of applying the QR and non-equivalence tests to case-control data generated by treating a simulated continuous trait in Figures 4G and 4H as a liability and labeling observations with the top 30% of liabilities as cases.

In the scenario with a $G \times E$ interaction but no E main effect (Figures 4E and 4F), the $G \times E$ interaction makes the effective heritability of the trait or the predictive value of the PGS stronger in the exposed than in the unexposed. The mean residual trait values are the same (zero) in both the exposed and unexposed groups, but because the PGS is predictive of trait values, observations with low values of both the trait and the PGS or high values for both are enriched for exposed observations. When quantile-specific trend lines are estimated for high quantiles ($\tau = 0.95$, top line in Figure 4E), that line goes from predominantly unexposed observations at low residual PGS values to predominantly exposed observations at high residual PGS values. The result is a large value of $\widehat{\beta}_\tau$ relative to $\widehat{\beta}_{\mathrm{OLS}}$. There are similarly large estimates of $\widehat{\beta}_\tau$ at low quintiles ($\tau = 0.05$, bottom line in Figure 4E) but lower estimates of $\widehat{\beta}_\tau$ at intermediate quintiles ($\tau = 0.50$, middle line in Figure 4E). The result is the "smile" pattern shown in Figure 4F. With the data generation parameters used for this simulation and the non-equivalence tests run at level $\alpha = 0.10$ and $\lambda = 1.1$, none of the quantile-specific effect sizes $\widehat{\beta}_\tau$ were significantly non-equivalent to $\widehat{\beta}_{\mathrm{OLS}}$, but in

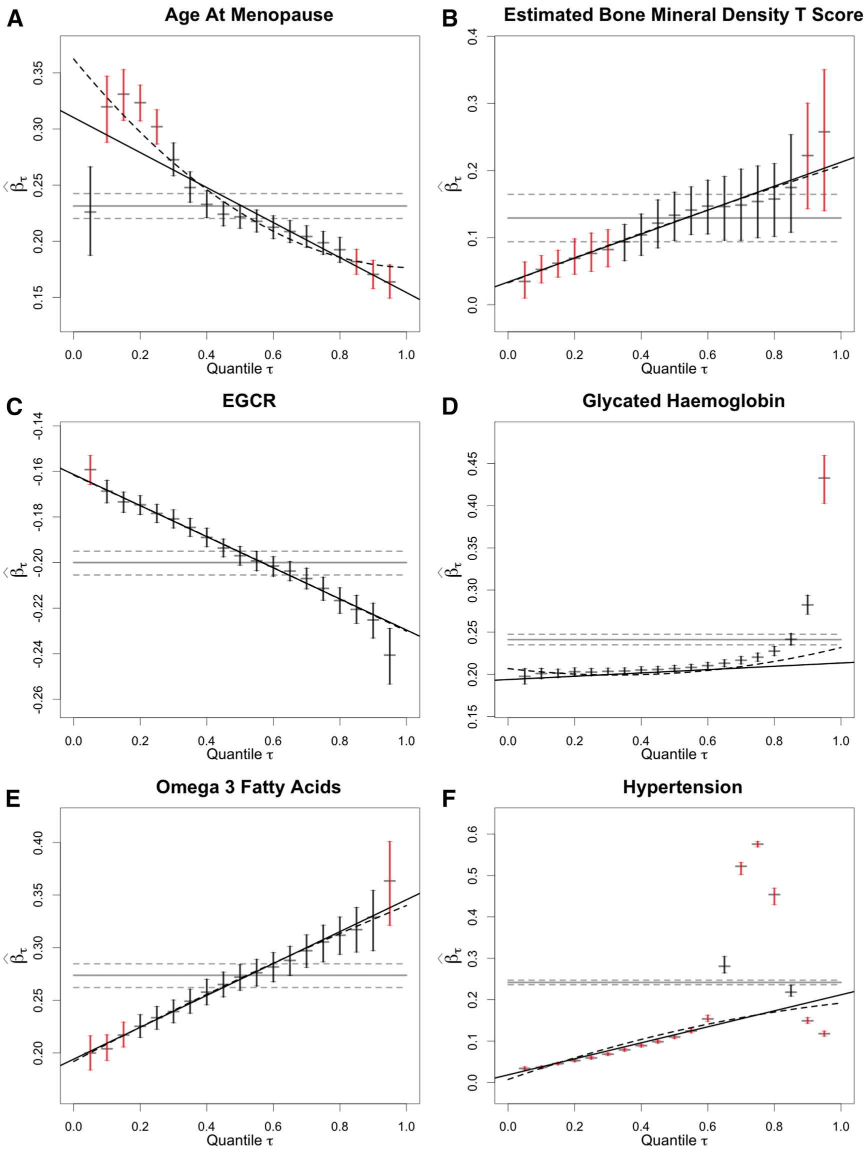

Figure 3. Diverse patterns for $\widehat{\beta}_\tau$ versus τ

Plots of $\widehat{\beta}_\tau$ versus τ. Vertical bars for $\widehat{\beta}_\tau$ are 95% confidence intervals. Vertical bars that are colored red indicate that the ratio of $\widehat{\beta}_\tau$ to $\widehat{\beta}_{\mathrm{OLS}}$ is outside of the interval $(1/\lambda, \lambda)$ with $\lambda = 1.2$ at a 90% confidence level or by a non-equivalence test at significance level $\alpha = 0.10$.
(A) Age at menopause.
(B) Estimated bone mineral density.
(C) Glomerular filtration rate, creatinine (EGCR).
(D) Glycated hemoglobin (HbA1c).
(E) Omega-3 fatty acids.
(F) Hypertension, a case-control trait.

Figure 4E, we can see the smile pattern, with $\widehat{\beta}_\tau$ having confidence intervals outside of the confidence interval for $\widehat{\beta}_{\mathrm{OLS}}$ for τ in (0.05, 0.45–0.60, and 0.95).

Suppose one of the patterns of non-homogeneity is seen; how can its origin be determined? Figures 5A and 5B illustrate a pattern that might be found, which, from its resemblance to Figures 4G and 4H, might be caused by $G \times E$ (we assume that the investigator has excluded a deviation from unimodal trait distribution). The line in Figure 5B crosses the OLS estimate at about 0.75, suggesting that whatever the environmental exposure is, it divides the population into a 75/25 split. Let's say the investigator guesses the exposure is treatment with statin drugs (which about 25% of the sample take). To test this hypothesis, statins are included as an adjustment covariate in the original GWAS to generate a PGS for use in QR and non-equivalence tests. Figures 5C and 5D show the result. The analysis does not remove the interaction, but it shifts the means of the two groups defined by the dichotomous variable. From Figures 4E and 4F, we expect $G \times E$ with no main E effect to have this smile, which is the result shown in Figure 5D. As confirmation, the sample is stratified by statin exposure and the analyses run again for the two strata. Figures 5F and 5H show that both strata are homogeneous and that the predictive scores and heritability are different across the strata, confirming the relevant exposure has been identified. The confirmatory analysis does not require access to the genetic source data and development of a new PGS, as illustrated in Figure S34.

Discussion

We have shown in this paper that non-equivalence tests for quantile-specific PGS effect sizes provide a way of detecting substantial deviations from homogeneity in predictive value of PGSs across the range of a phenotype's distribution.

Our approach for dealing with adjustment covariates such as age and genetic principal components in the QR analysis is to project these covariates out of both the trait of interest and the PGS using linear regression and then perform univariate QR on the residual trait and residual PGS. This approach allows QR to scale to analyses of biobanks and aids the interpretability of the quantile-specific effect sizes for the association of PGS with the trait. A similar residualization approach has recently been proposed in the QR field[22] to improve the interpretability of a single predictor used in the QR analysis. An individual at a particular quantile of the trait distribution in the trait ~ PGS analysis, say the 95th percentile, would generally be at a different position in the trait distribution for analyses with different covariates, say the 45th in a trait ~ age analysis. This

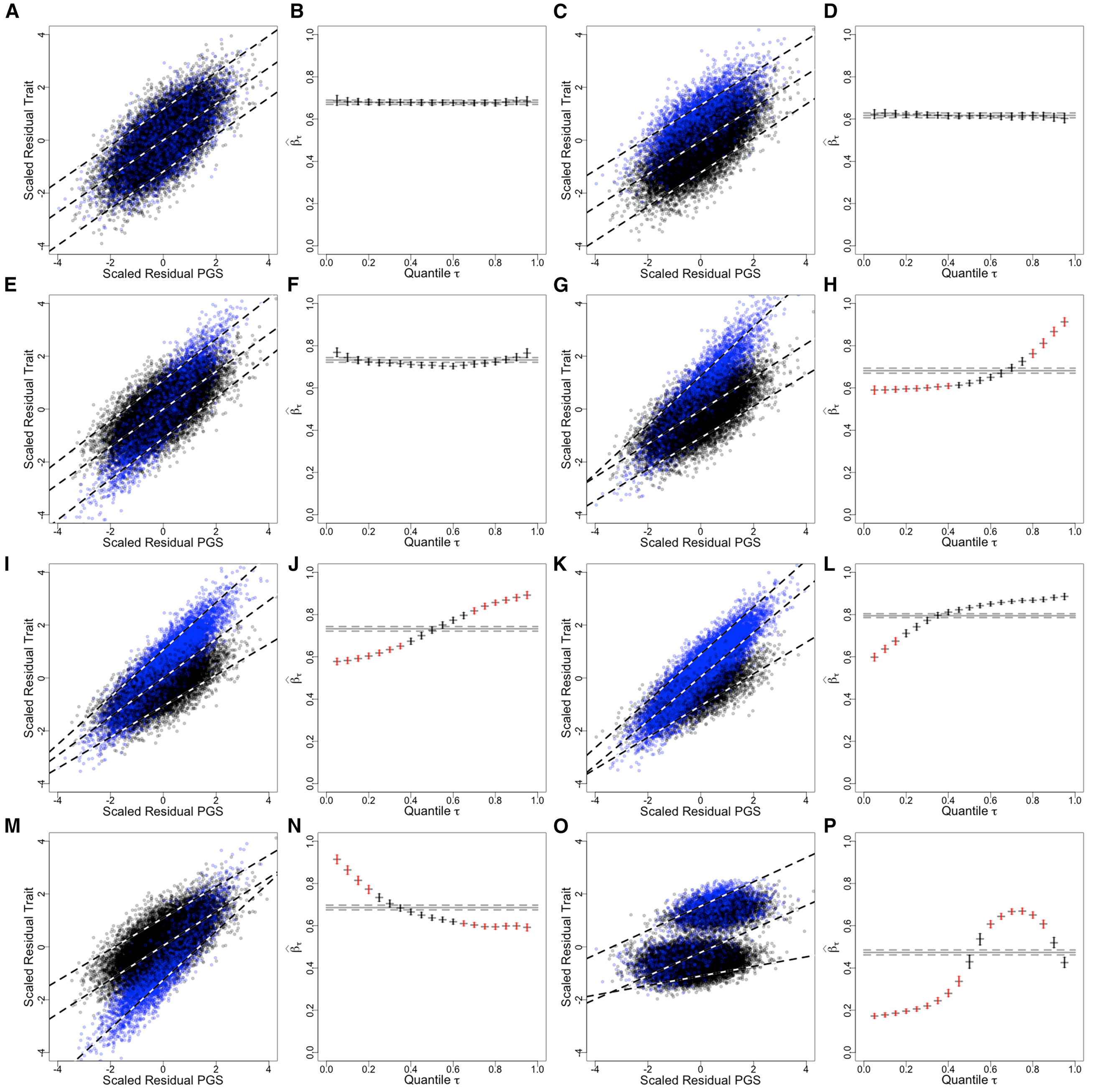

Figure 4. Homogeneous and heterogeneous quantile-specific effect sizes

A pair of plots is presented for each simulation: a scatterplot of residual trait versus residual PGS on the left and a plot of quantile-specific effect size estimates versus quantile on the right. In the scatterplots, blue dots correspond to exposed observations ($E = 1$) and black dots to non-exposed observations ($E = 0$). Vertical bars around $\widehat{\beta}_\tau$ estimates represent 95% confidence intervals. Bars that are colored red indicate that the ratio of $\widehat{\beta}_\tau$ to $\widehat{\beta}_{\mathrm{OLS}}$ is outside of the interval $(1/\lambda, \lambda)$ with $\lambda = 1.2$, rejecting the null of a non-equivalence test at significance level $\alpha = 0.10$.

(A and B) Homogeneous model where there is no $G \times E$ interaction and no E main effect. $\beta = 1, \gamma = 0, \delta = 0$, and $PrE = 0.25$. All the quantile-specific linear trend lines for residual traits versus residual PGSs are parallel, with near identical values for $\widehat{\beta}_\tau$. These values closely match $\widehat{\beta}_{\mathrm{OLS}}$, and the ratios $\widehat{\beta}_\tau/\widehat{\beta}_{\mathrm{OLS}}$ are close to one.

(C and D) Model with an E main effect but no $G \times E$ interaction. $\beta = 1, \gamma = 1, \delta = 0$, and $PrE = 0.25$. There is a shift in the residual trait values with exposed observations having higher values, but quantile-specific linear trend lines for residual traits versus residual PGSs are still parallel, with near identical values for $\widehat{\beta}_\tau$.

(E and F) Model with a $G \times E$ interaction but no E main effect. $\beta = 1, \gamma = 0, \delta = 1$, and $PrE = 0.25$.

(G and H) Model with both a $G \times E$ interaction and an E main effect. $\beta = 1, \gamma = 1, \delta = 1$, and $PrE = 0.25$.

(I and J) Model with both a $G \times E$ interaction and an E main effect. $\beta = 1, \gamma = 1, \delta = 1$, and $PrE = 0.50$.

(K and L) Model with both a $G \times E$ interaction and an E main effect. $\beta = 1, \gamma = 1, \delta = 1$, and $PrE = 0.75$.

(legend continued on next page)

has led to the ongoing development of different methods for QR with the goal of making interpretable quantile-specific regression coefficients when there are additional adjustment covariates.

Applying QR to 25 phenotypes in the UKBB with $\lambda = 1.2$ revealed that 70% of traits have substantial differences in quantile-specific regression coefficients of the trait by the corresponding PGS. Within these heterogeneous effect-size distributions, there is at least one quantile for which the quantile-specific effect size is above or below the average effect size, estimated by regression.

The patterns of deviations can be explained by the presence of unacknowledged gene-by-environment interactions. In the UKBB dataset, we found several patterns of deviation from homogeneity, the origins of which we investigated through extensive simulation. Gene-by-environment interactions can explain them all. Our results show that non-homogeneity in the prediction of PGS suggests the presence of an un-modeled $G \times E$ interaction and that the shape of the relationship between quantile and quantile-specific effect size can be used to estimate the prevalence of the exposure to E (without knowledge of the exposure). The quantile where the minimum or maximum occurs corresponds to the probability of a binary environmental exposure or one minus the probability for exposures with positive or negative environmental main effects.

Previous studies[18,23] have used conditional QR (CQR) to estimate quantile-specific linear models for phenotype as a function of PGS and then run trend tests to illustrate the non-homogeneity of the quantile-specific effect sizes. In addition to showing statistically significant results for very small trends, studies[18,23] report that such trend tests are mis-calibrated and give increasingly inflated false discovery rates when larger numbers of quantiles are estimated for the trend tests.

The increasing interest in PGSs as predictive tools for many applications requires increased attention to the factors that confound their interpretation. Our findings add to this discussion by showing that a PGS's predictive accuracy depends on the quantile of the phenotypic distribution to which the PGS is being applied. This work has the following implications: (1) population strata where PGSs have either a weaker or stronger predictive value than the assumed value when ignoring heterogeneity may be given ineffective or counter-productive interventions when treatment policies are based on PGSs. (2) Treatment policies based on PGSs are intrinsically biased if the PGSs have non-uniform predictability across population strata, and the use of such policies in this situation can lead to health disparities. (3) The non-uniform predictive value of PGSs is consistent with an important source of heterogeneity within the population. Possible sources of heterogeneity include an environmental factor that impacts the phenotype but with only a fraction of the population exposed, different subtypes of the underlying biological trait that affects the measured phenotype, important $G \times E$ interactions, or perhaps $G \times G$ interactions.

Some of the patterns we see are expected, notably the impact of the ties on the analysis of case-control phenotypes, such as asthma. The pattern of quantile-specific effects found in bone mineral density (BMD) can be explained as the age- and sex-specific consequences of estrogen loss following menopause: a non-genetic effect that results in the extremes of BMD being enriched for young females at the high BMD end and older females being enriched at the low BMD end. Consequently, the quantile-specific associations between BMD and its PGS will be relatively weak at the lowest and highest quantiles, where the age-specific strata are over-represented. At intermediate quantiles, the PGS is more predictive, resulting in a "frown," with greater predictive value for the PGS at intermediate quantiles. While we use the term "environment" loosely, these effects need not be strictly environmental: for example, we may explain the pattern for changes in glomerular filtration rate, creatinine method (EGCR) in Figure 3C as due to the interaction between age and the PRS, where the impact of age is considered as dichotomous, affecting ages below and above a certain threshold, such that the younger subpopulation typically has higher filtration rates that have lower correlation to PGS than the older population. To visualize this, consider Figures 4M and 4N, with black dots to represent the younger population and blue for the older population. If the group sizes were more evenly balanced for the simulation in Figure 4N, the image would closely match the pattern seen in Figure 3C. Other deviations can be best interpreted as due to the presence of a phenotypic subtype. It should be noted that in this case, the presence of a subtype is formally no different from the presence of exposure, where an exposure indicator equates to a subtype indicator. An example is shown in Figure 3D for HbA1c, where the extreme deviation for the higher quantiles is attributable to the presence of a group of subjects with obesity and/or diabetes. Finally, the distribution of some quantile effects reveals interactions with a factor that has yet to be determined (we place our observations for omega-3 fatty acids in this category). What should investigators do if they detect non-homogeneity in their analyses? We

(M and N) Model with both a $G \times E$ interaction and an E main effect but with the main effect in the opposite direction as the interaction effect. $\beta = 1, \gamma = 1, \delta = -1$, and $PrE = 0.25$.

(O and P) For these simulations, the continuous trait in (G) and (H) was considered to be a liability for a case-control trait. Observations in the top 30% for this liability were designated cases, with the trait coded as a 1, with trait values for the remainder of observations coded as 0. $\beta = 1, \gamma = 1, \delta = 1$, and $PrE = 0.25$.

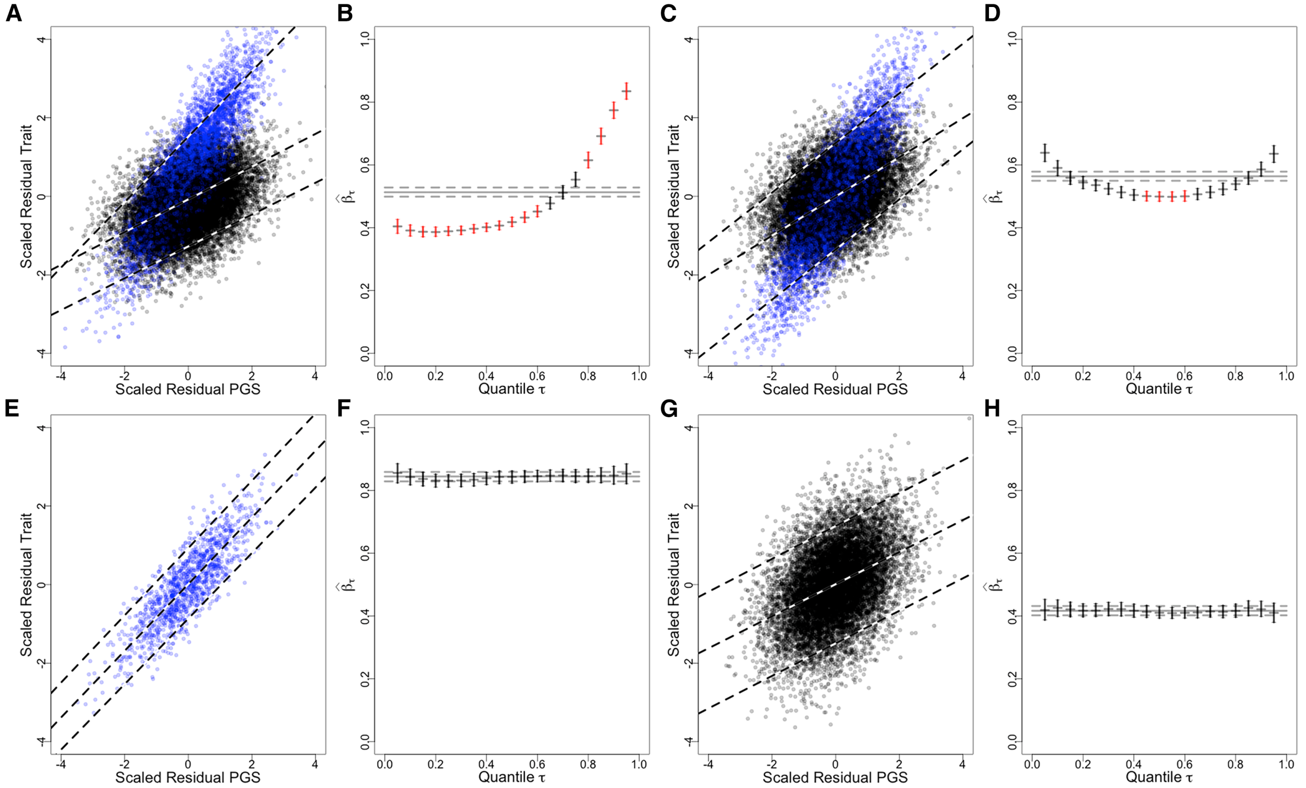

Figure 5. Adjustment and stratification by exposure

The impact of adjustment and stratification by exposure on quantile regression and non-equivalence tests. For all scenarios illustrated, the same dataset was used—one having both a $G \times E$ interaction and an E main effect, with 25% of the observations having been exposed: $\beta = 0.5, \gamma = 1, \delta = 1$, and $PrE = 0.25$. For each analysis, there are two paired plots: first a scatterplot of residual traits versus residual PGSs (A, C, E, and G) and then plots of quantile-specific effect size estimates versus quantile (B, D, F, and H). In the scatterplots, blue dots correspond to exposed observations ($E = 1$) and black dots to non-exposed observations ($E = 0$). Vertical bars around $\widehat{\beta}_\tau$ estimates represent 95% confidence intervals. Bars that are colored red indicate that the ratio of $\widehat{\beta}_\tau$ to $\widehat{\beta}_{\mathrm{OLS}}$ is outside of the interval $(1/\lambda, \lambda)$ with $\lambda = 1.2$, rejecting the null of a non-equivalence test at significance level $\alpha = 0.10$.
(A and B) The dataset was analyzed without accounting for the exposure E.
(C and D) An indicator variable for the exposure E was included as an adjustment covariate both for the GWAS used in PGS development and in the residualization step.
(E and F) The observations were stratified into exposed and non-exposed groups. Only the exposed observations were used for the GWAS, PGS development, quantile regression, and non-equivalence tests.
(G and H) Only the non-exposed observations were used for the GWAS, PGS development, quantile regression, and non-equivalence tests.

point out that there are two causes, one of which, a deviation from unimodal trait distribution, we expect can be detected by inspection of the dataset. The other cause is gene-by-environment interactions, which our method can reveal by its characteristic pattern on plots of quantile-specific effect size versus quantile (Figure 4). We show that when an exposure or environment is suspected to play this role, then it is relatively straightforward to demonstrate that it gives rise to the predicted patterns by rerunning our analyses after stratifying by the putative exposure of interest. Figures 4 and 5 and the associated text explain how we envisage this working. Other work[24] further develops methods to explore and quantify the impact of $G \times E$ with context-specific PGS analyses, but it is much harder to confirm the role of the exposure or context if no interaction is suspected. One important clue to the identity of an exposure contributing a substantial $G \times E$ contribution to a trait that our analysis provides is that the smile characteristic of the $G \times E$ will cross the OLS at the point of stratification. For example, it could tell the investigator that what they are looking for should roughly divide the sample into two groups of individuals by a given ratio. The presence of multiple environmental effects poses a further level of difficulty, but we expect that modifications of our method may help in their detection.

Our approach has several limitations. The method does not work effectively when there are ties in the measurements or discrete outcomes. QR is not informative for discrete phenotype distributions with a small number of values because quantile-specific effect sizes will be zero for most quantiles. When adjustment covariates are projected out of a discrete phenotype, continuous residuals are formed, but if there is a small number of isolated modes, the quantile-specific effect sizes will just represent the spacing of the modes for the residual trait.

For example, in Figure S29, educational attainment (EA; years) is explored, but the quantile-specific effect sizes represent the proportions of study participants in common EA categories, such as secondary school or college degree completed, in a less usable format than a table of observed proportions in different EA categories. This approach is inappropriate for case-control data but should not have problems with formally discrete data such as systolic blood pressure, taking many integer values between 100 and 190 mmHg. Traits with multiple modes may be explored, but results should be interpreted with caution.

In summary, our method provides a computationally efficient approach for identifying trait-PGS pairs where the predictive value of the PGS varies substantially across the phenotypic range. Such non-uniform predictive values may be a consequence of unrecognized $G \times E$ interactions that can affect the predictive performance of PGSs across the population. Flagging traits that have substantially non-uniform predictability by their PGS should be part of any analysis plan using PGSs.

Data and code availability

Code for analyses and simulations is available on Zenodo (https://zenodo.org/records/14187837).

Acknowledgments

We thank the reviewers and editors of this article for their helpful comments and suggestions. We also thank the participants and everyone involved in curating and hosting the UK Biobank for making such a resource widely available.

Declaration of interests

The authors declare no competing interests.

Supplemental information

Supplemental information can be found online at https://doi.org/10.1016/j.ajhg.2025.04.013.

Received: November 14, 2023
Accepted: April 24, 2025
Published: June 5, 2025

References

1. Albiñana, C., Grove, J., McGrath, J.J., Agerbo, E., Wray, N.R., Bulik, C.M., Nordentoft, M., Hougaard, D.M., Werge, T., Børglum, A.D., et al. (2021). Leveraging both individual-level genetic data and GWAS summary statistics increases polygenic prediction. Am. J. Hum. Genet. *108*, 1001–1011.
2. Thompson, D.J., Wells, D., Selzam, S., Peneva, I., Moore, R., Sharp, K., Tarran, W.A., Beard, E.J., Riveros-Mckay, F., Giner-Delgado, C., et al. (2024). A systematic evaluation of the performance and properties of the UK Biobank Polygenic Risk Score (PRS) Release. PLoS One *19*, e0307270.
3. Kullo, I.J., Lewis, C.M., Inouye, M., Martin, A.R., Ripatti, S., and Chatterjee, N. (2022). Polygenic scores in biomedical research. Nat. Rev. Genet. *23*, 524–532.
4. Dudbridge, F. (2013). Power and predictive accuracy of polygenic risk scores. PLoS Genet. *9*, e1003348.
5. Khera, A.V., Chaffin, M., Aragam, K.G., Haas, M.E., Roselli, C., Choi, S.H., Natarajan, P., Lander, E.S., Lubitz, S.A., Ellinor, P.T., and Kathiresan, S. (2018). Genome-wide polygenic scores for common diseases identify individuals with risk equivalent to monogenic mutations. Nat. Genet. *50*, 1219–1224.
6. Collister, J.A., Liu, X., and Clifton, L. (2022). Calculating polygenic risk scores (PRS) in UK Biobank: a practical guide for epidemiologists. Front. Genet. *13*, 818574.
7. Hill, W.D., Hagenaars, S.P., Marioni, R.E., Harris, S.E., Liewald, D.C.M., Davies, G., Okbay, A., McIntosh, A.M., Gale, C.R., and Deary, I.J. (2016). Molecular genetic contributions to social deprivation and household income in UK Biobank. Curr. Biol. *26*, 3083–3089.
8. Patel, P.C., and Rietveld, C.A. (2019). Effect of genetic propensity for obesity on income and wealth through educational attainment. Obesity *27*, 1423–1427.
9. Okbay, A., Wu, Y., Wang, N., Jayashankar, H., Bennett, M., Nehzati, S.M., Sidorenko, J., Kweon, H., Goldman, G., Gjorgjieva, T., et al. (2022). Polygenic prediction of educational attainment within and between families from genome-wide association analyses in 3 million individuals. Nat. Genet. *54*, 437–449.
10. Rea-Sandin, G., Oro, V., Strouse, E., Clifford, S., Wilson, M. N., Shaw, D.S., and Lemery-Chalfant, K. (2021). Educational attainment polygenic score predicts inhibitory control and academic skills in early and middle childhood. Gene Brain Behav. *20*, e12762.
11. Ding, Y., Hou, K., Xu, Z., Pimplaskar, A., Petter, E., Boulier, K., Privé, F., Vilhjálmsson, B.J., Olde Loohuis, L.M., and Pasaniuc, B. (2023). Polygenic scoring accuracy varies across the genetic ancestry continuum. Nature *618*, 774–781.
12. Mostafavi, H., Harpak, A., Agarwal, I., Conley, D., Pritchard, J.K., and Przeworski, M. (2020). Variable prediction accuracy of polygenic scores within an ancestry group. Elife *9*, e48376.
13. Petter, E., Ding, Y., Hou, K., Bhattacharya, A., Gusev, A., Zaitlen, N., and Pasaniuc, B. (2023). Genotype error due to low-coverage sequencing induces uncertainty in polygenic scoring. Am. J. Hum. Genet. *110*, 1319–1329.
14. Marderstein, A.R., Kulm, S., Peng, C., Tamimi, R., Clark, A.G., and Elemento, O. (2021). A polygenic-score-based approach for identification of gene-drug interactions stratifying breast cancer risk. Am. J. Hum. Genet. *108*, 1752–1764.
15. Bycroft, C., Freeman, C., Petkova, D., Band, G., Elliott, L.T., Sharp, K., Motyer, A., Vukcevic, D., Delaneau, O., O'Connell, J., et al. (2018). The UK Biobank resource with deep phenotyping and genomic data. Nature *562*, 203–209.
16. Koenker, R., and Hallock, K.F. (2001). Quantile regression. J. Econ. Perspect. *15*, 143–156.
17. Viechtbauer, W. (2010). Conducting Meta-Analyses in R with the metafor Package. J. Stat. Softw. *36*, 1–48.
18. Abadi, A., Alyass, A., Robiou du Pont, S., Bolker, B., Singh, P., Mohan, V., Diaz, R., Engert, J.C., Yusuf, S., Gerstein, H.C., et al. (2017). Penetrance of polygenic obesity susceptibility loci across the body mass index distribution. Am. J. Hum. Genet. *101*, 925–938.

19. Lakens, D., Scheel, A.M., and Isager, P.M. (2018). Equivalence testing for psychological research: A tutorial. Advances in methods and practices in psychological science *1*, 259–269.
20. Meisner, A., Kundu, P., and Chatterjee, N. (2019). Case-only analysis of gene-environment interactions using polygenic risk scores. Am. J. Epidemiol. *188*, 2013–2020.
21. Sheppard, B., Rappoport, N., Loh, P.R., Sanders, S.J., Zaitlen, N., and Dahl, A. (2021). A model and test for coordinated polygenic epistasis in complex traits. Proc. Natl. Acad. Sci. USA *118*, e1922305118.
22. Borgen, N.T., Haupt, A., and Wiborg, Ø.N. (2021). A new framework for estimation of unconditional quantile treatment effects: The Residualized Quantile Regression (RQR) model. Preprint at SocArXiv. https://osf.io/preprints/socarxiv/42gcb_v1.
23. Pozarickij, A., Williams, C., Hysi, P.G., Guggenheim, J.A.; and UK Biobank Eye and Vision Consortium (2019). Quantile regression analysis reveals widespread evidence for gene-environment or gene-gene interactions in myopia development. Commun. Biol. *2*, 167.
24. Sadowski, M., Thompson, M., Mefford, J., Haldar, T., Oni-Orisan, A., Border, R., Pazokitoroudi, A., Cai, N., Ayroles, J.F., Sankararaman, S., et al. (2024). Characterizing the genetic architecture of drug response using gene-context interaction methods. Cell Genom. *4*, 100722.

The American Journal of Human Genetics, Volume *112*

Supplemental information

Beyond predictive R^2: Quantile regression and non-equivalence tests reveal complex relationships of traits and polygenic scores

Joel Mefford, Molly Smullen, Felix Zhang, Michal Sadowski, Richard Border, Andy Dahl, Jonathan Flint, and Noah Zaitlen

Supplement

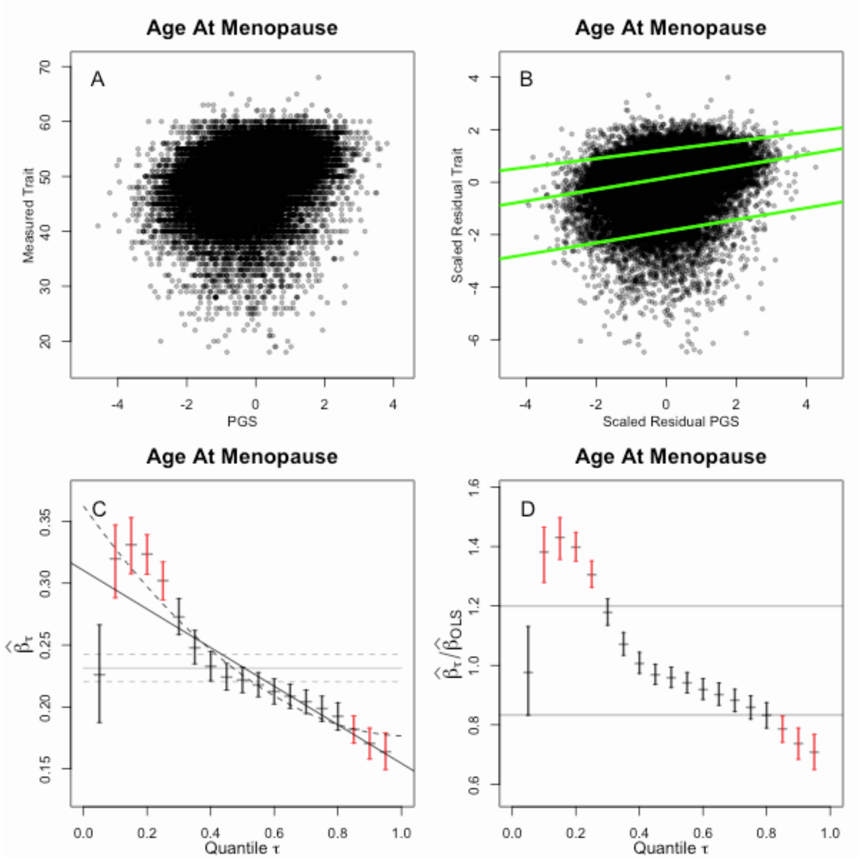

Figure S1. Age at menopause

Panels A and B show scatter plots of the trait (age at menopause) versus polygenic score for participants in the UK Biobank. In panel A and trait and PGS as reported in the data set are plotted. In panel B, residualized and scaled trait and PGS values are used, after projection of age, age^2, sex, age*sex, and ten genetic principal components and rescaling the residuals to mean zero variance one. The green lines show linear models fit to the data using QR at the 95th, 50th, and 5th quantiles of the residual trait distribution. QR is used to estimate quantile specific linear effect sizes $\widehat{\beta}_\tau$ at 19 quantiles τ of the residual trait distribution as in panel B. In panel C, $\widehat{\beta}_\tau$ is plotted against τ, with vertical bars representing 95% confidence intervals for $\widehat{\beta}_\tau$. The gray horizontal line with dashed lines above and below represent $\widehat{\beta}_{\mathrm{OLS}}$ and its 95% confidence interval for a linear model fit to residual data as in panel B. In panel D, $\widehat{\beta}_\tau/\widehat{\beta}_{\mathrm{OLS}}$ versus τ is plotted with vertical bars representing 95% confidence intervals for the ratio. Homogeneous effect sizes across the phenotypic distribution correspond to ratios $\widehat{\beta}_\tau/\widehat{\beta}_{\mathrm{OLS}}$ equal to one for each τ. The gray horizontal lines in panel D bound an equivalence band where the ratio is between $(1/\gamma, \gamma)$ for $\gamma = 1.2$. For ratios with the 95% confidence interval completely above or below the band, the vertical bars are colored red to indicate non-equivalence of the QR and OLS effect sizes and thus heterogeneity of $\widehat{\beta}_\tau$ and the linear predictive value of the PGS across the phenotypic range.

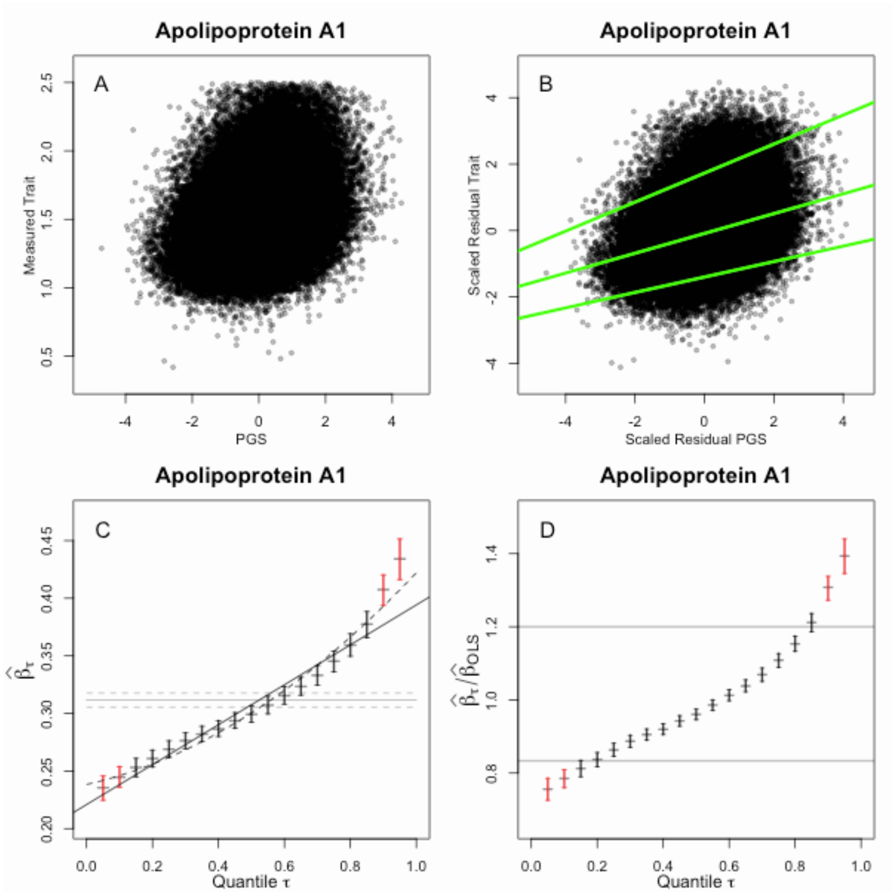

Figure S2. Apolipoprotein A1

Panels A and B show scatter plots of the trait (apolipoprotein A1) versus polygenic score for participants in the UK Biobank. In panel A and trait and PGS as reported in the data set are plotted. In panel B, residualized and scaled trait and PGS values are used, after projection of age, age^2, sex, age*sex, and ten genetic principal components and rescaling the residuals to mean zero variance one. The green lines show linear models fit to the data using QR at the 95th, 50th, and 5th quantiles of the residual trait distribution. QR is used to estimate quantile specific linear effect sizes $\widehat{\beta}_\tau$ at 19 quantiles τ of the residual trait distribution as in panel B. In panel C, $\widehat{\beta}_\tau$ is plotted against τ, with vertical bars representing 95% confidence intervals for $\widehat{\beta}_\tau$. The gray horizontal line with dashed lines above and below represent $\widehat{\beta}_{\mathrm{OLS}}$ and its 95% confidence interval for a linear model fit to residual data as in panel B. In panel D, $\widehat{\beta}_\tau/\widehat{\beta}_{\mathrm{OLS}}$ versus τ is plotted with vertical bars representing 95% confidence intervals for the ratio. Homogeneous effect sizes across the phenotypic distribution correspond to ratios $\widehat{\beta}_\tau/\widehat{\beta}_{\mathrm{OLS}}$ equal to one for each τ. The gray horizontal lines in panel D bound an equivalence band where the ratio is between $(1/\gamma, \gamma)$ for $\gamma = 1.2$. For ratios with the 95% confidence interval completely above or below the band, the vertical bars are colored red to indicate non-equivalence of the QR and OLS effect sizes and thus heterogeneity of $\widehat{\beta}_\tau$ and the linear predictive value of the PGS across the phenotypic range.

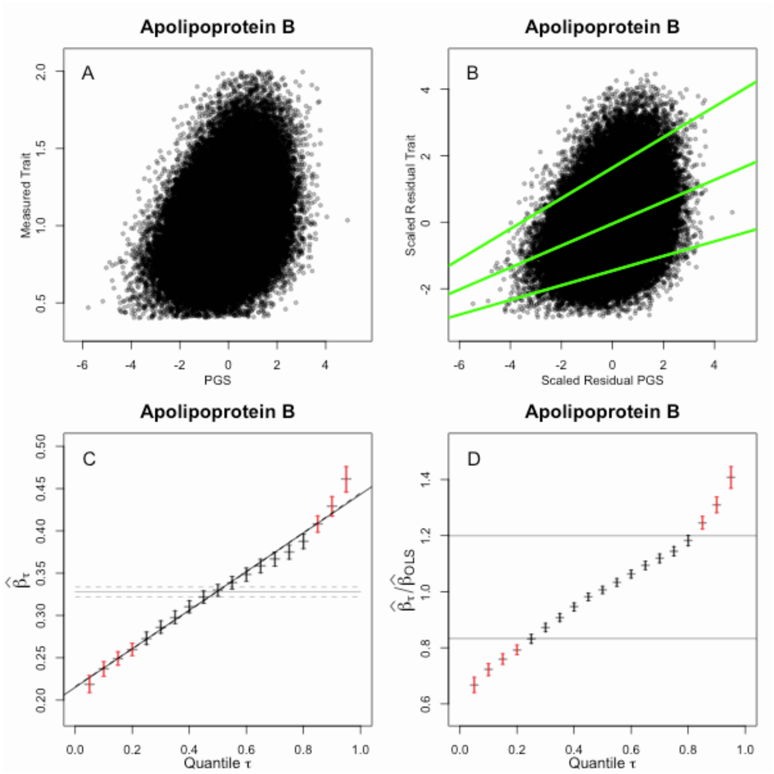

Figure S3. Apolipoprotein B

Panels A and B show scatter plots of the trait (apolipoprotein B) versus polygenic score for participants in the UK Biobank. In panel A and trait and PGS as reported in the data set are plotted. In panel B, residualized and scaled trait and PGS values are used, after projection of age, age^2, sex, age*sex, and ten genetic principal components and rescaling the residuals to mean zero variance one. The green lines show linear models fit to the data using QR at the 95th, 50th, and 5th quantiles of the residual trait distribution. QR is used to estimate quantile specific linear effect sizes $\widehat{\beta}_\tau$ at 19 quantiles τ of the residual trait distribution as in panel B. In panel C, $\widehat{\beta}_\tau$ is plotted against τ, with vertical bars representing 95% confidence intervals for $\widehat{\beta}_\tau$. The gray horizontal line with dashed lines above and below represent $\widehat{\beta}_{\mathrm{OLS}}$ and its 95% confidence interval for a linear model fit to residual data as in panel B. In panel D, $\widehat{\beta}_\tau/\widehat{\beta}_{\mathrm{OLS}}$ versus τ is plotted with vertical bars representing 95% confidence intervals for the ratio. Homogeneous effect sizes across the phenotypic distribution correspond to ratios $\widehat{\beta}_\tau/\widehat{\beta}_{\mathrm{OLS}}$ equal to one for each τ. The gray horizontal lines in panel D bound an equivalence band where the ratio is between $(1/\gamma, \gamma)$ for $\gamma = 1.2$. For ratios with the 95% confidence interval completely above or below the band, the vertical bars are colored red to indicate non-equivalence of the QR and OLS effect sizes and thus heterogeneity of $\widehat{\beta}_\tau$ and the linear predictive value of the PGS across the phenotypic range.

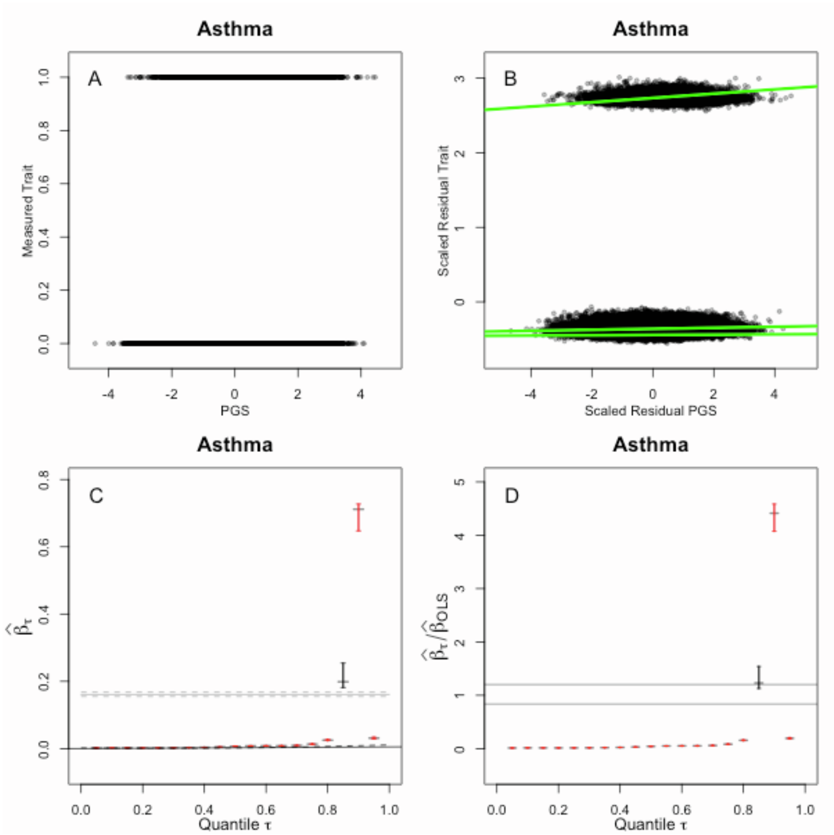

Figure S4. Asthma

Panels A and B show scatter plots of the trait (asthma) versus polygenic score for participants in the UK Biobank. In panel A and trait and PGS as reported in the data set are plotted. In panel B, residualized and scaled trait and PGS values are used, after projection of age, age^2, sex, age*sex, and ten genetic principal components and rescaling the residuals to mean zero variance one. The green lines show linear models fit to the data using QR at the 95th, 50th, and 5th quantiles of the residual trait distribution. QR is used to estimate quantile specific linear effect sizes $\widehat{\beta}_\tau$ at 19 quantiles τ of the residual trait distribution as in panel B. In panel C, $\widehat{\beta}_\tau$ is plotted against τ, with vertical bars representing 95% confidence intervals for $\widehat{\beta}_\tau$. The gray horizontal line with dashed lines above and below represent $\widehat{\beta}_{\mathrm{OLS}}$ and its 95% confidence interval for a linear model fit to residual data as in panel B. In panel D, $\widehat{\beta}_\tau/\widehat{\beta}_{\mathrm{OLS}}$ versus τ is plotted with vertical bars representing 95% confidence intervals for the ratio. Homogeneous effect sizes across the phenotypic distribution correspond to ratios $\widehat{\beta}_\tau/\widehat{\beta}_{\mathrm{OLS}}$ equal to one for each τ. The gray horizontal lines in panel D bound an equivalence band where the ratio is between $(1/\gamma, \gamma)$ for $\gamma = 1.2$. For ratios with the 95% confidence interval completely above or below the band, the vertical bars are colored red to indicate non-equivalence of the QR and OLS effect sizes and thus heterogeneity of $\widehat{\beta}_\tau$ and the linear predictive value of the PGS across the phenotypic range.

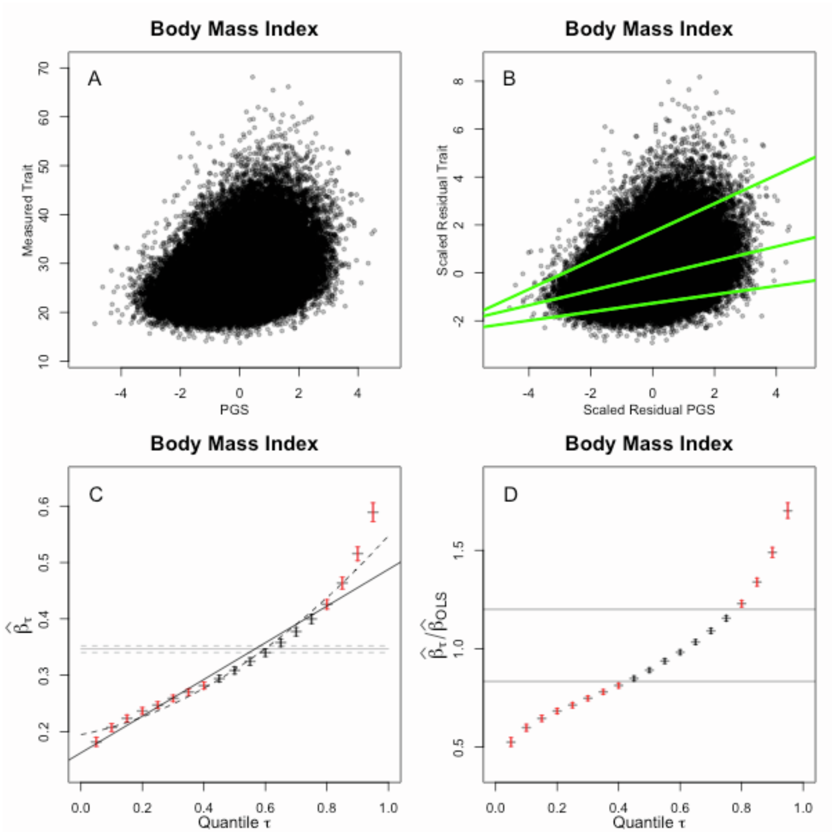

Figure S5. Body mass index

Panels A and B show scatter plots of the trait (body mass index; BMI) versus polygenic score for participants in the UK Biobank. In panel A and trait and PGS as reported in the data set are plotted. In panel B, residualized and scaled trait and PGS values are used, after projection of age, age^2, sex, age*sex, and ten genetic principal components and rescaling the residuals to mean zero variance one. The green lines show linear models fit to the data using QR at the 95th, 50th, and 5th quantiles of the residual trait distribution. QR is used to estimate quantile specific linear effect sizes $\widehat{\beta}_\tau$ at 19 quantiles τ of the residual trait distribution as in panel B. In panel C, $\widehat{\beta}_\tau$ is plotted against τ, with vertical bars representing 95% confidence intervals for $\widehat{\beta}_\tau$. The gray horizontal line with dashed lines above and below represent $\widehat{\beta}_{\mathrm{OLS}}$ and its 95% confidence interval for a linear model fit to residual data as in panel B. In panel D, $\widehat{\beta}_\tau/\widehat{\beta}_{\mathrm{OLS}}$ versus τ is plotted with vertical bars representing 95% confidence intervals for the ratio. Homogeneous effect sizes across the phenotypic distribution correspond to ratios $\widehat{\beta}_\tau/\widehat{\beta}_{\mathrm{OLS}}$ equal to one for each τ. The gray horizontal lines in panel D bound an equivalence band where the ratio is between $(1/\gamma, \gamma)$ for $\gamma = 1.2$. For ratios with the 95% confidence interval completely above or below the band, the vertical bars are colored red to indicate non-equivalence of the QR and OLS effect sizes and thus heterogeneity of $\widehat{\beta}_\tau$ and the linear predictive value of the PGS across the phenotypic range.

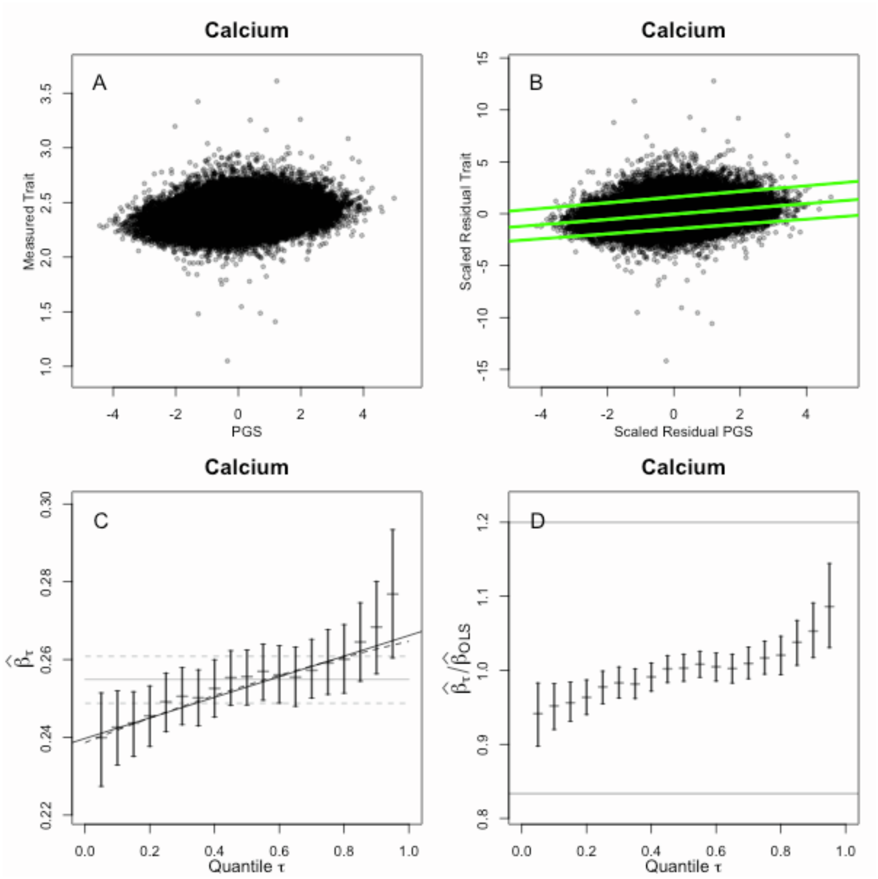

Figure S6. Calcium

Panels A and B show scatter plots of the trait (calcium) versus polygenic score for participants in the UK Biobank. In panel A and trait and PGS as reported in the data set are plotted. In panel B, residualized and scaled trait and PGS values are used, after projection of age, age^2, sex, age*sex, and ten genetic principal components and rescaling the residuals to mean zero variance one. The green lines show linear models fit to the data using QR at the 95th, 50th, and 5th quantiles of the residual trait distribution. QR is used to estimate quantile specific linear effect sizes $\widehat{\beta}_\tau$ at 19 quantiles τ of the residual trait distribution as in panel B. In panel C, $\widehat{\beta}_\tau$ is plotted against τ, with vertical bars representing 95% confidence intervals for $\widehat{\beta}_\tau$. The gray horizontal line with dashed lines above and below represent $\widehat{\beta}_{\mathrm{OLS}}$ and its 95% confidence interval for a linear model fit to residual data as in panel B. In panel D, $\widehat{\beta}_\tau/\widehat{\beta}_{\mathrm{OLS}}$ versus τ is plotted with vertical bars representing 95% confidence intervals for the ratio. Homogeneous effect sizes across the phenotypic distribution correspond to ratios $\widehat{\beta}_\tau/\widehat{\beta}_{\mathrm{OLS}}$ equal to one for each τ. The gray horizontal lines in panel D bound an equivalence band where the ratio is between $(1/\gamma, \gamma)$ for $\gamma = 1.2$. For ratios with the 95% confidence interval completely above or below the band, the vertical bars are colored red to indicate non-equivalence of the QR and OLS effect sizes and thus heterogeneity of $\widehat{\beta}_\tau$ and the linear predictive value of the PGS across the phenotypic range.

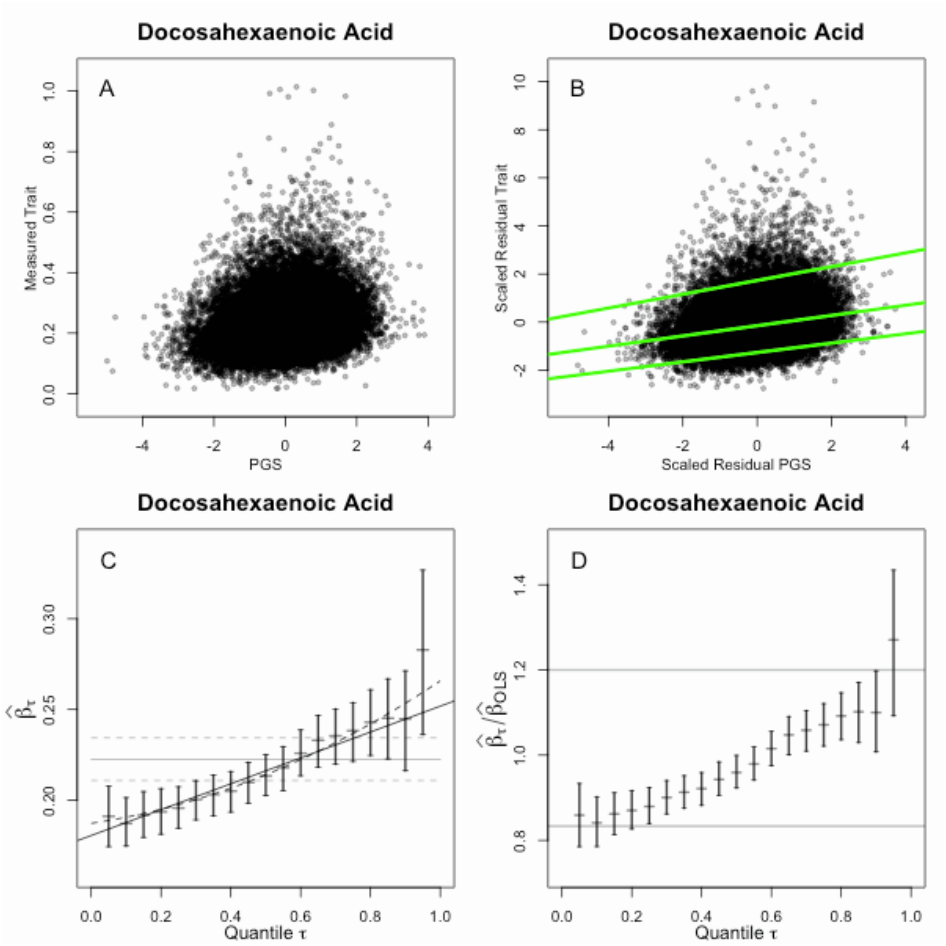

Figure S7. Docasahexaenoic Acid

Panels A and B show scatter plots of the trait (docasahexaenoic Acid) versus polygenic score for participants in the UK Biobank. In panel A and trait and PGS as reported in the data set are plotted. In panel B, residualized and scaled trait and PGS values are used, after projection of age, age^2, sex, age*sex, and ten genetic principal components and rescaling the residuals to mean zero variance one. The green lines show linear models fit to the data using QR at the 95th, 50th, and 5th quantiles of the residual trait distribution. QR is used to estimate quantile specific linear effect sizes $\widehat{\beta}_\tau$ at 19 quantiles τ of the residual trait distribution as in panel B. In panel C, $\widehat{\beta}_\tau$ is plotted against τ, with vertical bars representing 95% confidence intervals for $\widehat{\beta}_\tau$. The gray horizontal line with dashed lines above and below represent $\widehat{\beta}_{\mathrm{OLS}}$ and its 95% confidence interval for a linear model fit to residual data as in panel B. In panel D, $\widehat{\beta}_\tau/\widehat{\beta}_{\mathrm{OLS}}$ versus τ is plotted with vertical bars representing 95% confidence intervals for the ratio. Homogeneous effect sizes across the phenotypic distribution correspond to ratios $\widehat{\beta}_\tau/\widehat{\beta}_{\mathrm{OLS}}$ equal to one for each τ. The gray horizontal lines in panel D bound an equivalence band where the ratio is between $(1/\gamma, \gamma)$ for $\gamma = 1.2$. For ratios with the 95% confidence interval completely above or below the band, the vertical bars are colored red to indicate non-equivalence of the QR and OLS effect sizes and thus heterogeneity of $\widehat{\beta}_\tau$ and the linear predictive value of the PGS across the phenotypic range.

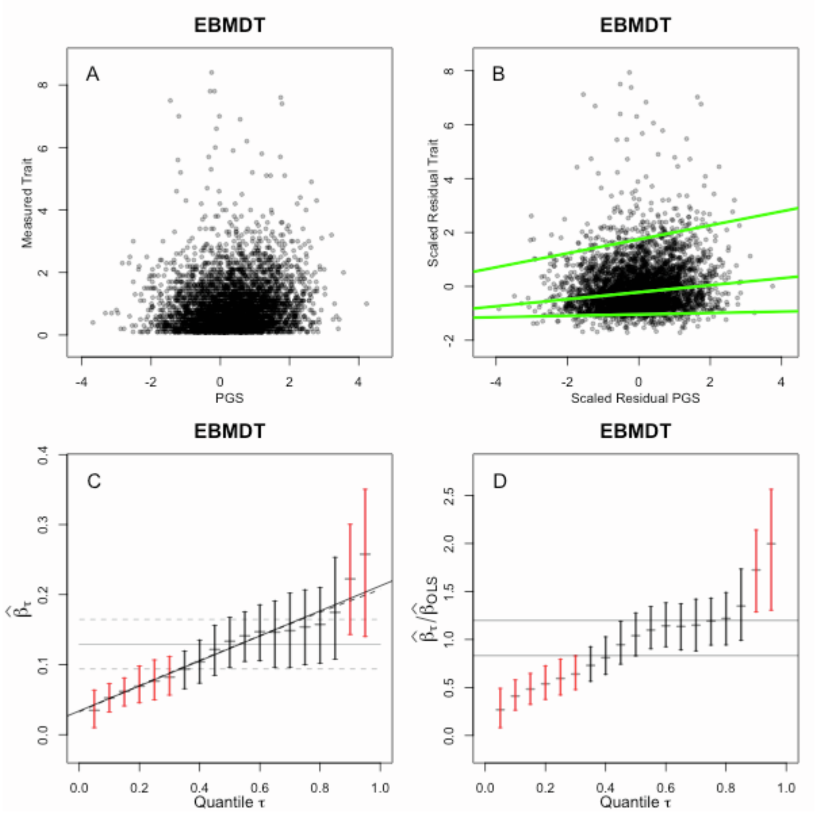

Figure S8. Estimated bone mineral density T score (EBMDT)

Panels A and B show scatter plots of the trait (estimated bone mineral density T score) versus polygenic score for participants in the UK Biobank. In panel A and trait and PGS as reported in the data set are plotted. In panel B, residualized and scaled trait and PGS values are used, after projection of age, age^2, sex, age*sex, and ten genetic principal components and rescaling the residuals to mean zero variance one. The green lines show linear models fit to the data using QR at the 95th, 50th, and 5th quantiles of the residual trait distribution. QR is used to estimate quantile specific linear effect sizes $\widehat{\beta}_\tau$ at 19 quantiles τ of the residual trait distribution as in panel B. In panel C, $\widehat{\beta}_\tau$ is plotted against τ, with vertical bars representing 95% confidence intervals for $\widehat{\beta}_\tau$. The gray horizontal line with dashed lines above and below represent $\widehat{\beta}_{\mathrm{OLS}}$ and its 95% confidence interval for a linear model fit to residual data as in panel B. In panel D, $\widehat{\beta}_\tau/\widehat{\beta}_{\mathrm{OLS}}$ versus τ is plotted with vertical bars representing 95% confidence intervals for the ratio. Homogeneous effect sizes across the phenotypic distribution correspond to ratios $\widehat{\beta}_\tau/\widehat{\beta}_{\mathrm{OLS}}$ equal to one for each τ. The gray horizontal lines in panel D bound an equivalence band where the ratio is between $(1/\gamma, \gamma)$ for $\gamma = 1.2$. For ratios with the 95% confidence interval completely above or below the band, the vertical bars are colored red to indicate non-equivalence of the QR and OLS effect sizes and thus heterogeneity of $\widehat{\beta}_\tau$ and the linear predictive value of the PGS across the phenotypic range.

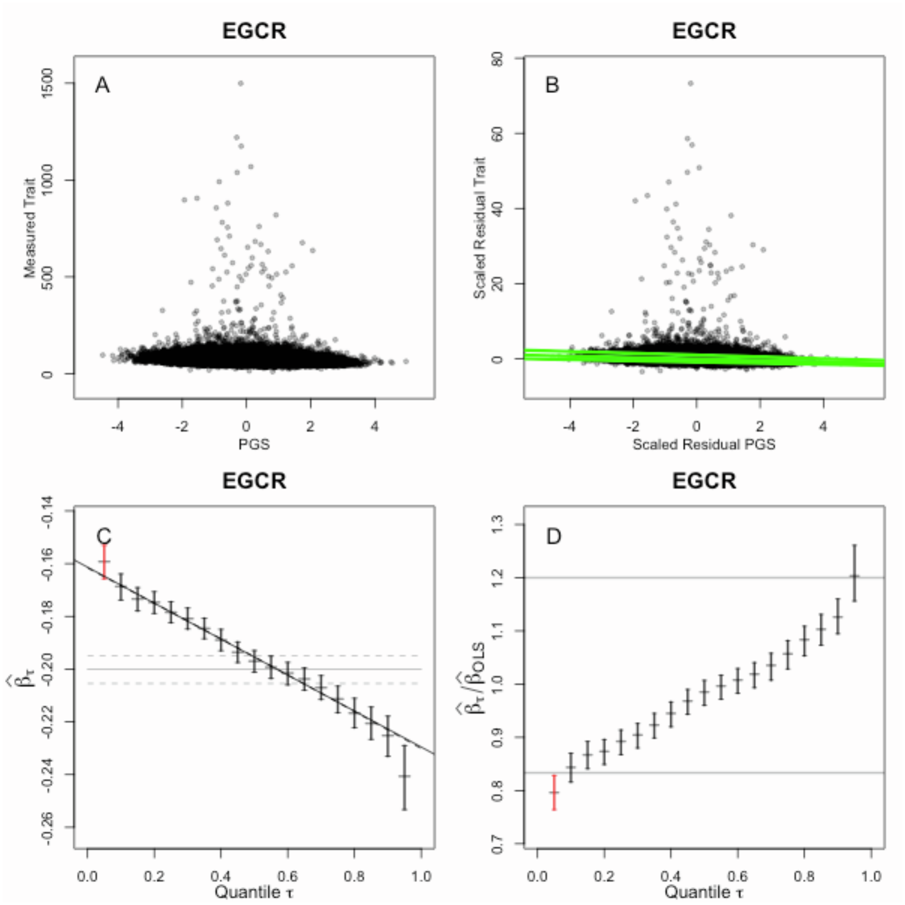

Figure S9. Estimated glomerular filtration rate, creatinin method, EGCR

Panels A and B show scatter plots of the trait (estimated glomerular filtration rate – creatinin method) versus polygenic score for participants in the UK Biobank. In panel A and trait and PGS as reported in the data set are plotted. In panel B, residualized and scaled trait and PGS values are used, after projection of age, age^2, sex, age*sex, and ten genetic principal components and rescaling the residuals to mean zero variance one. The green lines show linear models fit to the data using QR at the 95th, 50th, and 5th quantiles of the residual trait distribution. QR is used to estimate quantile specific linear effect sizes $\widehat{\beta}_\tau$ at 19 quantiles τ of the residual trait distribution as in panel B. In panel C, $\widehat{\beta}_\tau$ is plotted against τ, with vertical bars representing 95% confidence intervals for $\widehat{\beta}_\tau$. The gray horizontal line with dashed lines above and below represent $\widehat{\beta}_{\mathrm{OLS}}$ and its 95% confidence interval for a linear model fit to residual data as in panel B. In panel D, $\widehat{\beta}_\tau/\widehat{\beta}_{\mathrm{OLS}}$ versus τ is plotted with vertical bars representing 95% confidence intervals for the ratio. Homogeneous effect sizes across the phenotypic distribution correspond to ratios $\widehat{\beta}_\tau/\widehat{\beta}_{\mathrm{OLS}}$ equal to one for each τ. The gray horizontal lines in panel D bound an equivalence band where the ratio is between $(1/\gamma, \gamma)$ for $\gamma = 1.2$. For ratios with the 95% confidence interval completely above or below the band, the vertical bars are colored red to indicate non-equivalence of the QR and OLS effect sizes and thus heterogeneity of $\widehat{\beta}_\tau$ and the linear predictive value of the PGS across the phenotypic range.

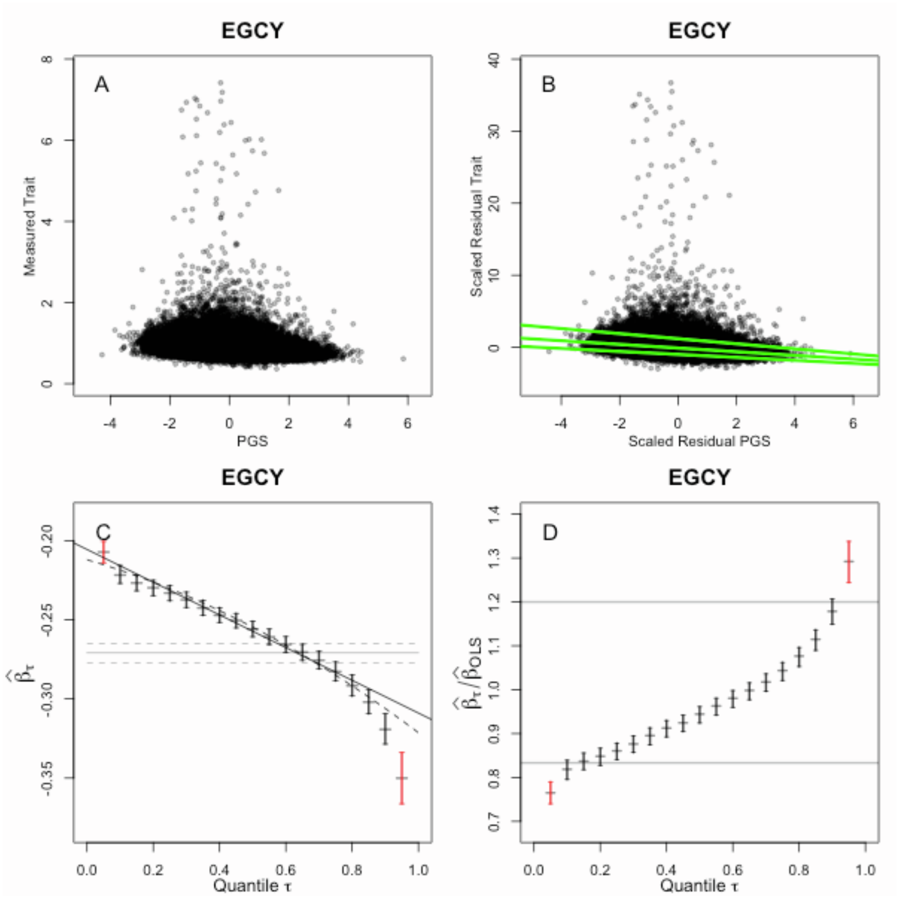

Figure S10. Estimated glomerular filtration rate cystatin method, EGCY

Panels A and B show scatter plots of the trait (estimated glomerular filtration rate – cystatin method) versus polygenic score for participants in the UK Biobank. In panel A and trait and PGS as reported in the data set are plotted. In panel B, residualized and scaled trait and PGS values are used, after projection of age, age^2, sex, age*sex, and ten genetic principal components and rescaling the residuals to mean zero variance one. The green lines show linear models fit to the data using QR at the 95th, 50th, and 5th quantiles of the residual trait distribution. QR is used to estimate quantile specific linear effect sizes $\widehat{\beta}_\tau$ at 19 quantiles τ of the residual trait distribution as in panel B. In panel C, $\widehat{\beta}_\tau$ is plotted against τ, with vertical bars representing 95% confidence intervals for $\widehat{\beta}_\tau$. The gray horizontal line with dashed lines above and below represent $\widehat{\beta}_{\mathrm{OLS}}$ and its 95% confidence interval for a linear model fit to residual data as in panel B. In panel D, $\widehat{\beta}_\tau/\widehat{\beta}_{\mathrm{OLS}}$ versus τ is plotted with vertical bars representing 95% confidence intervals for the ratio. Homogeneous effect sizes across the phenotypic distribution correspond to ratios $\widehat{\beta}_\tau/\widehat{\beta}_{\mathrm{OLS}}$ equal to one for each τ. The gray horizontal lines in panel D bound an equivalence band where the ratio is between $(1/\gamma, \gamma)$ for $\gamma = 1.2$. For ratios with the 95% confidence interval completely above or below the band, the vertical bars are colored red to indicate non-equivalence of the QR and OLS effect sizes and thus heterogeneity of $\widehat{\beta}_\tau$ and the linear predictive value of the PGS across the phenotypic range.

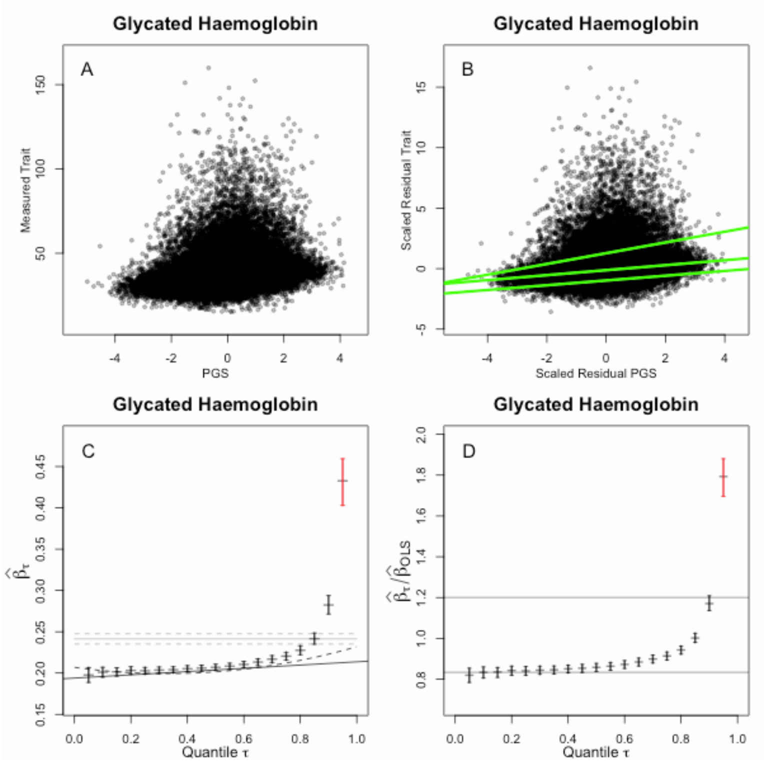

Figure S11. Glycated haemoglobin, HbA1c

Panels A and B show scatter plots of the trait (glycated haemoglobin, HbA1c) versus polygenic score for participants in the UK Biobank. In panel A and trait and PGS as reported in the data set are plotted. In panel B, residualized and scaled trait and PGS values are used, after projection of age, age^2, sex, age*sex, and ten genetic principal components and rescaling the residuals to mean zero variance one. The green lines show linear models fit to the data using QR at the 95th, 50th, and 5th quantiles of the residual trait distribution. QR is used to estimate quantile specific linear effect sizes $\widehat{\beta}_\tau$ at 19 quantiles τ of the residual trait distribution as in panel B. In panel C, $\widehat{\beta}_\tau$ is plotted against τ, with vertical bars representing 95% confidence intervals for $\widehat{\beta}_\tau$. The gray horizontal line with dashed lines above and below represent $\widehat{\beta}_{\mathrm{OLS}}$ and its 95% confidence interval for a linear model fit to residual data as in panel B. In panel D, $\widehat{\beta}_\tau/\widehat{\beta}_{\mathrm{OLS}}$ versus τ is plotted with vertical bars representing 95% confidence intervals for the ratio. Homogeneous effect sizes across the phenotypic distribution correspond to ratios $\widehat{\beta}_\tau/\widehat{\beta}_{\mathrm{OLS}}$ equal to one for each τ. The gray horizontal lines in panel D bound an equivalence band where the ratio is between $(1/\gamma, \gamma)$ for $\gamma = 1.2$. For ratios with the 95% confidence interval completely above or below the band, the vertical bars are colored red to indicate non-equivalence of the QR and OLS effect sizes and thus heterogeneity of $\widehat{\beta}_\tau$ and the linear predictive value of the PGS across the phenotypic range.

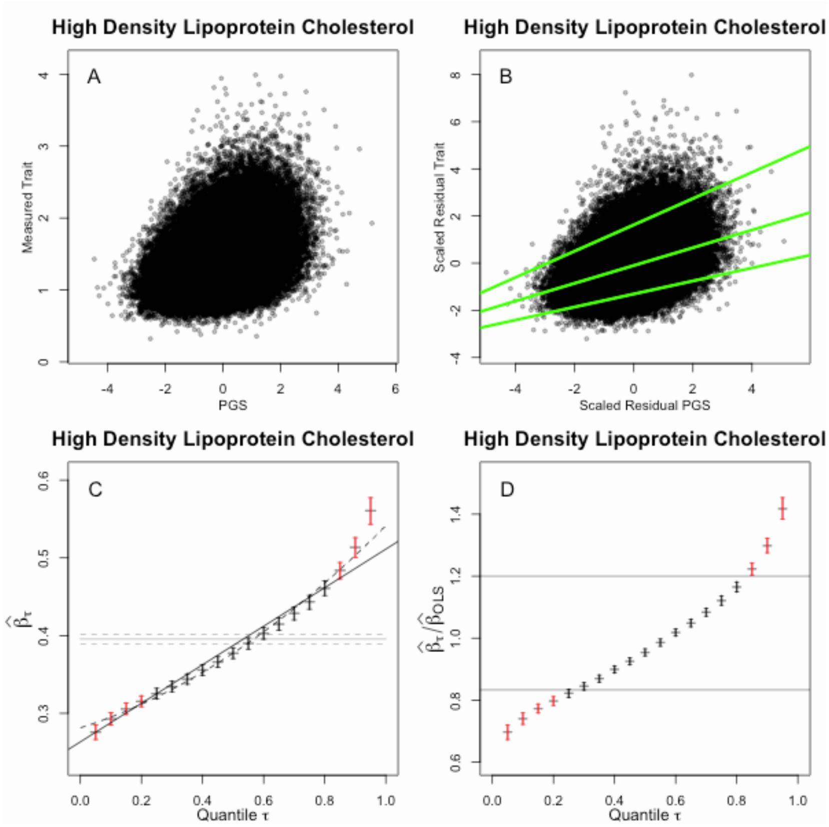

Figure S12. High density lipoprotein cholesterol, HDLc

Panels A and B show scatter plots of the trait (high density lipoprotein cholesterol, HDLc) versus polygenic score for participants in the UK Biobank. In panel A and trait and PGS as reported in the data set are plotted. In panel B, residualized and scaled trait and PGS values are used, after projection of age, age^2, sex, age*sex, and ten genetic principal components and rescaling the residuals to mean zero variance one. The green lines show linear models fit to the data using QR at the 95th, 50th, and 5th quantiles of the residual trait distribution. QR is used to estimate quantile specific linear effect sizes $\widehat{\beta}_\tau$ at 19 quantiles τ of the residual trait distribution as in panel B. In panel C, $\widehat{\beta}_\tau$ is plotted against τ, with vertical bars representing 95% confidence intervals for $\widehat{\beta}_\tau$. The gray horizontal line with dashed lines above and below represent $\widehat{\beta}_{\mathrm{OLS}}$ and its 95% confidence interval for a linear model fit to residual data as in panel B. In panel D, $\widehat{\beta}_\tau/\widehat{\beta}_{\mathrm{OLS}}$ versus τ is plotted with vertical bars representing 95% confidence intervals for the ratio. Homogeneous effect sizes across the phenotypic distribution correspond to ratios $\widehat{\beta}_\tau/\widehat{\beta}_{\mathrm{OLS}}$ equal to one for each τ. The gray horizontal lines in panel D bound an equivalence band where the ratio is between $(1/\gamma, \gamma)$ for $\gamma = 1.2$. For ratios with the 95% confidence interval completely above or below the band, the vertical bars are colored red to indicate non-equivalence of the QR and OLS effect sizes and thus heterogeneity of $\widehat{\beta}_\tau$ and the linear predictive value of the PGS across the phenotypic range.

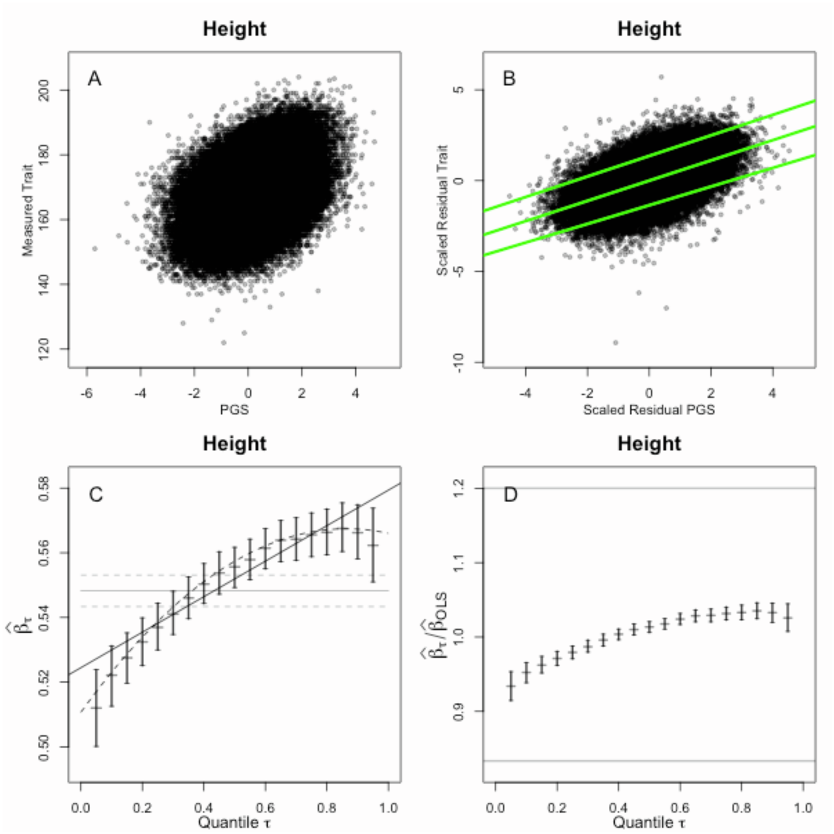

Figure S13. Height

Panels A and B show scatter plots of the trait (height) versus polygenic score for participants in the UK Biobank. In panel A and trait and PGS as reported in the data set are plotted. In panel B, residualized and scaled trait and PGS values are used, after projection of age, age^2, sex, age*sex, and ten genetic principal components and rescaling the residuals to mean zero variance one. The green lines show linear models fit to the data using QR at the 95th, 50th, and 5th quantiles of the residual trait distribution. QR is used to estimate quantile specific linear effect sizes $\widehat{\beta}_\tau$ at 19 quantiles τ of the residual trait distribution as in panel B. In panel C, $\widehat{\beta}_\tau$ is plotted against τ, with vertical bars representing 95% confidence intervals for $\widehat{\beta}_\tau$. The gray horizontal line with dashed lines above and below represent $\widehat{\beta}_{\mathrm{OLS}}$ and its 95% confidence interval for a linear model fit to residual data as in panel B. In panel D, $\widehat{\beta}_\tau/\widehat{\beta}_{\mathrm{OLS}}$ versus τ is plotted with vertical bars representing 95% confidence intervals for the ratio. Homogeneous effect sizes across the phenotypic distribution correspond to ratios $\widehat{\beta}_\tau/\widehat{\beta}_{\mathrm{OLS}}$ equal to one for each τ. The gray horizontal lines in panel D bound an equivalence band where the ratio is between $(1/\gamma, \gamma)$ for $\gamma = 1.2$. For ratios with the 95% confidence interval completely above or below the band, the vertical bars are colored red to indicate non-equivalence of the QR and OLS effect sizes and thus heterogeneity of $\widehat{\beta}_\tau$ and the linear predictive value of the PGS across the phenotypic range.

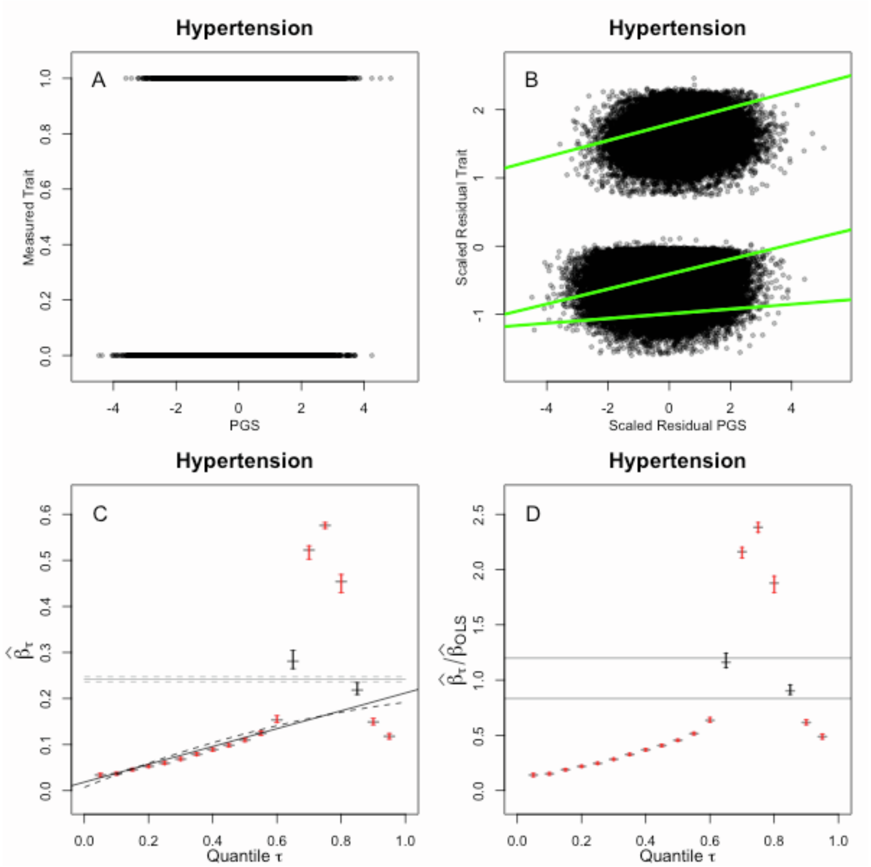

Figure S14. Hypertension

Panels A and B show scatter plots of the trait (hypertension) versus polygenic score for participants in the UK Biobank. In panel A and trait and PGS as reported in the data set are plotted. In panel B, residualized and scaled trait and PGS values are used, after projection of age, age^2, sex, age*sex, and ten genetic principal components and rescaling the residuals to mean zero variance one. The green lines show linear models fit to the data using QR at the 95th, 50th, and 5th quantiles of the residual trait distribution. QR is used to estimate quantile specific linear effect sizes $\widehat{\beta}_\tau$ at 19 quantiles τ of the residual trait distribution as in panel B. In panel C, $\widehat{\beta}_\tau$ is plotted against τ, with vertical bars representing 95% confidence intervals for $\widehat{\beta}_\tau$. The gray horizontal line with dashed lines above and below represent $\widehat{\beta}_{\mathrm{OLS}}$ and its 95% confidence interval for a linear model fit to residual data as in panel B. In panel D, $\widehat{\beta}_\tau/\widehat{\beta}_{\mathrm{OLS}}$ versus τ is plotted with vertical bars representing 95% confidence intervals for the ratio. Homogeneous effect sizes across the phenotypic distribution correspond to ratios $\widehat{\beta}_\tau/\widehat{\beta}_{\mathrm{OLS}}$ equal to one for each τ. The gray horizontal lines in panel D bound an equivalence band where the ratio is between $(1/\gamma, \gamma)$ for $\gamma = 1.2$. For ratios with the 95% confidence interval completely above or below the band, the vertical bars are colored red to indicate non-equivalence of the QR and OLS effect sizes and thus heterogeneity of $\widehat{\beta}_\tau$ and the linear predictive value of the PGS across the phenotypic range.

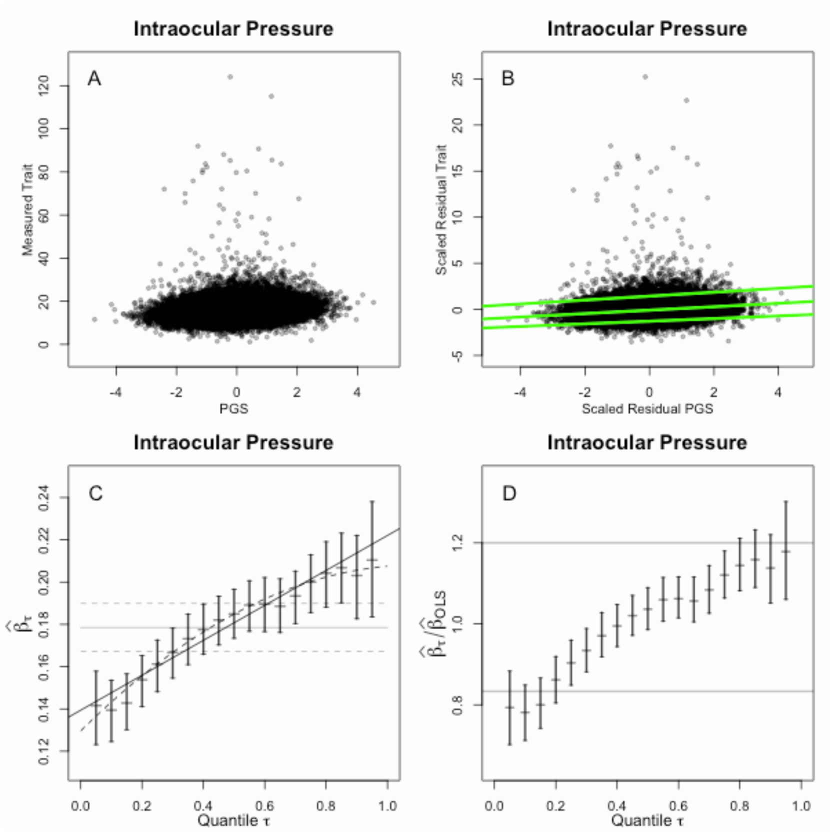

Figure S15. Intraocular Pressure

Panels A and B show scatter plots of the trait (intraocular pressure) versus polygenic score for participants in the UK Biobank. In panel A and trait and PGS as reported in the data set are plotted. In panel B, residualized and scaled trait and PGS values are used, after projection of age, age^2, sex, age*sex, and ten genetic principal components and rescaling the residuals to mean zero variance one. The green lines show linear models fit to the data using QR at the 95th, 50th, and 5th quantiles of the residual trait distribution. QR is used to estimate quantile specific linear effect sizes $\widehat{\beta}_\tau$ at 19 quantiles τ of the residual trait distribution as in panel B. In panel C, $\widehat{\beta}_\tau$ is plotted against τ, with vertical bars representing 95% confidence intervals for $\widehat{\beta}_\tau$. The gray horizontal line with dashed lines above and below represent $\widehat{\beta}_{\mathrm{OLS}}$ and its 95% confidence interval for a linear model fit to residual data as in panel B. In panel D, $\widehat{\beta}_\tau/\widehat{\beta}_{\mathrm{OLS}}$ versus τ is plotted with vertical bars representing 95% confidence intervals for the ratio. Homogeneous effect sizes across the phenotypic distribution correspond to ratios $\widehat{\beta}_\tau/\widehat{\beta}_{\mathrm{OLS}}$ equal to one for each τ. The gray horizontal lines in panel D bound an equivalence band where the ratio is between $(1/\gamma, \gamma)$ for $\gamma = 1.2$. For ratios with the 95% confidence interval completely above or below the band, the vertical bars are colored red to indicate non-equivalence of the QR and OLS effect sizes and thus heterogeneity of $\widehat{\beta}_\tau$ and the linear predictive value of the PGS across the phenotypic range.

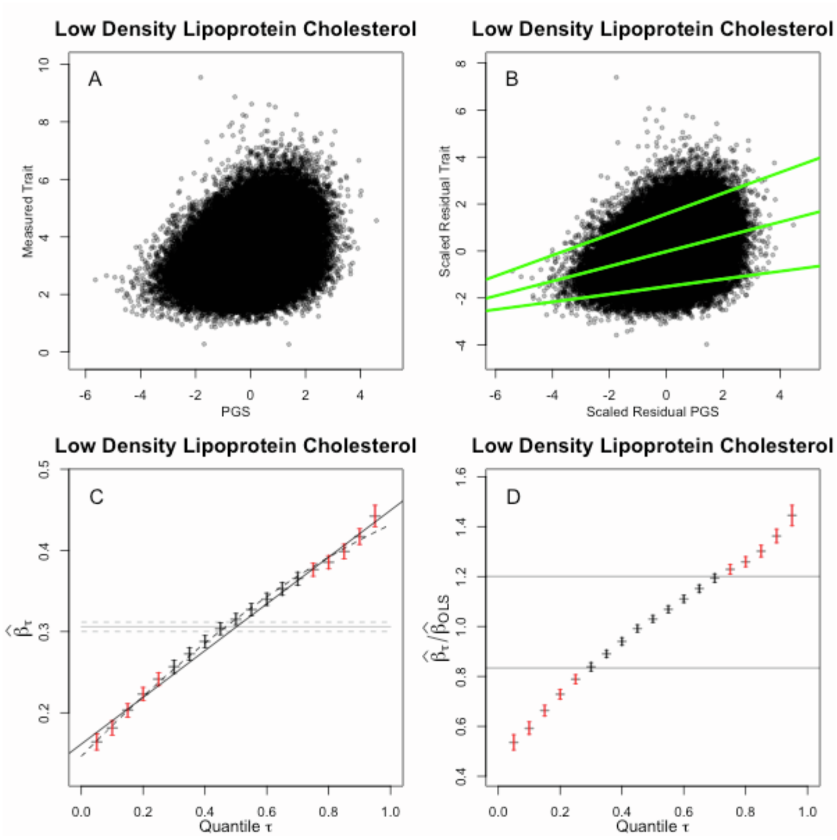

Figure S16. Low density lipoprotein cholesterol, LDLc

Panels A and B show scatter plots of the trait (low density lipoprotein cholesterol, LDLc) versus polygenic score for participants in the UK Biobank. In panel A and trait and PGS as reported in the data set are plotted. In panel B, residualized and scaled trait and PGS values are used, after projection of age, age^2, sex, age*sex, and ten genetic principal components and rescaling the residuals to mean zero variance one. The green lines show linear models fit to the data using QR at the 95th, 50th, and 5th quantiles of the residual trait distribution. QR is used to estimate quantile specific linear effect sizes $\widehat{\beta}_\tau$ at 19 quantiles τ of the residual trait distribution as in panel B. In panel C, $\widehat{\beta}_\tau$ is plotted against τ, with vertical bars representing 95% confidence intervals for $\widehat{\beta}_\tau$. The gray horizontal line with dashed lines above and below represent $\widehat{\beta}_{\mathrm{OLS}}$ and its 95% confidence interval for a linear model fit to residual data as in panel B. In panel D, $\widehat{\beta}_\tau/\widehat{\beta}_{\mathrm{OLS}}$ versus τ is plotted with vertical bars representing 95% confidence intervals for the ratio. Homogeneous effect sizes across the phenotypic distribution correspond to ratios $\widehat{\beta}_\tau/\widehat{\beta}_{\mathrm{OLS}}$ equal to one for each τ. The gray horizontal lines in panel D bound an equivalence band where the ratio is between $(1/\gamma, \gamma)$ for $\gamma = 1.2$. For ratios with the 95% confidence interval completely above or below the band, the vertical bars are colored red to indicate non-equivalence of the QR and OLS effect sizes and thus heterogeneity of $\widehat{\beta}_\tau$ and the linear predictive value of the PGS across the phenotypic range.

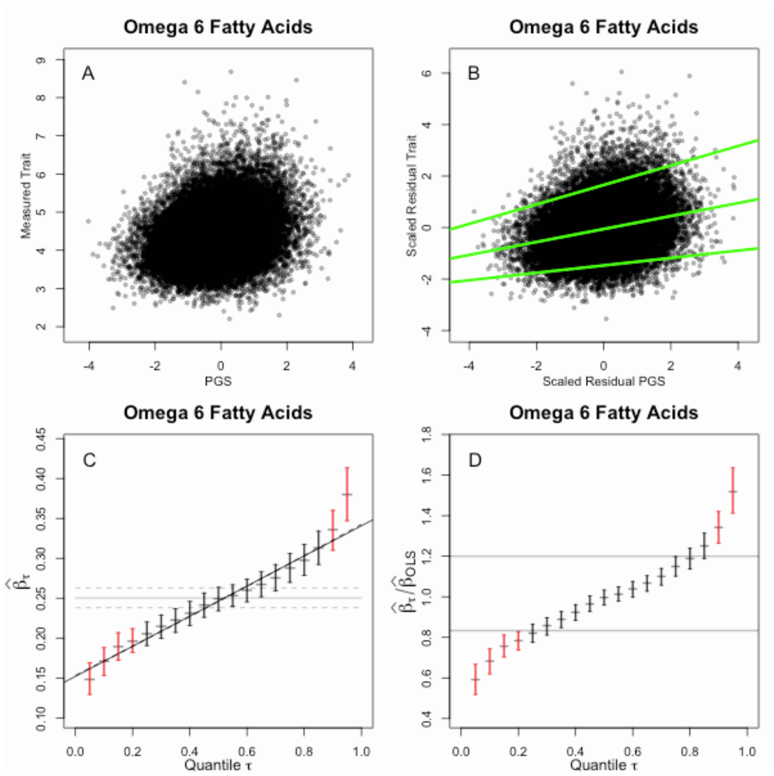

Figure S17. Omega 6 fatty acids

Panels A and B show scatter plots of the trait (omega 6 fatty acids) versus polygenic score for participants in the UK Biobank. In panel A and trait and PGS as reported in the data set are plotted. In panel B, residualized and scaled trait and PGS values are used, after projection of age, age^2, sex, age*sex, and ten genetic principal components and rescaling the residuals to mean zero variance one. The green lines show linear models fit to the data using QR at the 95th, 50th, and 5th quantiles of the residual trait distribution. QR is used to estimate quantile specific linear effect sizes $\widehat{\beta}_\tau$ at 19 quantiles τ of the residual trait distribution as in panel B. In panel C, $\widehat{\beta}_\tau$ is plotted against τ, with vertical bars representing 95% confidence intervals for $\widehat{\beta}_\tau$. The gray horizontal line with dashed lines above and below represent $\widehat{\beta}_{\mathrm{OLS}}$ and its 95% confidence interval for a linear model fit to residual data as in panel B. In panel D, $\widehat{\beta}_\tau/\widehat{\beta}_{\mathrm{OLS}}$ versus τ is plotted with vertical bars representing 95% confidence intervals for the ratio. Homogeneous effect sizes across the phenotypic distribution correspond to ratios $\widehat{\beta}_\tau/\widehat{\beta}_{\mathrm{OLS}}$ equal to one for each τ. The gray horizontal lines in panel D bound an equivalence band where the ratio is between $(1/\gamma, \gamma)$ for $\gamma = 1.2$. For ratios with the 95% confidence interval completely above or below the band, the vertical bars are colored red to indicate non-equivalence of the QR and OLS effect sizes and thus heterogeneity of $\widehat{\beta}_\tau$ and the linear predictive value of the PGS across the phenotypic range.

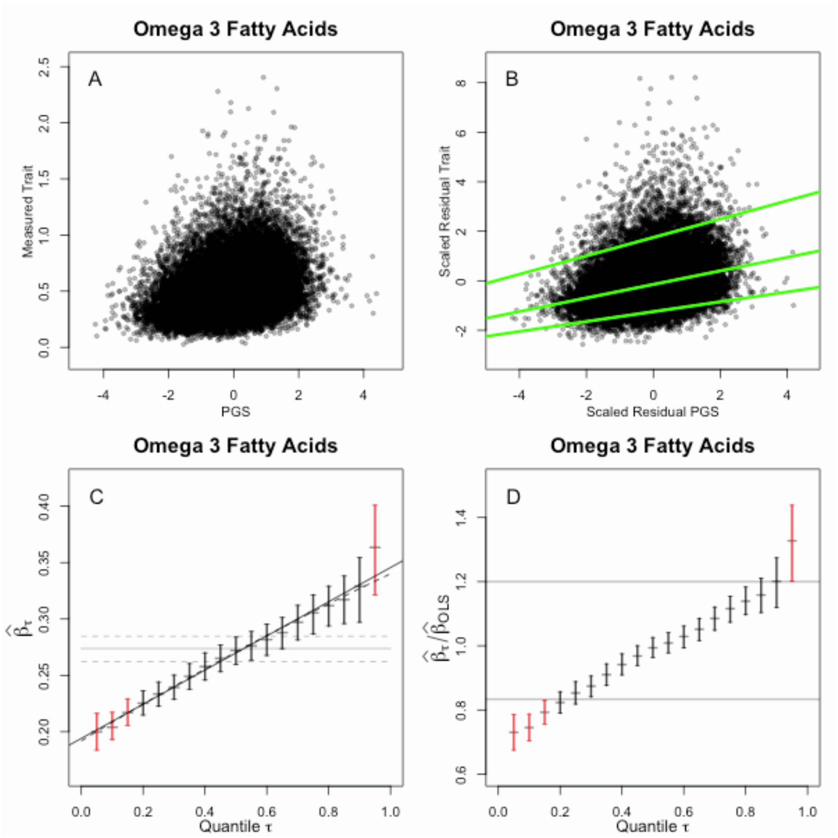

Figure S18. Omega 3 fatty acids

Panels A and B show scatter plots of the trait (omega 3 fatty acids) versus polygenic score for participants in the UK Biobank. In panel A and trait and PGS as reported in the data set are plotted. In panel B, residualized and scaled trait and PGS values are used, after projection of age, age^2, sex, age*sex, and ten genetic principal components and rescaling the residuals to mean zero variance one. The green lines show linear models fit to the data using QR at the 95th, 50th, and 5th quantiles of the residual trait distribution. QR is used to estimate quantile specific linear effect sizes $\widehat{\beta}_\tau$ at 19 quantiles τ of the residual trait distribution as in panel B. In panel C, $\widehat{\beta}_\tau$ is plotted against τ, with vertical bars representing 95% confidence intervals for $\widehat{\beta}_\tau$. The gray horizontal line with dashed lines above and below represent $\widehat{\beta}_{\mathrm{OLS}}$ and its 95% confidence interval for a linear model fit to residual data as in panel B. In panel D, $\widehat{\beta}_\tau/\widehat{\beta}_{\mathrm{OLS}}$ versus τ is plotted with vertical bars representing 95% confidence intervals for the ratio. Homogeneous effect sizes across the phenotypic distribution correspond to ratios $\widehat{\beta}_\tau/\widehat{\beta}_{\mathrm{OLS}}$ equal to one for each τ. The gray horizontal lines in panel D bound an equivalence band where the ratio is between $(1/\gamma, \gamma)$ for $\gamma = 1.2$. For ratios with the 95% confidence interval completely above or below the band, the vertical bars are colored red to indicate non-equivalence of the QR and OLS effect sizes and thus heterogeneity of $\widehat{\beta}_\tau$ and the linear predictive value of the PGS across the phenotypic range.

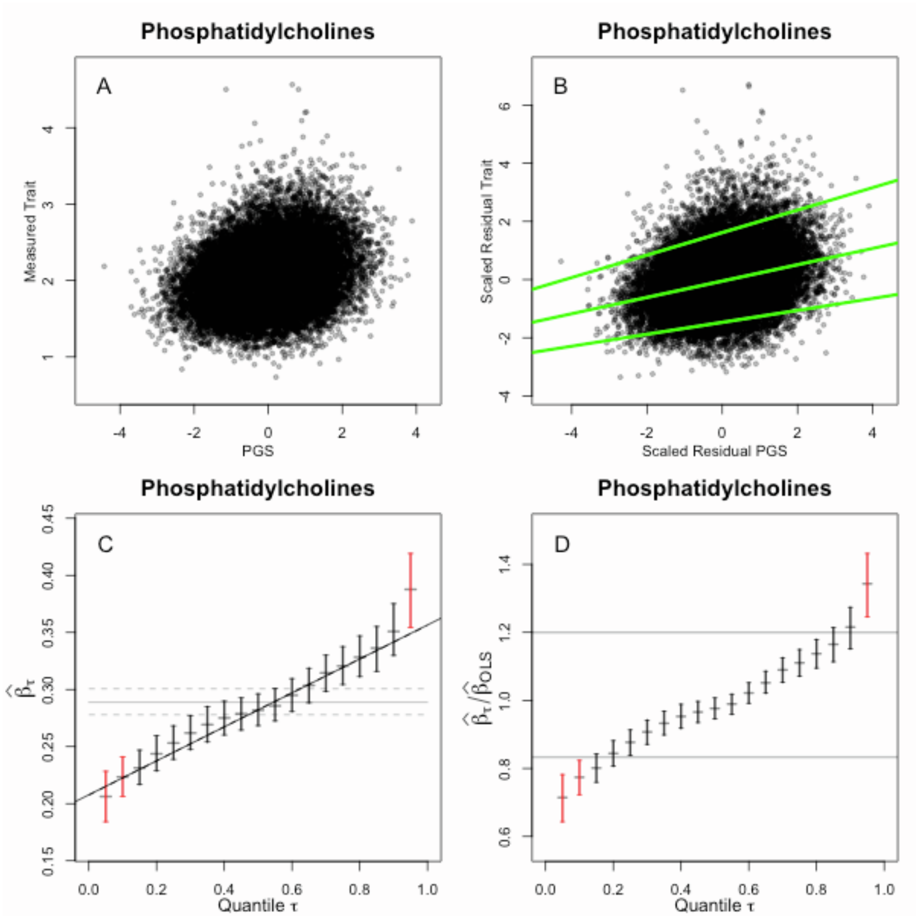

Figure S19. Phosphatidylcholines

Panels A and B show scatter plots of the trait (phosphatidylcholines) versus polygenic score for participants in the UK Biobank. In panel A and trait and PGS as reported in the data set are plotted. In panel B, residualized and scaled trait and PGS values are used, after projection of age, age^2, sex, age*sex, and ten genetic principal components and rescaling the residuals to mean zero variance one. The green lines show linear models fit to the data using QR at the 95th, 50th, and 5th quantiles of the residual trait distribution. QR is used to estimate quantile specific linear effect sizes $\widehat{\beta}_\tau$ at 19 quantiles τ of the residual trait distribution as in panel B. In panel C, $\widehat{\beta}_\tau$ is plotted against τ, with vertical bars representing 95% confidence intervals for $\widehat{\beta}_\tau$. The gray horizontal line with dashed lines above and below represent $\widehat{\beta}_{\text{OLS}}$ and its 95% confidence interval for a linear model fit to residual data as in panel B. In panel D, $\widehat{\beta}_\tau/\widehat{\beta}_{\text{OLS}}$ versus τ is plotted with vertical bars representing 95% confidence intervals for the ratio. Homogeneous effect sizes across the phenotypic distribution correspond to ratios $\widehat{\beta}_\tau/\widehat{\beta}_{\text{OLS}}$ equal to one for each τ. The gray horizontal lines in panel D bound an equivalence band where the ratio is between $(1/\gamma, \gamma)$ for $\gamma = 1.2$. For ratios with the 95% confidence interval completely above or below the band, the vertical bars are colored red to indicate non-equivalence of the QR and OLS effect sizes and thus heterogeneity of $\widehat{\beta}_\tau$ and the linear predictive value of the PGS across the phenotypic range.

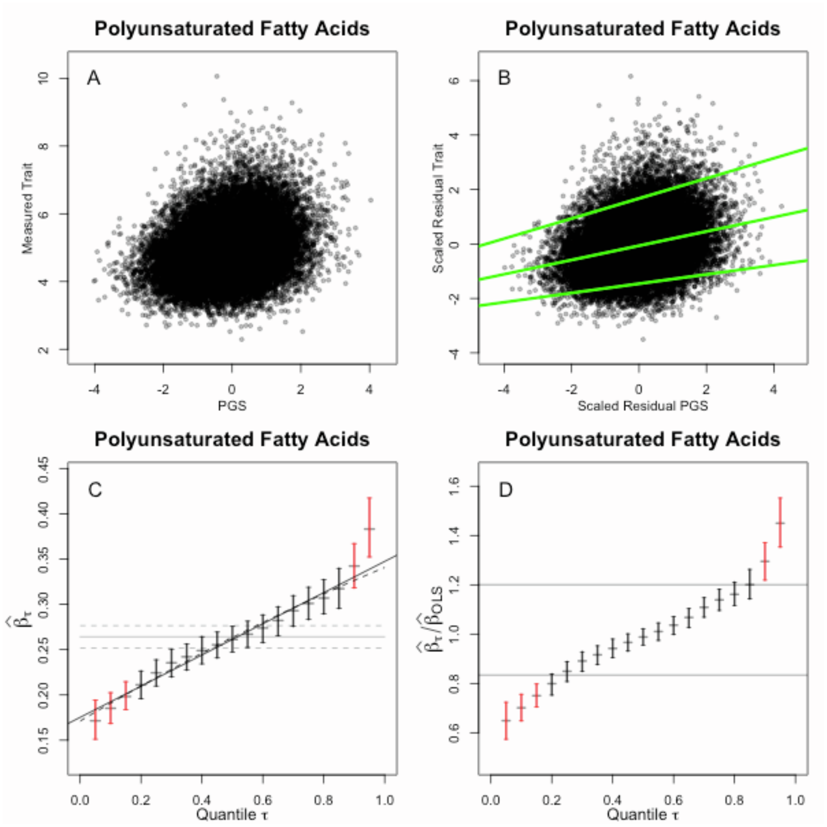

Figure S20. Polyunsaturated fatty acids

Panels A and B show scatter plots of the trait (polyunsaturated fatty acids) versus polygenic score for participants in the UK Biobank. In panel A and trait and PGS as reported in the data set are plotted. In panel B, residualized and scaled trait and PGS values are used, after projection of age, age^2, sex, age*sex, and ten genetic principal components and rescaling the residuals to mean zero variance one. The green lines show linear models fit to the data using QR at the 95th, 50th, and 5th quantiles of the residual trait distribution. QR is used to estimate quantile specific linear effect sizes $\widehat{\beta}_\tau$ at 19 quantiles τ of the residual trait distribution as in panel B. In panel C, $\widehat{\beta}_\tau$ is plotted against τ, with vertical bars representing 95% confidence intervals for $\widehat{\beta}_\tau$. The gray horizontal line with dashed lines above and below represent $\widehat{\beta}_{\mathrm{OLS}}$ and its 95% confidence interval for a linear model fit to residual data as in panel B. In panel D, $\widehat{\beta}_\tau/\widehat{\beta}_{\mathrm{OLS}}$ versus τ is plotted with vertical bars representing 95% confidence intervals for the ratio. Homogeneous effect sizes across the phenotypic distribution correspond to ratios $\widehat{\beta}_\tau/\widehat{\beta}_{\mathrm{OLS}}$ equal to one for each τ. The gray horizontal lines in panel D bound an equivalence band where the ratio is between $(1/\gamma, \gamma)$ for $\gamma = 1.2$. For ratios with the 95% confidence interval completely above or below the band, the vertical bars are colored red to indicate non-equivalence of the QR and OLS effect sizes and thus heterogeneity of $\widehat{\beta}_\tau$ and the linear predictive value of the PGS across the phenotypic range.

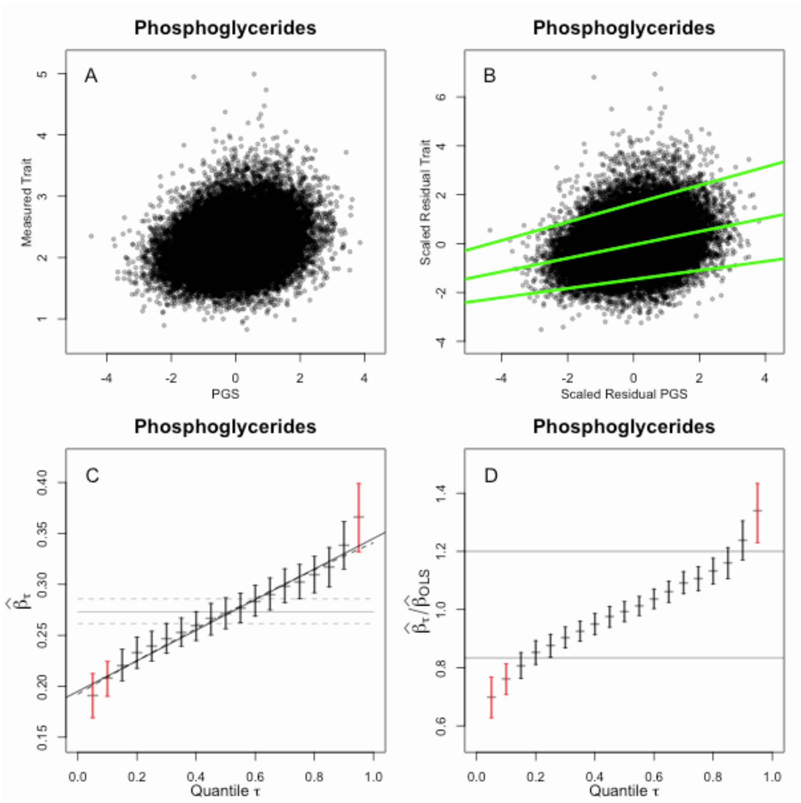

Figure S21. Phosphoglycerides

Panels A and B show scatter plots of the trait (phosphoglycerides) versus polygenic score for participants in the UK Biobank. In panel A and trait and PGS as reported in the data set are plotted. In panel B, residualized and scaled trait and PGS values are used, after projection of age, age^2, sex, age*sex, and ten genetic principal components and rescaling the residuals to mean zero variance one. The green lines show linear models fit to the data using QR at the 95th, 50th, and 5th quantiles of the residual trait distribution. QR is used to estimate quantile specific linear effect sizes $\widehat{\beta}_\tau$ at 19 quantiles τ of the residual trait distribution as in panel B. In panel C, $\widehat{\beta}_\tau$ is plotted against τ, with vertical bars representing 95% confidence intervals for $\widehat{\beta}_\tau$. The gray horizontal line with dashed lines above and below represent $\widehat{\beta}_{\mathrm{OLS}}$ and its 95% confidence interval for a linear model fit to residual data as in panel B. In panel D, $\widehat{\beta}_\tau/\widehat{\beta}_{\mathrm{OLS}}$ versus τ is plotted with vertical bars representing 95% confidence intervals for the ratio. Homogeneous effect sizes across the phenotypic distribution correspond to ratios $\widehat{\beta}_\tau/\widehat{\beta}_{\mathrm{OLS}}$ equal to one for each τ. The gray horizontal lines in panel D bound an equivalence band where the ratio is between $(1/\gamma, \gamma)$ for $\gamma = 1.2$. For ratios with the 95% confidence interval completely above or below the band, the vertical bars are colored red to indicate non-equivalence of the QR and OLS effect sizes and thus heterogeneity of $\widehat{\beta}_\tau$ and the linear predictive value of the PGS across the phenotypic range.

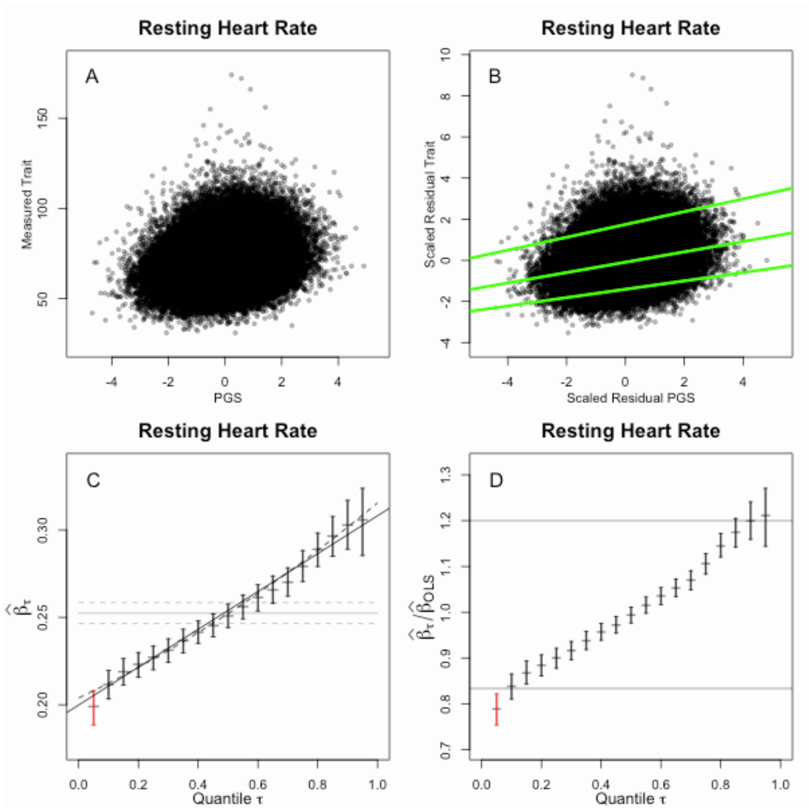

Figure S22. Resting heart rate

Panels A and B show scatter plots of the trait (resting heart rate) versus polygenic score for participants in the UK Biobank. In panel A and trait and PGS as reported in the data set are plotted. In panel B, residualized and scaled trait and PGS values are used, after projection of age, age^2, sex, age*sex, and ten genetic principal components and rescaling the residuals to mean zero variance one. The green lines show linear models fit to the data using QR at the 95th, 50th, and 5th quantiles of the residual trait distribution. QR is used to estimate quantile specific linear effect sizes $\widehat{\beta}_\tau$ at 19 quantiles τ of the residual trait distribution as in panel B. In panel C, $\widehat{\beta}_\tau$ is plotted against τ, with vertical bars representing 95% confidence intervals for $\widehat{\beta}_\tau$. The gray horizontal line with dashed lines above and below represent $\widehat{\beta}_{\text{OLS}}$ and its 95% confidence interval for a linear model fit to residual data as in panel B. In panel D, $\widehat{\beta}_\tau/\widehat{\beta}_{\text{OLS}}$ versus τ is plotted with vertical bars representing 95% confidence intervals for the ratio. Homogeneous effect sizes across the phenotypic distribution correspond to ratios $\widehat{\beta}_\tau/\widehat{\beta}_{\text{OLS}}$ equal to one for each τ. The gray horizontal lines in panel D bound an equivalence band where the ratio is between $(1/\gamma, \gamma)$ for $\gamma = 1.2$. For ratios with the 95% confidence interval completely above or below the band, the vertical bars are colored red to indicate non-equivalence of the QR and OLS effect sizes and thus heterogeneity of $\widehat{\beta}_\tau$ and the linear predictive value of the PGS across the phenotypic range.

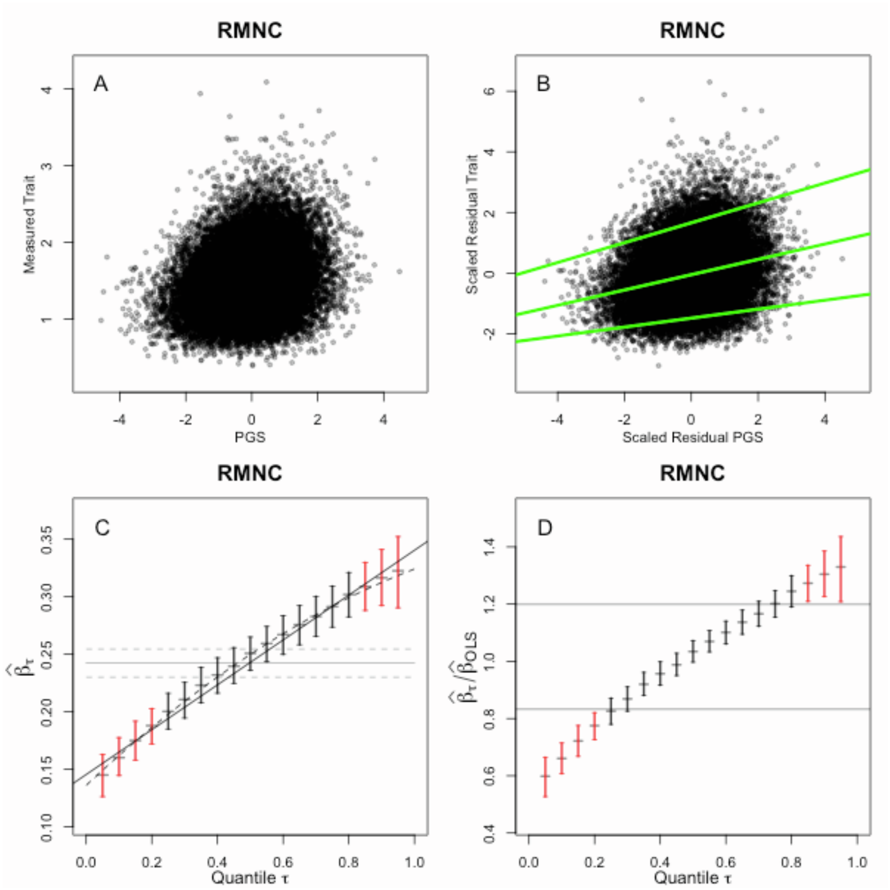

Figure S23. Remnant cholesterol, RMNC

Panels A and B show scatter plots of the trait (remnant cholesterol) versus polygenic score for participants in the UK Biobank. In panel A and trait and PGS as reported in the data set are plotted. In panel B, residualized and scaled trait and PGS values are used, after projection of age, age^2, sex, age*sex, and ten genetic principal components and rescaling the residuals to mean zero variance one. The green lines show linear models fit to the data using QR at the 95th, 50th, and 5th quantiles of the residual trait distribution. QR is used to estimate quantile specific linear effect sizes $\widehat{\beta}_\tau$ at 19 quantiles τ of the residual trait distribution as in panel B. In panel C, $\widehat{\beta}_\tau$ is plotted against τ, with vertical bars representing 95% confidence intervals for $\widehat{\beta}_\tau$. The gray horizontal line with dashed lines above and below represent $\widehat{\beta}_{\mathrm{OLS}}$ and its 95% confidence interval for a linear model fit to residual data as in panel B. In panel D, $\widehat{\beta}_\tau/\widehat{\beta}_{\mathrm{OLS}}$ versus τ is plotted with vertical bars representing 95% confidence intervals for the ratio. Homogeneous effect sizes across the phenotypic distribution correspond to ratios $\widehat{\beta}_\tau/\widehat{\beta}_{\mathrm{OLS}}$ equal to one for each τ. The gray horizontal lines in panel D bound an equivalence band where the ratio is between $(1/\gamma, \gamma)$ for $\gamma = 1.2$. For ratios with the 95% confidence interval completely above or below the band, the vertical bars are colored red to indicate non-equivalence of the QR and OLS effect sizes and thus heterogeneity of $\widehat{\beta}_\tau$ and the linear predictive value of the PGS across the phenotypic range.

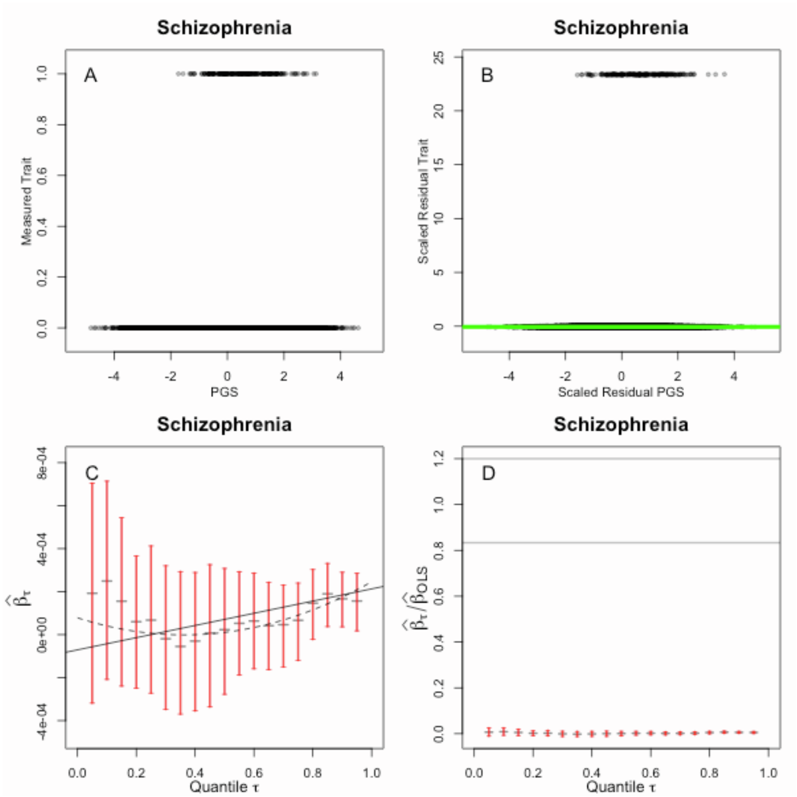

Figure S24. Schizophrenia

Panels A and B show scatter plots of the trait (schizophrenia) versus polygenic score for participants in the UK Biobank. In panel A and trait and PGS as reported in the data set are plotted. In panel B, residualized and scaled trait and PGS values are used, after projection of age, age^2, sex, age*sex, and ten genetic principal components and rescaling the residuals to mean zero variance one. The green lines show linear models fit to the data using QR at the 95th, 50th, and 5th quantiles of the residual trait distribution. QR is used to estimate quantile specific linear effect sizes $\widehat{\beta}_{\tau}$ at 19 quantiles τ of the residual trait distribution as in panel B. In panel C, $\widehat{\beta}_{\tau}$ is plotted against τ, with vertical bars representing 95% confidence intervals for $\widehat{\beta}_{\tau}$. The gray horizontal line with dashed lines above and below represent $\widehat{\beta}_{\mathrm{OLS}}$ and its 95% confidence interval for a linear model fit to residual data as in panel B. In panel D, $\widehat{\beta}_{\tau}/\widehat{\beta}_{\mathrm{OLS}}$ versus τ is plotted with vertical bars representing 95% confidence intervals for the ratio. Homogeneous effect sizes across the phenotypic distribution correspond to ratios $\widehat{\beta}_{\tau}/\widehat{\beta}_{\mathrm{OLS}}$ equal to one for each τ. The gray horizontal lines in panel D bound an equivalence band where the ratio is between $(1/\gamma, \gamma)$ for $\gamma = 1.2$. For ratios with the 95% confidence interval completely above or below the band, the vertical bars are colored red to indicate non-equivalence of the QR and OLS effect sizes and thus heterogeneity of $\widehat{\beta}_{\tau}$ and the linear predictive value of the PGS across the phenotypic range.

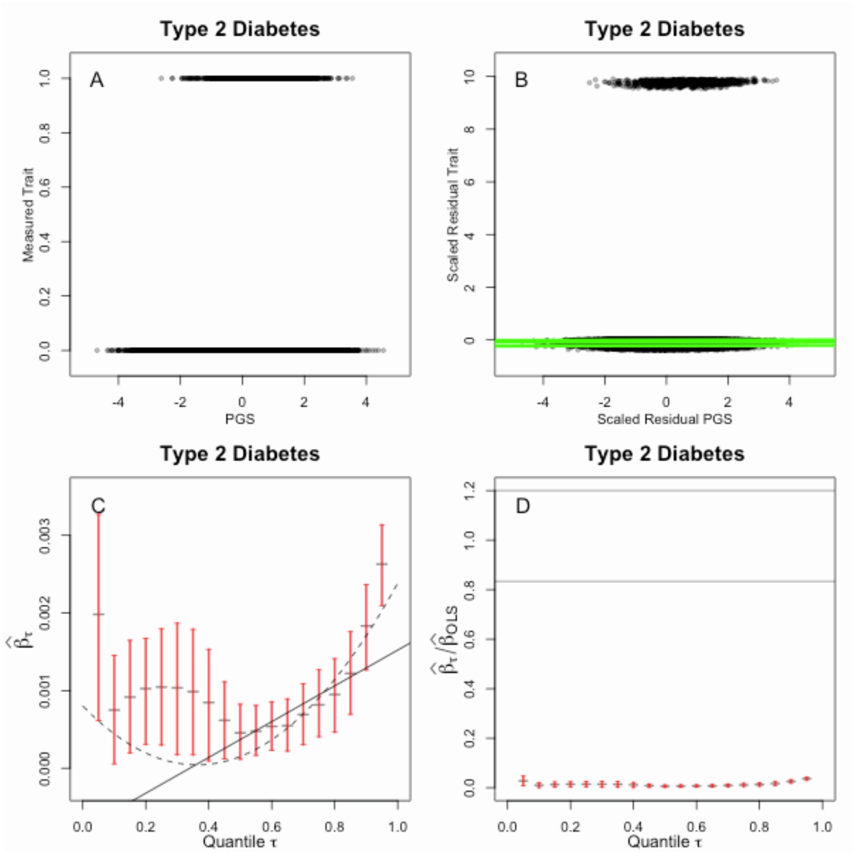

Figure S25. Type 2 diabetes, T2D

Panels A and B show scatter plots of the trait (type 2 diabetes, T2D) versus polygenic score for participants in the UK Biobank. In panel A and trait and PGS as reported in the data set are plotted. In panel B, residualized and scaled trait and PGS values are used, after projection of age, age^2, sex, age*sex, and ten genetic principal components and rescaling the residuals to mean zero variance one. The green lines show linear models fit to the data using QR at the 95th, 50th, and 5th quantiles of the residual trait distribution. QR is used to estimate quantile specific linear effect sizes $\widehat{\beta}_\tau$ at 19 quantiles τ of the residual trait distribution as in panel B. In panel C, $\widehat{\beta}_\tau$ is plotted against τ, with vertical bars representing 95% confidence intervals for $\widehat{\beta}_\tau$. The gray horizontal line with dashed lines above and below represent $\widehat{\beta}_{\mathrm{OLS}}$ and its 95% confidence interval for a linear model fit to residual data as in panel B. In panel D, $\widehat{\beta}_\tau/\widehat{\beta}_{\mathrm{OLS}}$ versus τ is plotted with vertical bars representing 95% confidence intervals for the ratio. Homogeneous effect sizes across the phenotypic distribution correspond to ratios $\widehat{\beta}_\tau/\widehat{\beta}_{\mathrm{OLS}}$ equal to one for each τ. The gray horizontal lines in panel D bound an equivalence band where the ratio is between $(1/\gamma, \gamma)$ for $\gamma = 1.2$. For ratios with the 95% confidence interval completely above or below the band, the vertical bars are colored red to indicate non-equivalence of the QR and OLS effect sizes and thus heterogeneity of $\widehat{\beta}_\tau$ and the linear predictive value of the PGS across the phenotypic range.

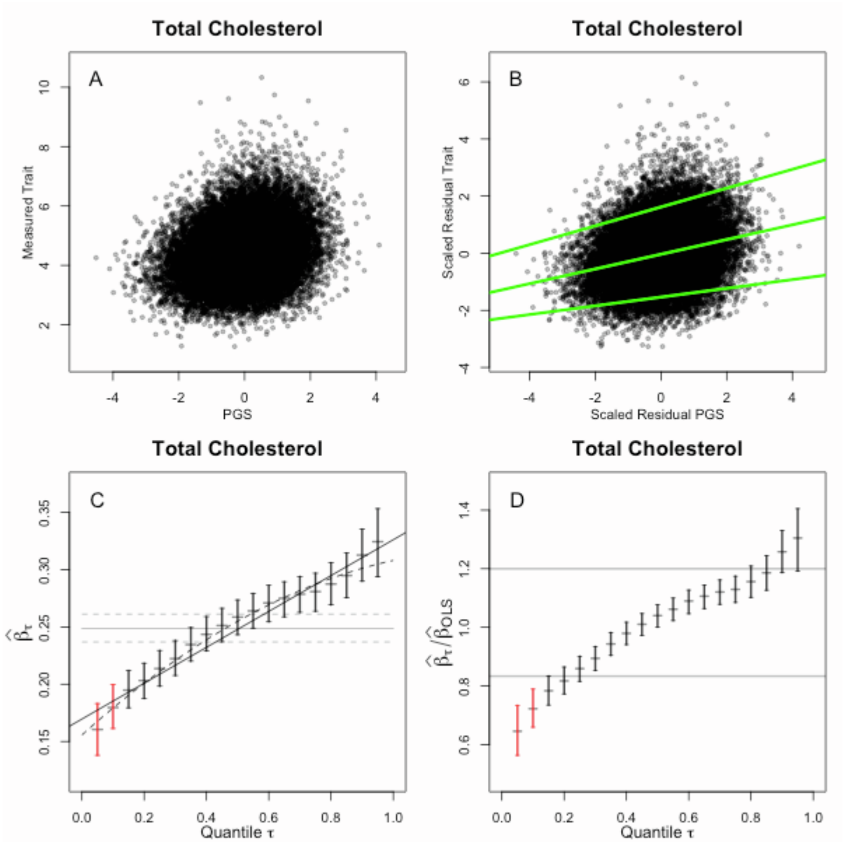

Figure S26. Total cholesterol

Panels A and B show scatter plots of the trait (total cholesterol) versus polygenic score for participants in the UK Biobank. In panel A and trait and PGS as reported in the data set are plotted. In panel B, residualized and scaled trait and PGS values are used, after projection of age, age^2, sex, age*sex, and ten genetic principal components and rescaling the residuals to mean zero variance one. The green lines show linear models fit to the data using QR at the 95th, 50th, and 5th quantiles of the residual trait distribution. QR is used to estimate quantile specific linear effect sizes $\widehat{\beta}_\tau$ at 19 quantiles τ of the residual trait distribution as in panel B. In panel C, $\widehat{\beta}_\tau$ is plotted against τ, with vertical bars representing 95% confidence intervals for $\widehat{\beta}_\tau$. The gray horizontal line with dashed lines above and below represent $\widehat{\beta}_{\mathrm{OLS}}$ and its 95% confidence interval for a linear model fit to residual data as in panel B. In panel D, $\widehat{\beta}_\tau/\widehat{\beta}_{\mathrm{OLS}}$ versus τ is plotted with vertical bars representing 95% confidence intervals for the ratio. Homogeneous effect sizes across the phenotypic distribution correspond to ratios $\widehat{\beta}_\tau/\widehat{\beta}_{\mathrm{OLS}}$ equal to one for each τ. The gray horizontal lines in panel D bound an equivalence band where the ratio is between $(1/\gamma, \gamma)$ for $\gamma = 1.2$. For ratios with the 95% confidence interval completely above or below the band, the vertical bars are colored red to indicate non-equivalence of the QR and OLS effect sizes and thus heterogeneity of $\widehat{\beta}_\tau$ and the linear predictive value of the PGS across the phenotypic range.

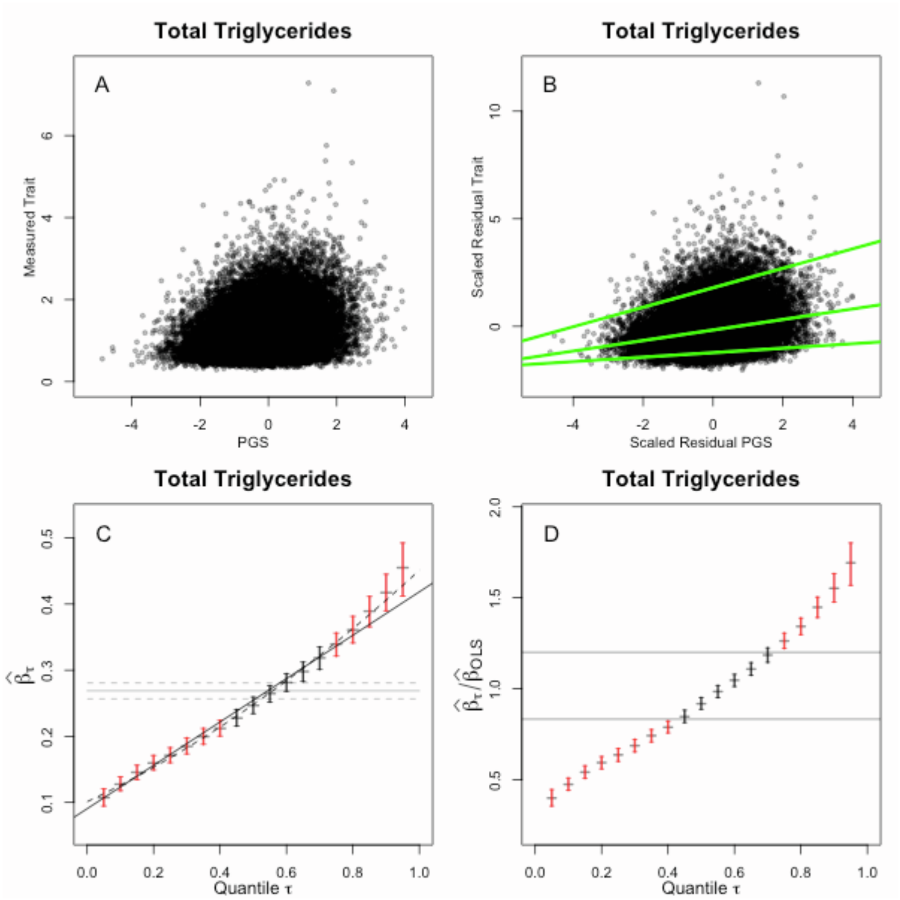

Figure S27. Total triglycerides

Panels A and B show scatter plots of the trait (total triglycerides) versus polygenic score for participants in the UK Biobank. In panel A and trait and PGS as reported in the data set are plotted. In panel B, residualized and scaled trait and PGS values are used, after projection of age, age^2, sex, age*sex, and ten genetic principal components and rescaling the residuals to mean zero variance one. The green lines show linear models fit to the data using QR at the 95th, 50th, and 5th quantiles of the residual trait distribution. QR is used to estimate quantile specific linear effect sizes $\widehat{\beta}_\tau$ at 19 quantiles τ of the residual trait distribution as in panel B. In panel C, $\widehat{\beta}_\tau$ is plotted against τ, with vertical bars representing 95% confidence intervals for $\widehat{\beta}_\tau$. The gray horizontal line with dashed lines above and below represent $\widehat{\beta}_{\text{OLS}}$ and its 95% confidence interval for a linear model fit to residual data as in panel B. In panel D, $\widehat{\beta}_\tau/\widehat{\beta}_{\text{OLS}}$ versus τ is plotted with vertical bars representing 95% confidence intervals for the ratio. Homogeneous effect sizes across the phenotypic distribution correspond to ratios $\widehat{\beta}_\tau/\widehat{\beta}_{\text{OLS}}$ equal to one for each τ. The gray horizontal lines in panel D bound an equivalence band where the ratio is between $(1/\gamma, \gamma)$ for $\gamma = 1.2$. For ratios with the 95% confidence interval completely above or below the band, the vertical bars are colored red to indicate non-equivalence of the QR and OLS effect sizes and thus heterogeneity of $\widehat{\beta}_\tau$ and the linear predictive value of the PGS across the phenotypic range.

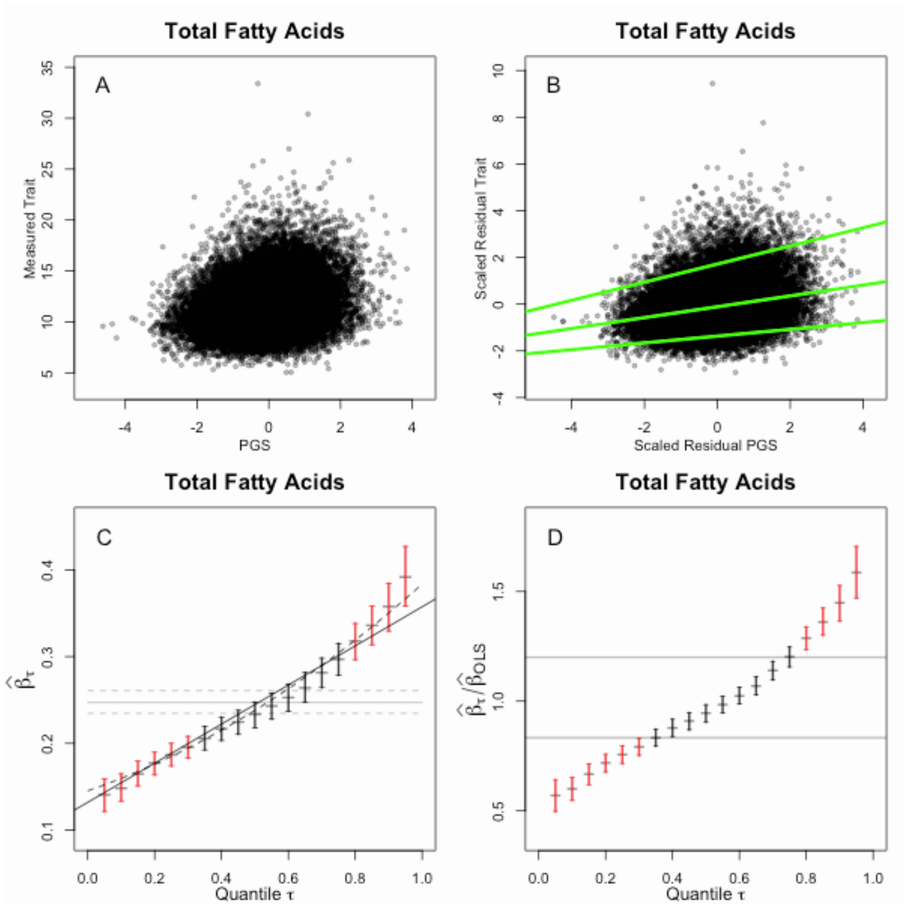

Figure S28. Total fatty acids

Panels A and B show scatter plots of the trait (total fatty acids) versus polygenic score for participants in the UK Biobank. In panel A and trait and PGS as reported in the data set are plotted. In panel B, residualized and scaled trait and PGS values are used, after projection of age, age^2, sex, age*sex, and ten genetic principal components and rescaling the residuals to mean zero variance one. The green lines show linear models fit to the data using QR at the 95th, 50th, and 5th quantiles of the residual trait distribution. QR is used to estimate quantile specific linear effect sizes $\widehat{\beta}_\tau$ at 19 quantiles τ of the residual trait distribution as in panel B. In panel C, $\widehat{\beta}_\tau$ is plotted against τ, with vertical bars representing 95% confidence intervals for $\widehat{\beta}_\tau$. The gray horizontal line with dashed lines above and below represent $\widehat{\beta}_{\mathrm{OLS}}$ and its 95% confidence interval for a linear model fit to residual data as in panel B. In panel D, $\widehat{\beta}_\tau/\widehat{\beta}_{\mathrm{OLS}}$ versus τ is plotted with vertical bars representing 95% confidence intervals for the ratio. Homogeneous effect sizes across the phenotypic distribution correspond to ratios $\widehat{\beta}_\tau/\widehat{\beta}_{\mathrm{OLS}}$ equal to one for each τ. The gray horizontal lines in panel D bound an equivalence band where the ratio is between $(1/\gamma, \gamma)$ for $\gamma = 1.2$. For ratios with the 95% confidence interval completely above or below the band, the vertical bars are colored red to indicate non-equivalence of the QR and OLS effect sizes and thus heterogeneity of $\widehat{\beta}_\tau$ and the linear predictive value of the PGS across the phenotypic range.

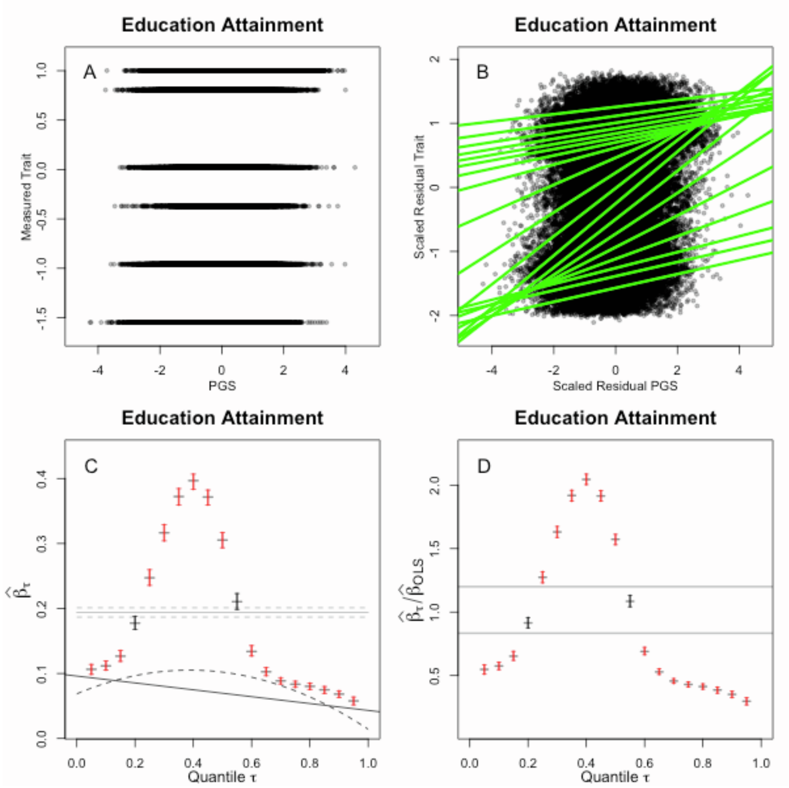

Figure S29. Education Attainment

Educational attainment (EA, years) takes a small number of integer values corresponding to completion of primary school, secondary school, college degree and so on. So, education-years has a distinctly multimodal distribution. These education-years were z-transformed to give the trait values in Panel A. As with case-control traits, the OLS and quantile regression effect estimates for the PGS, $\widehat{\beta}_{\mathrm{OLS}}$ and $\widehat{\beta}_{\tau}$, show heterogeneity of $\widehat{\beta}_{\tau}$ related to the proportions of observations in each mode represented by the horizontal lines in the scatter plot of panel A. Perhaps future work could find a way to quantify and interpret the relatively strong impact of a genetic boost to the liability of moving from one mode of the EA distribution to another for individuals whose non-genetic contributions put them near a threshold between modes. This would generalize the analyses of case-control traits to multimodal traits, but further work is needed to make the quantile-regression and non-equivalence test approach informative for case-control traits as well.

Panels A and B show scatter plots of the trait (education attainment) versus polygenic score for participants in the UK Biobank. In panel A and trait and PGS as reported in the data set are plotted. In panel B, residualized and scaled trait and PGS values are used, after projection of age, age^2, sex, age*sex, and ten genetic principal components and rescaling the residuals to mean zero variance one. QR is used to estimate and plot quantile specific linear models at 19 quantiles τ of the residual trait distribution. In panel C, $\widehat{\beta}_{\tau}$ is plotted against τ, with vertical bars representing 95% confidence intervals for $\widehat{\beta}_{\tau}$. The gray horizontal line with dashed lines above

and below represent $\widehat{\beta}_{\mathrm{OLS}}$ and its 95% confidence interval for a linear model fit to residual data as in panel B. In panel D, $\widehat{\beta}_{\tau}/\widehat{\beta}_{\mathrm{OLS}}$ versus τ is plotted with vertical bars representing 95% confidence intervals for the ratio. Homogeneous effect sizes across the phenotypic distribution correspond to ratios $\widehat{\beta}_{\tau}/\widehat{\beta}_{\mathrm{OLS}}$ equal to one for each τ. The gray horizontal lines in panel D bound an equivalence band where the ratio is between $(1/\gamma, \gamma)$ for $\gamma = 1.2$. For ratios with the 95% confidence interval completely above or below the band, the vertical bars are colored red to indicate non-equivalence of the QR and OLS effect sizes and thus heterogeneity of $\widehat{\beta}_{\tau}$ and the linear predictive value of the PGS across the phenotypic range.

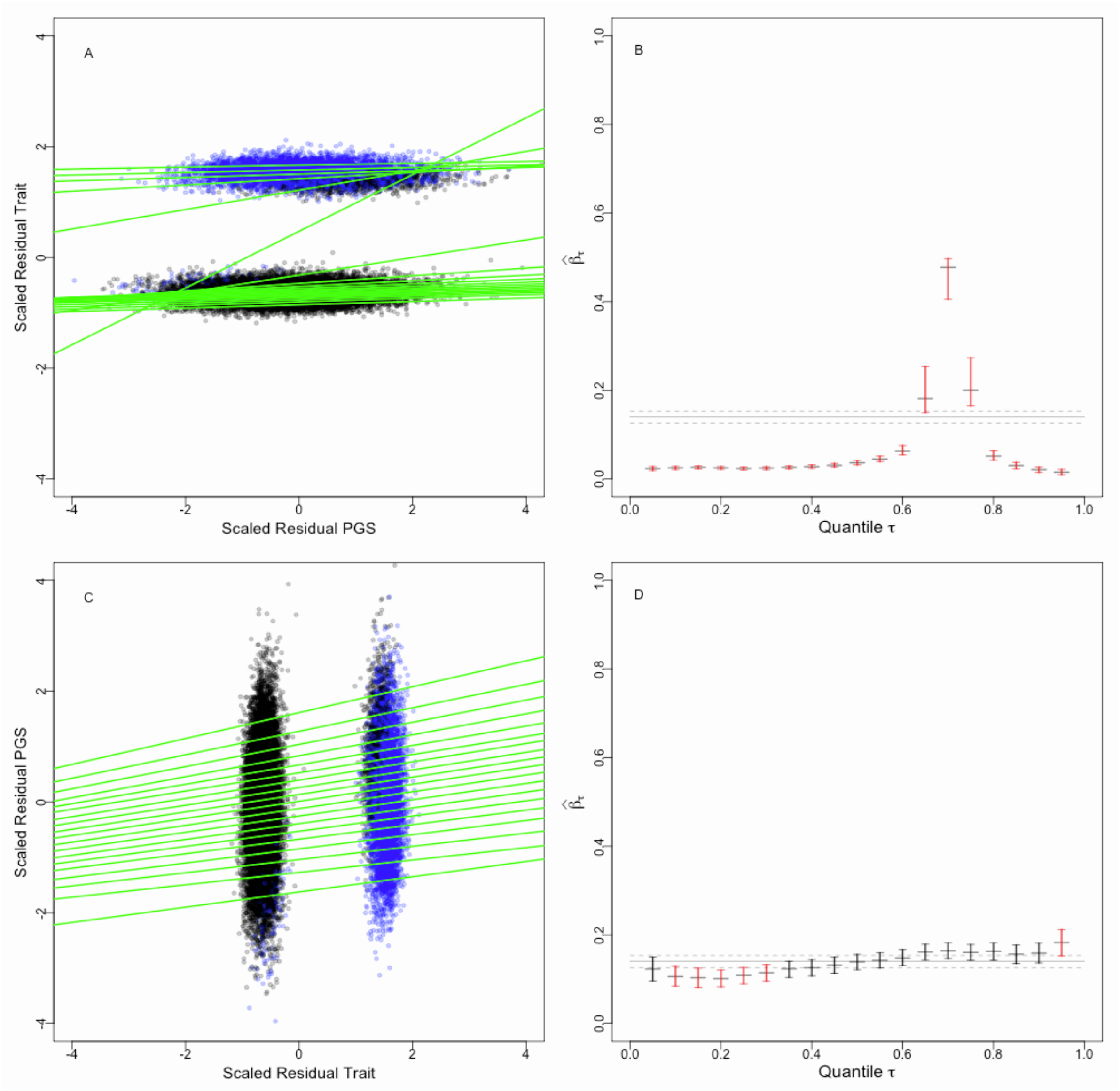

Figure S30. Transposing the analysis for case-control data

Figures S30 and S31 are results from analysis of a case control data set corresponding to the data of Figure 4P (S31) and another bimodal dataset with better separated modes (S30, $\gamma = 5$). Here the continuous trait analyzed for Figure 4A and 4B was treated as a liability for the case-control trait in S31. Figure S30 is for a trait with better separated liabilities for the cases and controls. Panel S30B shows a spike at quantile τ=0.7, corresponding to the 0.7 control proportion in the simulated dataset or the 1-0.7=0.3 case proportion.

Panels S30C and S30D show the same data as in panels A and B, but with the axes transposed. With these scales, quantile regression models the quantiles of the continuous PGS distribution rather than the dichotomous trait. Comparing panels S30B and D, or S30D and S31D, we don't see any easy way to interpret these results in terms of exposure or interactions

as we have done for other deviations from homogeneity. We do not see any way to interpret the results of the transposed analysis in terms of case-control proportions either.

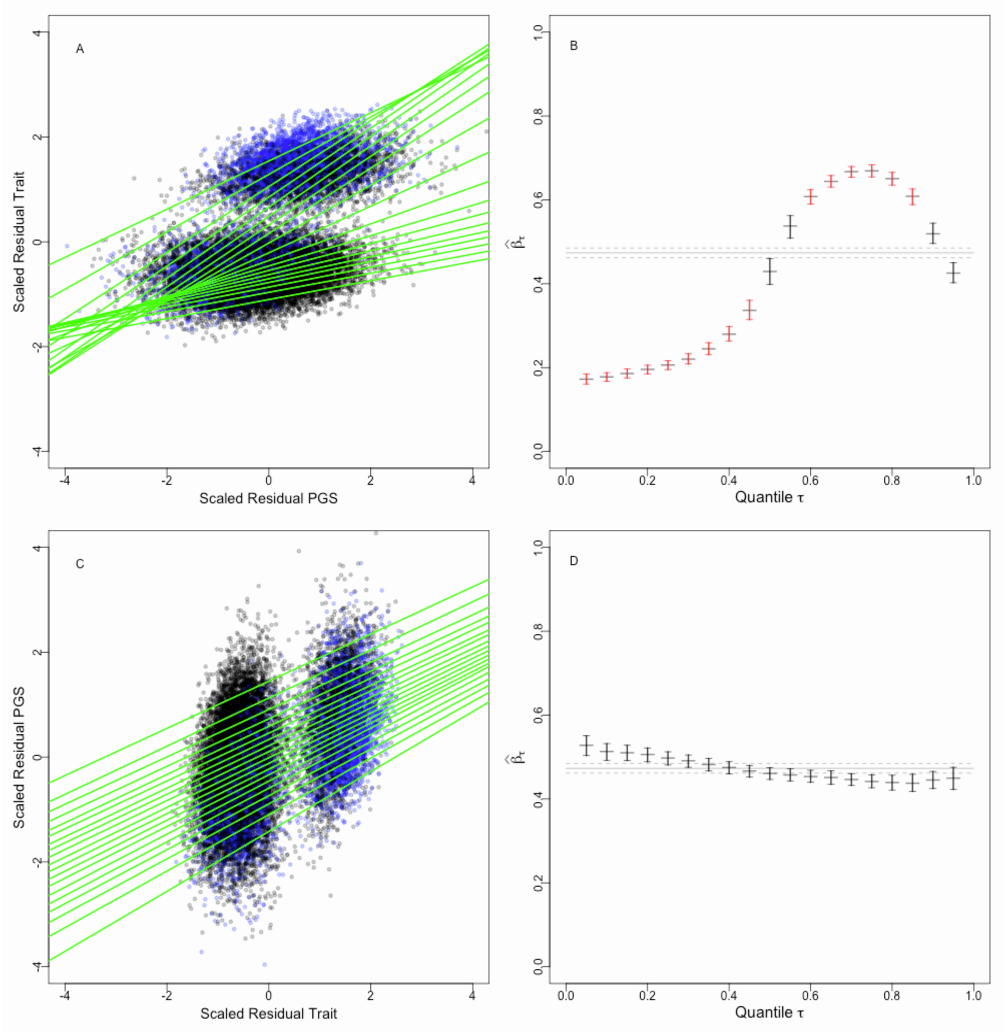

Figure S31. Transposing the analysis for bimodal data

Figures S30 and S31 are results from analysis of a case control data set corresponding to the data of Figure 4P (S31) and another bimodal dataset with better separated modes (S30, $\gamma = 5$). Here the continuous trait analyzed for Figure 4A and 4B was treated as a liability for the case-control trait in S31. Figure S30 is for a trait with better separated liabilities for the cases and controls. Panel S31B shows a mode at quantile τ=0.7, corresponding to the 0.7 control proportion in the simulated dataset or the 1-0.7=0.3 case proportion. This mode is broader than the one in panel S30B due to the less well-separated modes in Figure S31 than in S29.

Panels S31C and S31D show the same data as in panels A and B, but with the axes transposed. With these scales, quantile regression models the quantiles of the continuous PGS distribution rather than the dichotomous trait. Comparing panels S31B and D, or S31D and

S30D, we don't see any easy way to interpret these results in terms of exposure or interactions as we have done for other deviations from homogeneity. We do not see any way to interpret the results of the transposed analysis in terms of case-control proportions either.

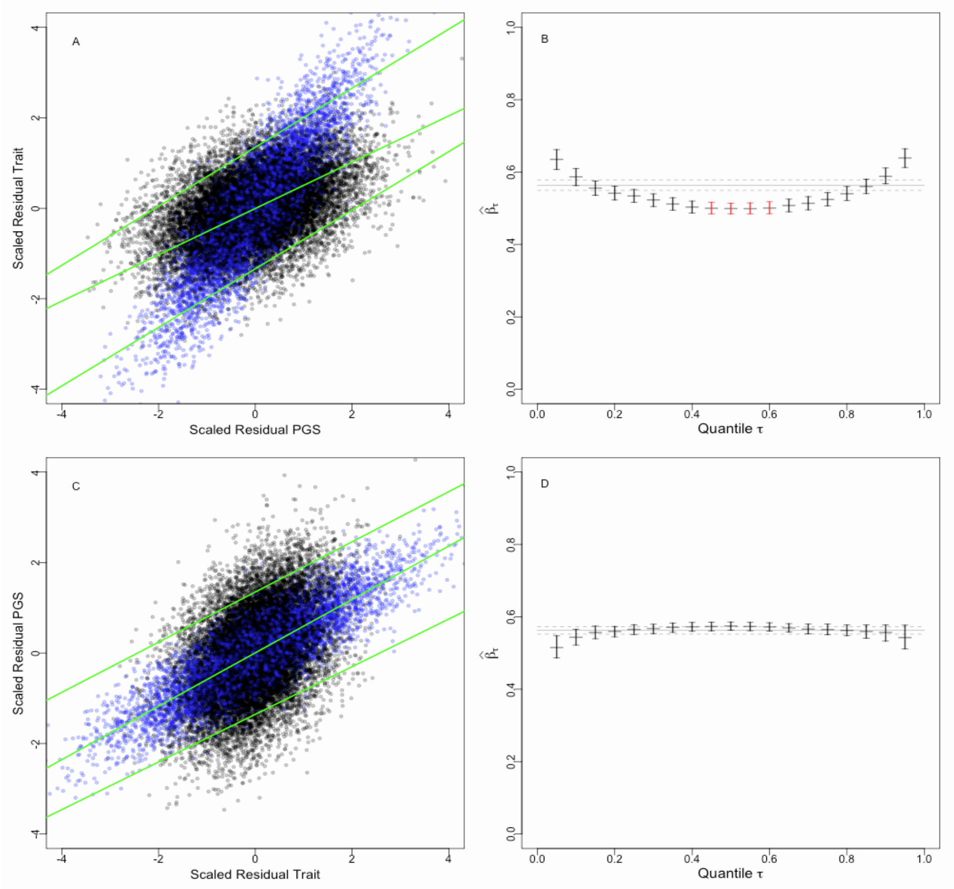

Figure S32. Transposing the analysis for the $G \times E$ model

For S32 and S33 we compare the standard analyses to the transposed analysis for continuous traits. In the residual analysis we use (residual trait vs residual PGS) with rescaled residuals, the OLS regression coefficient is also the correlation. In the transposed analysis, the OLS regression coefficient is again the correlation The quantile-specific linear model effect sizes are then analogous to correlations or OLS effect sizes. Specifically, for the analyses in this paper, $\widehat{\beta}_{\tau}$ is the slope of a trend line through the τth percentile of residual outcome vs residual PGS. The simulations for Figure 4 suggest how patterns in plots of $\widehat{\beta}_{\tau}$ versus τ reflect the data generating model – directions and strengths of GxE interactions and E main effects, and the proportion PrE of observations having a dichotomous exposure. For a "turned around analysis", $\widehat{\beta}_{\tau}$ is the slope of a trend line through the τth percentile of residual PGS vs residual PGS. In

supplemental figures S32 and S33, data with pronounced GxE interactions is analyzed by our standard trait-vs-PGS approach in Panels A and B, then by the turned-around method for Panels C and D. For the standard analysis, significant non-equivalence of $\widehat{\beta}_\tau$ and the OLS effect size is shown, and the patterns on $\widehat{\beta}_\tau$ versus τ suggest a GxE interaction with negligible E main effect for S32, and a GxE interaction with an E main effect both increasing trait values in the exposed observations and an exposed proportion around 0.3. For the turned around analyses, no $\widehat{\beta}_\tau$ are flagged as non-equivalent to the OLS estimate, and we don't see any we cannot see obvious patterns in the $\widehat{\beta}_\tau$ versus τ to give insight into the data generating model. There may be some other informative way to use quantile regression to characterize disease polygenic risk scores

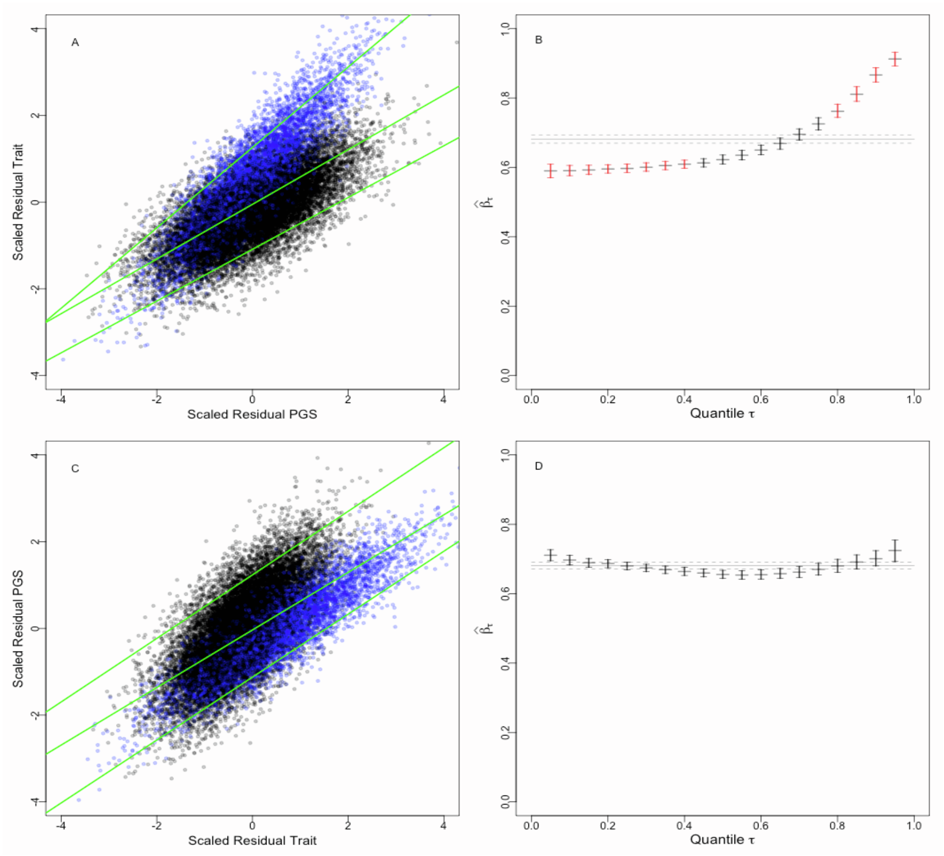

Figure S33. Transposing the analysis for the $G \times E$ model with an E main effect

For S32 and S33 we compare the standard analyses to the transposed analysis for continuous traits. In the residual analysis we use (residual trait vs residual PGS) with rescaled residuals, the OLS regression coefficient is also the correlation. In the transposed analysis, the OLS regression coefficient is again the correlation The quantile-specific linear model effect sizes are then analogous to correlations or OLS effect sizes. Specifically, for the analyses in this paper, $\widehat{\beta}_{\tau}$ is the slope of a trend line through the τth percentile of residual outcome vs residual PGS. The simulations for Figure 4 suggest how patterns in plots of $\widehat{\beta}_{\tau}$ versus τ reflect the data generating model – directions and strengths of GxE interactions and E main effects, and the proportion PrE of observations having a dichotomous exposure. For a "turned around analysis", $\widehat{\beta}_{\tau}$ is the slope of a trend line through the τth percentile of residual PGS vs residual PGS. In supplemental figures S32 and S33, data with pronounced GxE interactions is analyzed by our standard trait-vs-PGS approach in Panels A and B, then by the turned-around method for Panels C and D. For the standard analysis, significant non-equivalence of $\widehat{\beta}_{\tau}$ and the OLS effect size is shown, and the patterns on $\widehat{\beta}_{\tau}$ versus τ suggest a GxE interaction with a substantial E main effect for S33, with the GxE interaction and E main effect both increasing

trait values in the exposed observations and an exposed proportion around 0.3. For the turned around analyses, no $\widehat{\beta}_\tau$ are flagged as non-equivalent to the OLS estimate, and we don't see any we cannot see obvious patterns in the $\widehat{\beta}_\tau$ versus τ to give insight into the data generating model. There may be some other informative way to use quantile regression to characterize disease polygenic risk scores

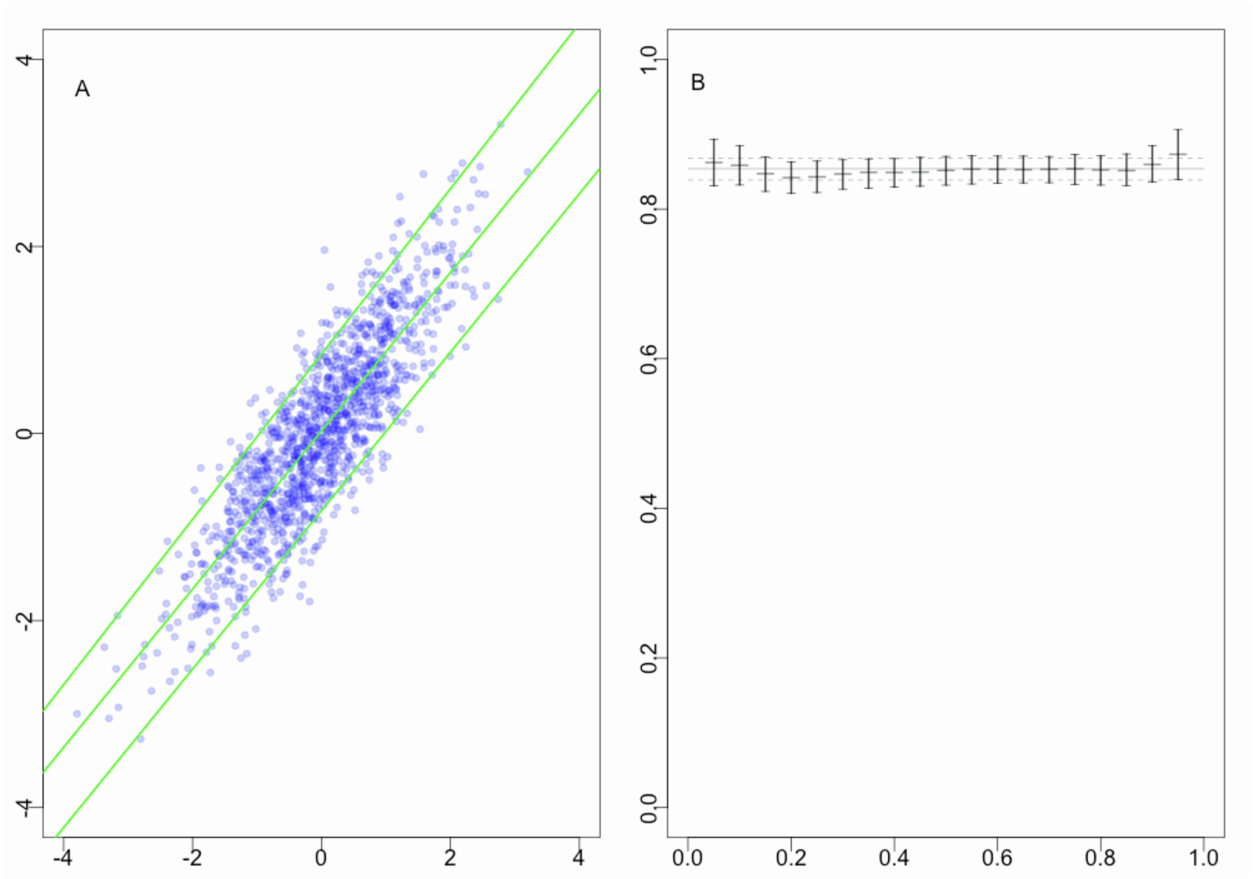

Figure S34. Reference Polygenic Scores

Supplemental Figure S34 illustrates the effect of using an ‘off-the-shelf’ polygenic score (rather than one generated from the same data) For Figure 5 Panels E-H, the GWAS and PGS development was done separately in the exposed (5E and 5F) and non-exposed (5G and 5H) strata, however, using a polygenic score developed on a mixed set of exposed and unexposed observations but following with the quantile regression and non-equivalence tests in a single partition also results in homogeneous $\widehat{\beta}_{\tau}$ in the stratum specific analyses. So, the confirmatory analyses for checking the contribution of a particular exposure to an observed pattern of heterogeneous $\widehat{\beta}_{\tau}$, does not require access to the genetic source data for a new GWAS run in the stratum of interest, and development of a new PGS. However, removal of one source of trait heterogeneity and stronger PGS predictive value in one stratum suggests further analyses of each stratum separately may be informative.

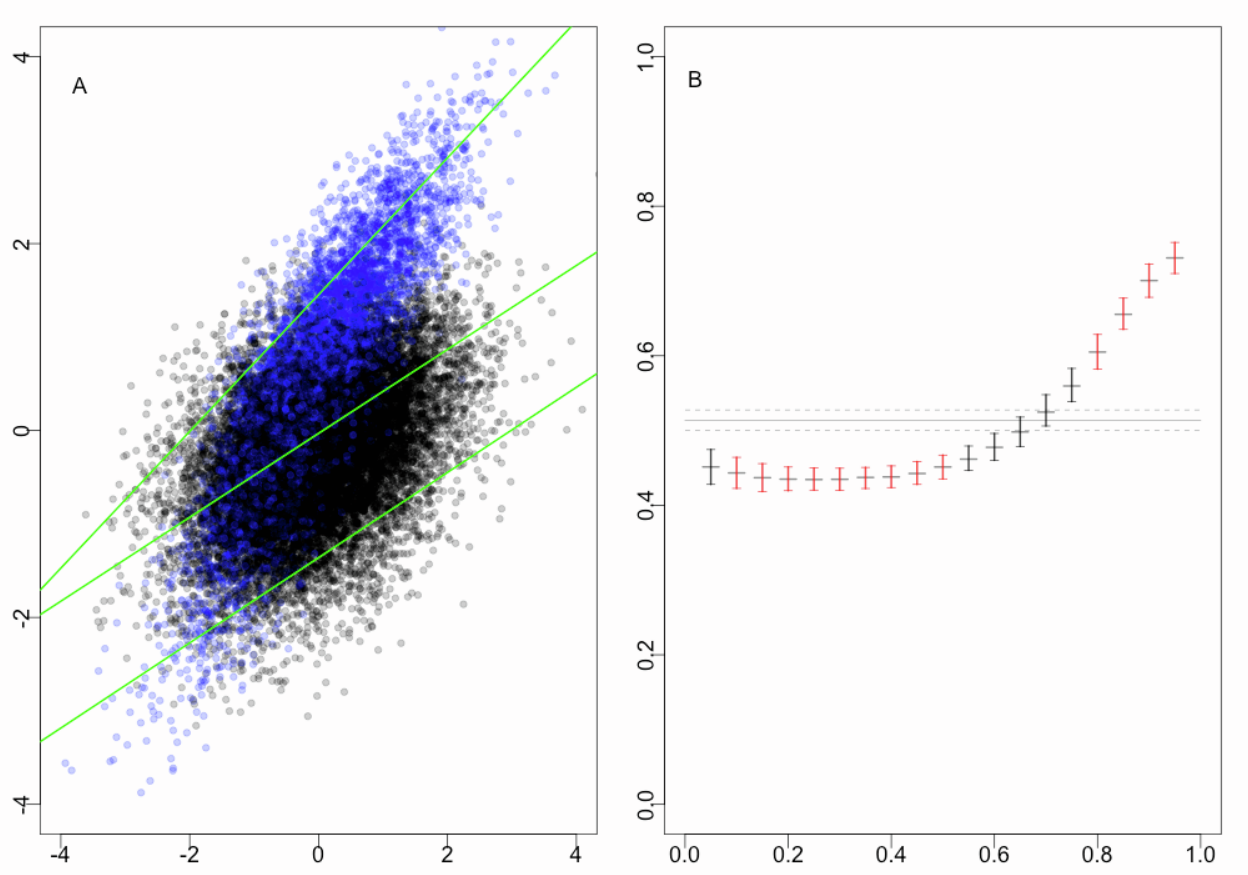

Figure S35. Data transformation

In Figure S35, the same data that was analyzed for Figure 5 was used. The contrast between Figure 5 Panels A and B and Figure S35, is that to generate the later figure, the trait was quantile normalized before analysis. The trait was quantile normalized before the GWAS, so SNP effect sizes and the PGS do not have the same interpretation as in the non-transformed analyses in Figure 5A and 5B, but the plots of $\widehat{\beta}_{\tau}$ versus quantile τ are qualitatively the same for the analyses of the non-transformed and transformed data (uniformly low $\widehat{\beta}_{\tau}$ values at low quantiles below 0.5, sloping up to higher values at higher quantiles, crossing the OLS effect size estimate around 0.75) . Here, the data generating model corresponds to the non-transformed analysis, which may explain the somewhat higher power for the non-equivalence tests using the non-transformed data – more $\widehat{\beta}_{\tau}$ quantiles are significantly non-equivalent to the OLS estimate with the non-transformed analysis.

Table S1. Quantile-specific and OLS predictive R^2

Trait	Trait code	R2 OLs	R2 min	R2 max	R2 min/ R2 OLS	R2 max / R2 OLS	Max $\widehat{\beta}_\tau/\widehat{\beta}_{\mathrm{OLS}}$	Min $\widehat{\beta}_\tau/\widehat{\beta}_{\mathrm{OLS}}$
Age At Menopause	AAM	0.053	0.028	0.111	0.523	2.078	0.723	1.442
Apolipoprotein A1	APOEA	0.097	0.054	0.191	0.559	1.972	0.748	1.404
Apolipoprotein B	APOEB	0.107	0.048	0.211	0.453	1.966	0.673	1.402
Body Mass Index	BMI	0.119	0.033	0.355	0.273	2.973	0.523	1.724
Calcium	CAL	0.065	0.056	0.075	0.864	1.160	0.930	1.077
Docosahexaenoic Acid	DOA	0.050	0.035	0.083	0.699	1.651	0.836	1.285
Estimated Bone Mineral Density T Score	EBMDT	0.016	0.001	0.067	0.042	4.172	0.204	2.043
Estimated Glomerular Filtration Rate (Creatinine Based)	EGCR	0.040	0.057	0.026	1.422	0.641	1.192	0.801
Estimated Glomerular Filtration Rate (Cystatin Based)	EGCY	0.073	0.124	0.043	1.701	0.589	1.304	0.768
Glycated Haemoglobin	HBA1C	0.058	0.039	0.197	0.673	3.409	0.821	1.846
Height	HEIGHT	0.302	0.264	0.323	0.875	1.071	0.936	1.035
High Density Lipoprotein Cholesterol	HDL	0.157	0.076	0.311	0.487	1.984	0.698	1.409
Intraocular Pressure	IOP	0.032	0.019	0.045	0.598	1.400	0.774	1.183
Low Density Lipoprotein Cholesterol	LDL	0.094	0.026	0.195	0.282	2.077	0.531	1.441
Omega 3 Fatty Acids	OTFA	0.075	0.040	0.138	0.529	1.831	0.727	1.353
Omega 6 Fatty Acids	OSFA	0.062	0.021	0.143	0.334	2.292	0.578	1.514
Phosphatidylcholines	PDCL	0.083	0.042	0.150	0.507	1.792	0.712	1.339
Phosphoglycerides	PHG	0.075	0.034	0.141	0.459	1.888	0.677	1.374
Polyunsaturated Fatty Acids	PFA	0.070	0.029	0.136	0.417	1.950	0.646	1.396

Remnant Cholesterol (Non Hdl, Non Ldl Cholesterol)	RMNC	0.059	0.022	0.107	0.372	1.827	0.610	1.352
Resting Heart Rate	RHR	0.065	0.041	0.097	0.640	1.502	0.800	1.226
Sphingomyelins	SGM	0.076	0.039	0.134	0.517	1.772	0.719	1.331
Total Cholesterol	TCH	0.062	0.023	0.109	0.381	1.763	0.617	1.328
Total Fatty Acids	TFA	0.061	0.021	0.151	0.343	2.452	0.586	1.566
Total Triglycerides	TTG	0.072	0.011	0.206	0.150	2.855	0.388	1.690
